# Iminothioindoxyl as a molecular photoswitch with 100 nm band separation in the visible range

**DOI:** 10.1038/s41467-019-10251-8

**Published:** 2019-06-03

**Authors:** Mark W. H. Hoorens, Miroslav Medved’, Adèle D. Laurent, Mariangela Di Donato, Samuele Fanetti, Laura Slappendel, Michiel Hilbers, Ben L Feringa, Wybren Jan Buma, Wiktor Szymanski

**Affiliations:** 1 Department of Radiology, Medical Imaging Center, University Medical Center Groningen, University of Groningen, Hanzeplein 1, 9713 GZ Groningen, The Netherlands; 2 Faculty of Science and Engineering, Centre for Systems Chemistry, Stratingh Institute for Chemistry, University of Groningen, Nijenborgh 7, 9747 AG Groningen, The Netherlands; 3 Faculty of Science, Regional Centre of Advanced Technologies and Materials, Palacký University in Olomouc, Šlechtitelů 27, CZ-771 46 Olomouc, Czech Republic; 4 Faculty of Natural Sciences, Department of Chemistry, Matej Bel University, Tajovského 40, SK-97400 Banská Bystrica, Slovak Republic; 5 University of Nantes, CEISAM UMR CNRS 6230, BP 92208 2 Rue de la Houssiniere, 44322, Cedex 3 Nantes, France; 6 European Laboratory for Non Linear Spectroscopy (LENS) via N. Carrara 1, 50019 Sesto Fiorentino, Italy; 7 INO, Istituto Nazionale di Ottica, Largo Fermi 6, 50125 Firenze, Italy; 8 Van’t Hoff Institute for Molecular Sciences, University of Amsterdam, Science Park 904, 1098 XH Amsterdam, The Netherlands; 9 Institute for Molecules and Materials, FELIX Laboratory, Radboud University, Toernooiveld 7c, 6525 ED Nijmegen, The Netherlands; 10 Department of Chemistry ‘Ugo Shiff’, University of Florence, via della Lastruccia 3-13, 50019 Sesto Fiorentino (FI), Italy

**Keywords:** Synthetic chemistry methodology, Light harvesting, Chemical physics

## Abstract

Light is an exceptional external stimulus for establishing precise control over the properties and functions of chemical and biological systems, which is enabled through the use of molecular photoswitches. Ideal photoswitches are operated with visible light only, show large separation of absorption bands and are functional in various solvents including water, posing an unmet challenge. Here we show a class of fully-visible-light-operated molecular photoswitches, Iminothioindoxyls (ITIs) that meet these requirements. ITIs show a band separation of over 100 nm, isomerize on picosecond time scale and thermally relax on millisecond time scale. Using a combination of advanced spectroscopic and computational techniques, we provide the rationale for the switching behavior of ITIs and the influence of structural modifications and environment, including aqueous solution, on their photochemical properties. This research paves the way for the development of improved photo-controlled systems for a wide variety of applications that require fast responsive functions.

## Abstract (web summary)

The design of photoswitches which operate in the visible light regime, show a large separation of absorption bands and are functional in various solvents is challenging. Here the authors report Iminothioindoxyls as visible-light operated photoswitches with a band separation of 100 nm.

## Introduction

There is currently a growing interest in the development of responsive functional systems that can be controlled with light, which is a powerful, non-invasive external stimulus. Photochemical control is exerted at the molecular level through light-responsive chemical structures, i.e. photoswitches, which usually have two isomers that can be reversibly interconverted upon irradiation at different wavelengths^1,2^. Often, one of those isomers is less stable and thermally converts back over time to the stable isomer. The two photo-isomers of the switch differ in structure and chemical properties, which enables photochemical control of the systems in which they are embedded^1–4^, including drugs and their protein targets^5,6^, drug delivery systems^7,8^, the function of hydrogels in regenerative medicine^9^, the conformation of peptides^10^ and nucleotides^11^. Fascinating applications in bio-imaging^12,13^ and vision restoration^14^ are also emerging. However, for these applications, only a limited number of photoswitches is available, each with its own scope and limitations.

The selectivity in addressing the photoswitchable component in a complex functional system is crucial for its application. Because many molecular components of such systems absorb light in the UV range, a major challenge is to achieve selective switching through the design of photoswitches that can be operated in both directions using visible light. For example, in the emerging area of photopharmacology^5,6,15–17^, visible light switching is crucial to enable deep tissue penetration, especially in the 650–900 nm range^3^. However, most of the commonly used switches, such as diarylethenes, spiropyrans, Donor-Acceptor Stenhouse Adducts (DASAs) and fulgides, do not show absorption bands of both photo-isomers in the visible light region^2,18,19^. For switches that can be operated in both directions in the visible range, such as substituted azobenzenes^1^ and indigoids such as indigo^20^ and hemithioindigos^21,22^, the band separation becomes a challenge, limiting their selective bidirectional photoisomerization. Only recently, this problem has been addressed for azobenzenes by the groups of Woolley and Hecht, who developed fully-visible-light-responsive azobenzenes^1,3,23^, which - despite lower water solubility and challenging synthesis - have been successfully used for biological applications^24–26^. Yet, the band separation to achieve selectivity remains an unmet challenge.

In our continuous efforts to expand the limited repertoire of molecular photoswitches, we further focused on several characteristics that they should possess, besides the visible light operation with large band separation. Firstly, the photoswitch should be a small structural motif, in order to introduce it into the structure of a compound or material while affecting its original design only minimally. Secondly, it should be synthetically readily accessible. Thirdly, the parameters that control the rate of the thermal back isomerization reaction should be understood. Finally, for biological applications, the photoswitch should be able to operate under aqueous conditions. So far, realizing all these requirements in one molecular photoswitch has not been achieved.

Here we present the design, synthesis and evaluation of a class of photoswitches, which combine the photochromic dyes thioindigo and azobenzene into a photoswitch called Iminothioindoxyl (ITI). We demonstrate fully-visible (blue/orange) light switching of ITI in either direction and a large band separation between both isomers of over 100 nm. We furthermore investigate, through a comprehensive combination of synthesis, spectroscopy and theoretical calculations, the influence of the environment and chemical substitution on the switching process and re-isomerization speed of ITI. Also, we demonstrate that these spectacular photochemical properties are retained for aqueous solutions, which opens opportunities for applying ITI for reversibly controlling biological systems.

## Results

### Design and synthesis of ITI

The design of iminothioindoxyl (ITI) is inspired by the structure of the visible-light-responsive molecular photoswitch hemithioindigo (HTI)^21,22^, which consists of half a thioindigo and half a stilbene moiety, featuring a photo-isomerizable C=C double bond. Yet, photo-isomerization is not limited to C=C double bonds. In particular, C=N photo-isomerization has recently attracted attention in designing molecular photoswitches^27–31^. Based on that, we envisioned that a molecular architecture combining azobenzene and indigoid photochromic unit could also show switching properties.

Already in the early 1900s, the chemical structures of ITI and similar compounds have been reported as dyes^32^. Back in 1910, Rudolf Pummerer reported the one-step synthesis of ITI by the condensation of thioindoxyl with nitrosobenzene^33^. Nearly 100 years later, Soeta reported the synthesis of the same chemical structure using a Passerini-type [4 + 1] cycloaddition^34^, also confirming through X-ray crystallography that the *Z*-form is the thermodynamically stable one. However, to the best of our knowledge, the behavior of these structures as molecular photoswitches has not been explored so far.

**Fig. 1:**
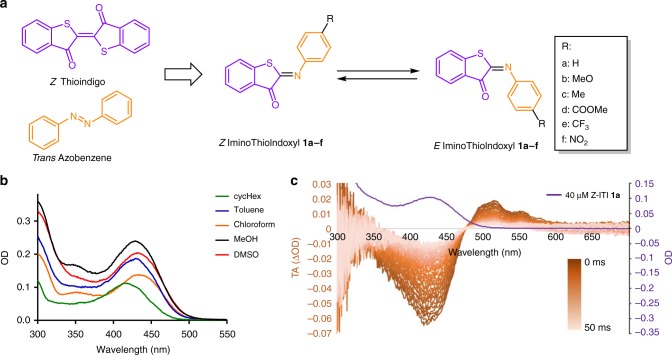
Design and absorption of ITI. **a** The structure of Iminothioindoxyl (ITI) is a hybrid of thioindigo (purple) and azobenzene (orange). The R group indicates various substituents to study the electronic effects on the photochemical properties. **b** Absorption spectra of 40 μM ITI **1a** in cyclohexane, toluene, chloroform, MeOH and DMSO. **c** Millisecond transient absorption of 400 μM ITI **1a** in MeOH at room temperature. The sample was irradiated with a 430 nm light pulse, upon which the spectrum was recorded with 1 ms delay steps. The color bar represents increased delay of transient absorption spectroscopy and the purple line represents the spectrum of 40 μM of *Z*-ITI **1a** in MeOH after thermal equilibration

Here, we report the synthesis of six ITIs **1a-f** by the condensation of thioindoxyl with substituted nitrosobenzene derivatives (Supplementary Fig. 1). Besides unsubstituted ITI **1a**, two electron donating substituents (**1b, 1c**) and three electron withdrawing substituents (**1d–1f**) were placed at the R-position (Fig. 1a) to determine the influence of different substitution patterns on the photochemical properties of ITI, including absorption maxima and switching properties. Full experimental procedures and characterization is reported in Supplementary Methods and Supplementary Fig. 1–21.

### Solvent effects of ITI photo-isomerization

**Fig. 2:**
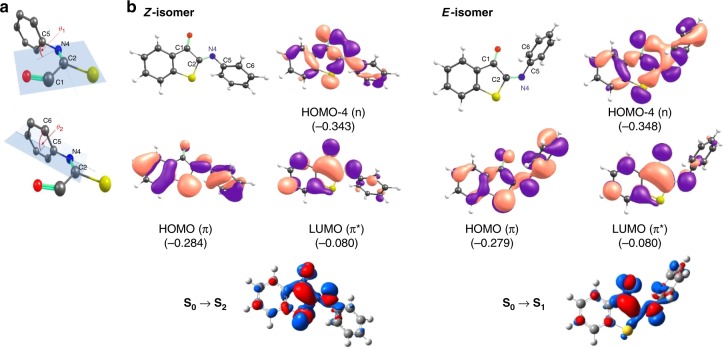
Computational studies on solvents on ITI photo-isomerization. **a** Angles θ_1_ (top) and θ_2_ (bottom). **b** Structures of the *Z* and *E* forms of ITI **1a** in MeOH with the numbering of atoms in the central part of a molecule, molecular orbitals involved in the observed electronic transition (energies in Hartrees) and electron density difference (EDD) plots showing the decrease (blue) and increase (red) of the electron density upon excitation obtained at the SMD-TD-M06-2X/6-311++G(2df,2p)//SMD-M06-2X/6-31+G(d) level of theory

To determine the influence of the medium on the photochemical properties of unsubstituted ITI **1a**, absorption spectra were recorded in five solvents with different polarity (Fig. 1b, Table 1). In all solvents, the *Z*-isomer of ITI has an absorption band in the 400–500 nm region, with only limited solvatochromism. No clear correlation between solvent polarity and *λ*_max,Z_ was observed within the group of polar solvents examined (Supplementary Fig. 61), similarly to the hemithioindigo switch^35^. Time-dependent density functional theory (TD-DFT) calculations at the TD-M06–2X/6–311++G(2df,2p) level^36,37^, in combination with the universal solvation model based on density (SMD)^38^ (see Supplementary Information) predicted that the band corresponds to the S_0_ → S_2_ transition with prevailing π→π* (HOMO → LUMO) character, while the first excited state S_1_ is a mixed state with a significant n→π* (HOMO-4 → LUMO) contribution (Supplementary Note 2, Supplementary Tables 1–3, Supplementary Figs. 23–25). In fact, due to twisting of the phenyl group out of the molecular plane (see *θ*_2_ in Fig. 2a), both excited states  are partially mixed.

**Table 1:** Computational studies on solvents on ITI photo-isomerization

	*Z*-isomer				
Solvent (ε_r_)	*λ*_max,Z_ (nm)		Transition		
	exp.	calc.	ππ*/nπ*	*θ*_1_(C1-C2-N4-C5)/*θ*_2_(C2-N4-C5-C6)	Δμ_ES-GS,Z_(D)
Cyclohexane (2.02)	416	373	S_0_→S_2_ 0.61/0.18	179.8/49.8	1.77
Toluene (2.37)	430	374	S_0_→S_2_ 0.61/0.20	179.8/50.2	1.86
CHCl_**3**_ (4.71)	435	378	S_0_→S_2_ 0.61/0.20	180.0/51.4	2.26
MeOH (32.61)	429	398	S_0_→S_1_ 0.58/0.34	−179.8/54.1	1.55
DMSO (46.83)	432	379	S_0_→S_2_ 0.61/0.24	180.0/54.1	2.82

	*E*-isomer				
Solvent (ε_r_)	*λ*_max,E_ (nm)		Transition		
	exp.	calc.	ππ*/nπ*	*θ*_1_(C1-C2-N4-C5)/*θ*_2_(C2-N4-C5-C6)	Δμ_ES-GS,E_(D)
Cyclohexane (2.02)	**517**, 554	520	S_0_→S_1_ 0.55/0.31	9.5/60.6	−2.85
Toluene (2.37)	**510**, 551	519	S_0_→S_1_ 0.55/0.31	9.5/61.7	−2.96
CHCl_3_ (4.71)	**506**, 549,	513	S_0_→S_1_ 0.55/0.31	9.3/62.9	−3.48
MeOH (32.61)	**515**, 552	505	S_0_→S_1_ 0.54/0.30	9.3/66.0	−4.17
DMSO (46.83)	**514**, 553	503	S_0_→S_1_ 0.55/0.31	9.0/66.9	−4.05

						Transition state	
Solvent (ε_r_)	Δ*λ*_max_ (nm)		*t*_1/2_ (ms)	$\Delta G_{Z - E}^\#$ (kcal/mol)			
	exp.	calc.	exp.	exp.	calc.	*θ*_1_(C1-C2-N4-C5)/*θ*_2_(C2-N4-C5-C6)	*μ*_GS,TS_(D)
Gas phase (1.00)	–	–	–	–	NA (13.2)	NA (0.0/0.0)	NA (1.20)
Cyclohexane (2.02)	**101**, 138	147	9.5 ± 0.4	14.1	12.8 (12.8)	0.0/90.4 (−0.1/0.1)	3.85 (1.30)
Toluene (2.37)	80, **121**	145	12.4 ± 0.9	14.2	12.7 (12.9)	0.0/90.4 (−0.1/0.1)	3.97 (1.33)
CHCl_**3**_ (4.71)	71, **114**	135	16.9 ± 1.2	14.4	13.3 (13.7)	0.0/90.4 (−0.1/0.1)	4.44 (1.52)
MeOH (32.61)	**86**, 123	107	18.5 ± 1.4	14.4	14.4 (NA)	0.0/87.5 (NA)	5.25 (NA)
DMSO (46.83)	**82**, 121	124	23.3 ± 2.0	14.6	13.5 (NA)	0.0/90.2 (NA)	4.92 (NA)

Solvatochromic shifts of *λ*_max_ for the *Z* (Top) and *E* (Middle) isomers of ITI **1a**. Experimental *λ*_max,E_ values are obtained from TA that show two absorption maxima which are both reported and the maximum, of which the one with the highest absorption is highlighted in bold. Theoretical *λ*_max_ values and the difference of GS and ES dipole moments (Δμ_ES-GS_) were obtained at the SMD-TD-M06-2X/6-311++G(2df,2p) level using the SMD-M06-2X/6-31+G(d) geometries, from which also twisting angles *θ*_1_ and *θ*_2_ were derived (see Fig. 2a). Bottom: Thermal relaxation of ITI **1a**. Experimental half-lives were calculated from ms TA. The GS dipole moments for the transition state (*μ*_GS,TS_) were obtained at the SMD-M06-2X/6-31+G(d) level, at which also the twisting angles *θ*_1_ and *θ*_2_ as well as the activation barriers for thermal relaxation were derived (see Fig. 2a). The data in parentheses refer to a planar TS structure

The photo-isomerization of **1a** was followed by transient absorption spectroscopy (TA) in the millisecond time range, which revealed changes in the absorption spectrum upon irradiation at a short timescale. The transient spectra show a red-shifted absorption band, assigned to the thermally unstable *E*-isomer of the unsubstituted ITI **1a** (Fig. 1c, Supplementary Figs. 39–48) in the 500 to 600 nm region, where *Z*-ITI **1a** does not absorb. In all solvents, the spectrum of the *E*-isomer has two maxima (506–517 and at 549–554 nm), of which the most intense has been highlighted in bold (Table 1). ITI thus shows a large Δ*λ*_max_ between the two photo-isomers of over 100 nm. In comparison, HTIs usually show Δ*λ*_max_ of only 10 to 50 nm^22,39^.

The experimentally observed large Δ*λ*_max_ values are reproduced by the TD-DFT calculations, which further support the assignment of the absorption bands. Based on the Molecular Orbital (MO) analysis, the absorption band of the *E*-isomer corresponds to  the S_0_ → S_1_ transition with a predominant π→π* character and a small n→π* contribution (Table 1 and Supplementary Table 2). The huge bathochromic shift observed upon photoisomerization can be explained by the twist around the central double bond (C2 = N4) in the *E* isomer (see *θ*_1_ and *θ*_2_ in Fig. 2a). In the more twisted structure (*E*), the π orbital (HOMO) is destabilized (due to less efficient overlap of 2p orbitals of C2 and N4 atoms, see Fig. 2b) leading to a smaller energy gap in the *E* isomer.

The half-life for the *E* isomer of ITI **1a** in the thermal re-isomerization process was determined at room temperature to be in the millisecond time range, which is much shorter than found for HTI^22^. This finding can be ascribed to the presence of a nitrogen atom in ITI that can undergo inversion (Supplementary Fig. 30), a thermal relaxation mechanism also observed for azobenzenes^40^ and imine photoswitches^41^. The rate of nitrogen inversion is medium-dependent, with polar solvents increasing the reaction barrier^42^, which is consistent with our experimental data (Table 1, Supplementary Figs. 62, 63).

Theoretical observations of the thermal half-life are in line with the experimental ones, taking into account the limitations of continuum models to accurately describe the protic nature of MeOH. The calculations reveal that in all solvents the phenyl group is perpendicular to the molecular plane in the transition state for back isomerization from *E* to *Z*, although a concurrent (less stable) transition state with planar structure was identified in less polar solvents as well (Supplementary Note 4, Supplementary Table 5). The preference for the twisted structure is apparently related to the higher polarity of this conformation compared to the planar one (Supplementary Table 6, Supplementary Figs. 27–30) favoring its interactions with solvent molecules.

**Fig. 3:**
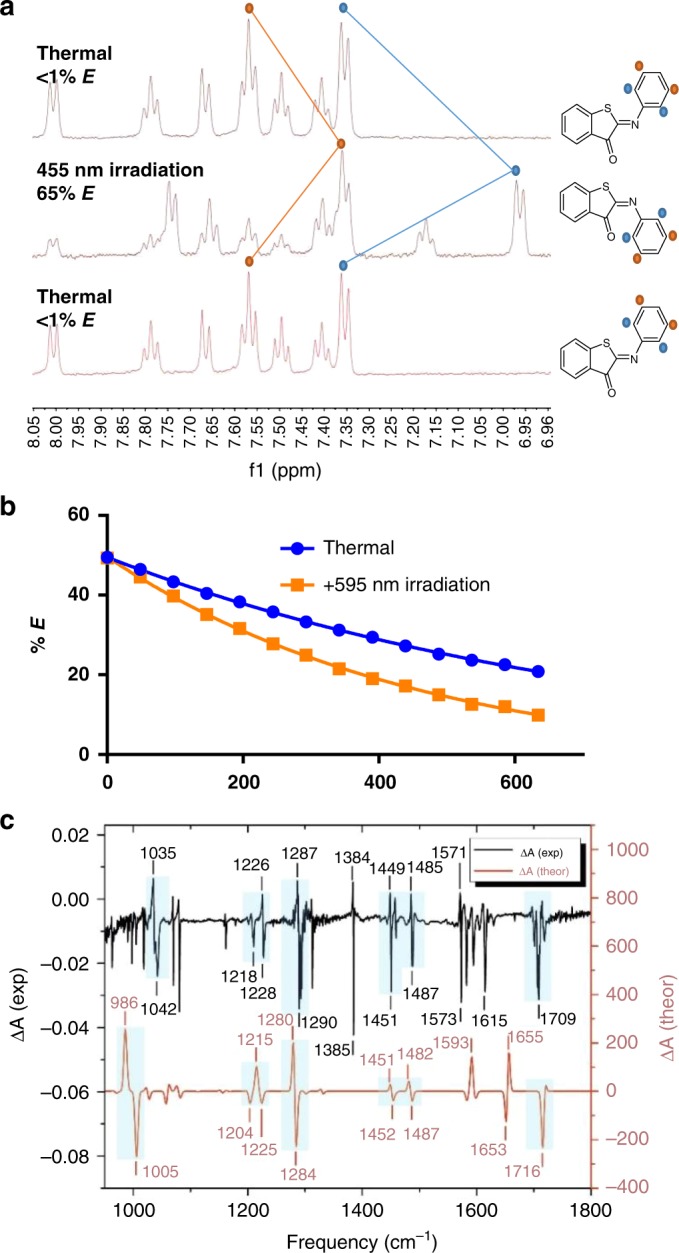
NMR and IR spectroscopy. **a** NMR spectra of ITI **1a** in CD_3_OD at −60 ^o^C for the thermally adapted, irradiated and again thermally adapted sample **b:** *E*-*Z* isomerization of ITI **1a** at −60 ^o^C in CD_3_OD, recorded without (thermal) and with *λ* = 595 nm irradiation (Supplementary Fig. 77, Supplementary Note 12) **c** *E*–*Z* FTIR difference spectrum recorded upon irradiation at 405 nm in KBr at  184 K for ITI **1a**. Comparison of experimental and theoretical IR difference spectra of **1a**. Experimental FTIR difference spectrum of the compound **1a** was obtained from the spectra in the dark and under 405 nm light measured at 184 K in a KBr pellet (Supplementary Figs. 84–86). Simulated difference spectrum was obtained from scaled harmonic GS IR spectra (scaling factor *f* = 0.98) of the *E*- and *Z*-isomers of **1a** in acetonitrile calculated with at the SMD-B3LYP/6-31 + + G(d,p) level. The experimental FTIR spectra are also reported in Supplementary Fig. 84 for better visualization. Further IR characterization can be found in Supplementary Notes 7,8, Supplementary Figs. 33–37 and Supplementary Table 11

The isomerization was further studied with low-temperature NMR experiments at −60 ^o^C. NMR spectra (Fig. 3a) showed that, upon irradiation with 455 nm light, the signals of the *Z*-isomer decreased with a concomitant rise of new signals that can be assigned to the *E*-isomer, reaching a photostationary state (PSS) of 65%. The upfield shift of proton signals upon photo-isomerization of **1a** is also predicted by calculations (see Supplementary Note 9 and Supplementary Table 12), further supporting our structure assignment. Thermal relaxation at −60 ^o^C resulted again in the formation of the *Z*-isomer with a half-life of 6.8 ± 0.5 min without any observable degradation. An Eyring analysis, based on the determination of the back-isomerization rate at different temperatures by NMR, allowed for the calculation of the thermodynamic properties of the *E-Z* re-isomerization step (Supplementary Figs. 78–82), showing Δ*H*^‡^ = 61.8 ± 5.2 kJmol^−1^ and Δ*S*^‡^ = 81.6 ± 23.4 JK^−1^mol^−1^, which results in a Δ*G*^‡^ = 86.1 ± 8.7 kJmol^−1^ (at 298 K).

An important feature of a photoswitch is the ability to be operated photochemically in both directions exclusively with visible light. To test whether the reverse *E-Z* isomerization can be achieved photochemically, ITI **1a** in CD_3_OD at −60 °C was switched to the *E*-isomer by irradiation with 455 nm (blue) light, and the rate of back-isomerization was then determined either without or with *λ* = 595 nm (orange) light irradiation. An approximately two-fold increase in the back-isomerization rate was observed under irradiation (Fig. 3b)^43^, showing that **1a** is indeed both a T- and P-type photoswitch, while the heating effect of irradiation could be excluded (Supplementary Fig. 77). Yet it must be noted that the observation of photochemical *E* to *Z* isomerization is not of additional value at room temperature, because of the fast thermal re-isomerization.

The less stable *E*-isomer was also further characterized by measuring *E-Z* difference FTIR spectra obtained upon irradiating the sample at *λ* = 405 nm at  184 K (Fig. 3c). Importantly, these spectra were acquired with the sample in a KBr pellet, demonstrating that isomerization also occurs at the solid state. The main spectral features related to structural differences between the two isomers are fairly well reproduced by the DFT calculations (see band assignment in Supplementary Table 11, Supplementary Figs. 35 and 36 and Supplementary Note 7.).

### *Z-E* isomerization of ITI is a fast process

**Fig. 4:**
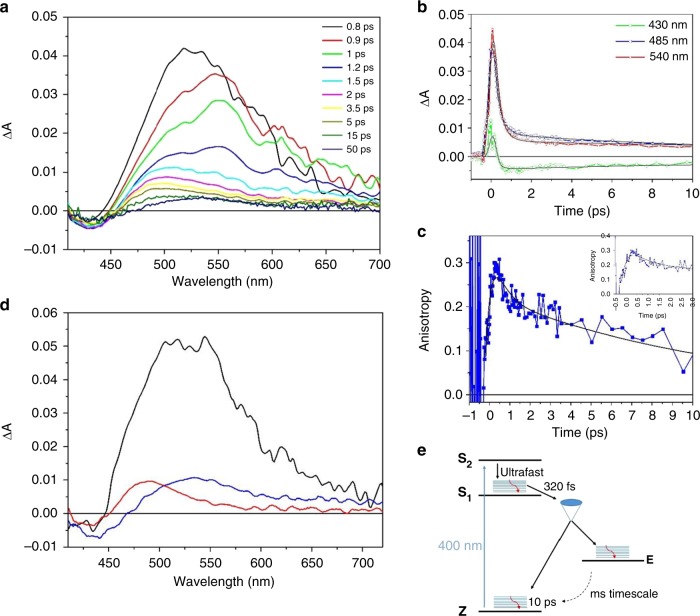
Ultra-fast Transient Spectroscopy of ITI. **a** Transient absorption spectra of unsubstituted ITI **1a** recorded in methanol with excitation at 400 nm. **b** Representative kinetic traces (open symbols) and fits obtained from target analysis (continuous line), **c** Time-resolved anisotropy, the initial 3 ps are shown in the inset, **d** Species-Associated Decay Spectra (SADS), obtained by analyzing the kinetic traces with the kinetic model depicted on the right-bottom side of the figure. The black curve represents the S_1_ state, the red curve hot Z isomer and the blue curve the E isomer. **e** Proposed model for photo-isomerization

Transient absorption measurements with sub-picosecond time resolution were performed to determine the timescale of forward *Z* to *E* isomerization of ITI, which is expected to be very fast, based on structural analogies with HTIs and azobenzenes^22,43^. For unsubstituted ITI **1a**, the spectra recorded immediately after excitation with *λ* = 400 nm light are dominated by a very broad excited state absorption band with an intensity that rapidly decays, leaving a constant weak differential signal as shown in the time-resolved spectra reported in Fig. 4a and the kinetic traces in Fig. 4b. Importantly, the long-living signal matches the one measured on the millisecond timescale (Fig. 1c), and can thus assigned unambiguously be as the *Z*-*E* difference spectrum. The very fast decay of the excited state absorption band indicates that isomerization itself is a very fast process, since the system has to reach the conical intersection (CI) leading to the formation of the *Z* and *E* isomers in their respective ground states before the deactivation of the excited states. In order to get additional kinetic information on the process, we measured the pump-probe anisotropy by recording the transient spectra with parallel and perpendicular polarization of the pump beam with respect to the probe. Interestingly, the resulting anisotropy signal, reported in Fig. 4c, shows a fast rise component, on a timescale of a few hundred femtoseconds, and a slower decay, occurring within 12–16 ps. The timescale of the anisotropy decay is in line with what has been observed for azobenzene in solution^44^. The rise of the anisotropy within the initial 500 fs indicates that a significant charge redistribution rapidly occurs once the molecule starts to move on the excited state potential energy surface towards the conical intersection region, in line with the computed large difference in transition dipole moments for the *Z* and *E* forms (Table 1). It is worth noticing that a similar rise in the anisotropy in a few hundred fs has been previously observed for rhodopsin, which is known to isomerize on an ultrafast timescale and interpreted in terms of rapid and substantial change in the charge distribution of the molecule due to the activation of the vibrational modes leading to isomerization^45^.

Our calculations indicate that the bright state of ITI is the S_2_ state. Taking into account the observed fast excited state decay, we therefore envisioned the excited state relaxation pathway to be similar to that of azobenzene. To extract the time constants describing the photodynamics of the system, we fitted the transient isotropic data with the kinetic scheme shown in Fig. 4e, retrieving the lifetimes reported therein and the Species-Associated Difference Spectra (SADS) of the transient intermediates (Fig. 4d). Upon excitation to S_2_, the system rapidly undergoes internal conversion towards S_1_, with a time constant below the time resolution of our measurements. This results in an unreasonable spectral shape for this state, which is not shown in Fig. 4d. The remaining SADS are assigned to the S_1_ state (black line), to the hot *Z* isomer (red curve) and the *E* isomer (blue curve). The very short S_2_ lifetime is again similar to what is known for azobenzene, for which a value of 50 fs has been recently determined^44,46^. The decay of the broad S_1_ excited state band within 320 fs and the rise of anisotropy on the same timescale indicate that ITI reaches the conical intersection region on a time scale competing with vibrational relaxation in S_1_. From there, the molecule relaxes to the ground state of either the *Z* and *E* isomers, where vibrational cooling takes place on a time scale of 10 ps.

Support to our hypothesis that isomerization starts from a hot S_1_ state comes from the computation of the forces acting on the individual atoms of ITI in S_2_ and S_1_ after vertical excitation, showing that the molecule undergoes more pronounced structural changes in the S_1_ state (for more details see Supplementary Fig. 26, Supplementary Note 3 and Supplementary Table 4). The presence of a nitrogen atom in the isomerizing double bond opens the possibility for isomerization to occur through either an inversion or rotation mechanism. The negligible change in the excited state relaxation time scale observed in solvents with different viscosity (see Supplementary Fig. 38) in first instance favors an inversion mechanism, although most probably the simple vision of motion along a single reaction coordinate is not realistic, as recently pointed out for azobenzene^44^.

### Substituent effects on ITI photo-isomerization

**Fig. 5:**
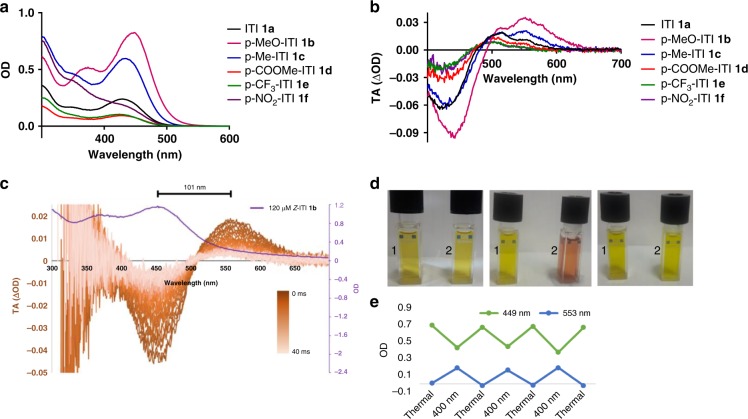
Spectroscopy studies on the substituent effects on ITI photo-isomerization. **a** Absorption spectra of 40 μM ITIs **1a**–**f** in MeOH. **b** Transient absorption spectra of ITIs **1a**–**f** in MeOH after irradiation at 430 nm after 3 ms delay. **c** Transient absorption spectroscopy of 120 μM *p*-MeO-ITI **1b** in aqueous PBS buffer (6.7% DMSO), irradiated with a 10 ns 430 nm light pulse and spectra recorded with 1 ms delay steps. The purple line indicates the absorption spectrum of 120 μM *Z*-ITI **1b** in aqueous PBS buffer (6.7% DMSO). The color bar represents increased delay in transient absorption spectroscopy. **d** Cuvettes 1 and 2 contain 200 µM ITI **1b** in MeOH. Left: both thermally adapted. Middle: cuvette 2 irradiated with 400 nm light while cooled at −60 °C in acetone bath. Right: reheating of cuvette 2 to room temperature. **e** Three cycles of photo-isomerisation of 100 µM **1b** in MeOH, thermally adapted and switched with 400 nm light, while cooled at −60 °C  in acetone bath (Supplementary Fig. 89)

The influence of the substituents on photoswitching of ITI was studied using a small library of ITIs with either an electron donating (**1b,c**) or an electron withdrawing group (**1d-f**). As shown in Fig. 5, electron donating groups (EDG) result in a slight red-shift of *λ*_max,Z_ and increased absorption, while electron withdrawing groups (EWG) result in a slight blue-shift of λ_max,Z_ and decreased absorption (Supplementary Fig. 64). Theoretical calculations reproduce this trend and show that the auxochromic effects are mainly due to the twist around the =N-C- central single bond (θ_2_, Fig. 2a). Indeed, θ_2_ is smaller for **1b,c**, leading to a more planar structure and favoring the electron delocalization (Supplementary Fig. 31 and Supplementary Table 8) upon excitation and increasing *λ*_max,Z_. In the ground state, EDGs increase the electron density on the phenyl ring which tends to “planarize” to increase conjugation with the thioindoxyl moiety in accordance with similar auxochromic affects have been observed in HTIs^47^.

Isomerization of the differently substituted ITIs was measured in MeOH upon irradiation with *λ* = 430 nm light (Fig. 5b, Supplementary Figs. 49–58). A new absorption band was found for all the substituted ITIs and for electron donating ITIs **1b** and **c** an impressively large Δ*λ*_max_ of over 100 nm was observed. ITI **1b** was dissolved in MeOH and irradiated with 400 nm while cooled to −60 ^o^C (Fig. 5d). Compared to the thermally adapted state, isomerization resulted in a clear change in color. Switching for several cycles of **1b** in MeOH did not result in observable degradation (Fig. 5e). For all ITIs, the quantum yield for forward switching was estimated to be between 4 and 6%, which is relatively low compared to many other photoswitches^21^. No clear correlation between Hammett parameter R and the quantum yield (Supplementary Note 13, Supplementary Table 14) for the single studied position was found, meaning that both electron withdrawing and electron donating groups are tolerated.

Our calculations show that the auxochromic effects on Δλ_max_ can be explained by a combination of geometrical and electronic effects (Supplementary Note 5). While *θ*_2_ is governing the auxochromic effects for the *Z* and *E* isomers in the same way (*θ*_2_ is larger for *E* than for *Z* but the extent to which *E* and *Z* are influenced by a substituent is similar), a twist around the C = N central double bond (*θ*_1_) is only observed for the *E* isomer. The *θ*_1_ twist, being more pronounced for EDG substituents (**1b**,**c**), leads to a stronger destabilization of the π orbital (HOMO) of the *E* isomer for these substituents compared to the *Z* isomer. Such geometrical feature partly contributes to the decrease of the Δ*λ*_max_ when going from **1b,c** to **1a,d,e,f**. In addition, the change of the dipole moment upon excitation for the *E* form decreases from 2.37 D (**1b**) to −5.85 D (**1f**) in methanol following the nature of the substituents (Table 2). We have found that the more negative Δμ, the larger destabilization of the ES with respect to GS. This electronic effect also contributes to a smaller Δ*λ*_max_ for EWG substituents (Supplementary Table 7, 8 and Supplementary Fig. 31).

**Table 2:** Computational studies on substituent effects on ITI photo-isomerization

		*Z*-isomer					
	*R* (Hammet constant *σ*)	*λ*_max,Z_ (nm)		Transition			
		exp.	calc.	ππ*/nπ*	*θ*_1_(C1-C2-N4-C5)/*θ*_2_(C2-N4-C5-C6)	Δμ_ES-GS,Z_ (D)	*ε* (mol^−1^cm^−1^)
**1a**	H (0.00)	429	398	S_0_→S_1_ 0.58/0.38	−179.8/54.1	1.55	4300
**1b**	MeO (−0.27)	448	413	S_0_→S_1_ 0.62/0.29	−179.1/38.6	4.43	11000
**1c**	Me (−0.17)	434	406	S_0_→S_1_ 0.61/0.32	−179.9/50.3	2.66	5700
**1d**	COOMe (0.45)	427	399	S_0_→S_1_ 0.56/−0.15	−179.1/60.8	0.85	2300
**1e**	CF_3_ (0.54)	424	391	S_0_→S_1_ 0.54/0.37	−179.1/62.0	−0.62	2100
**1** **f**	NO_2_ (0.78)	–	390	S_0_→S_1_ 0.53/−0.33	−178.9/67.3	−0.26	2600

		*E*-isomer				
	*R* (Hammet constant σ)	λ_max,E_ (nm)		Transition		
		exp.	calc.	ππ^*^/nπ^*^	*θ*_1_(C1-C2-N4-C5)/ *θ*_2_(C2-N4-C5-C6)	Δμ_ES-GS,E_ (D)
**1a**	H (0.00)	515, 552	505	S_0_→S_1_ 0.54/0.30	9.3/66.0	−4.16
**1b**	MeO (−0.27)	553	533	S_0_→S_1_ 0.58/−0.32	12.4/52.9	2.37
**1c**	Me (−0.17)	511, **548**,	519	S_0_→S_1_ 0.58/−0.31	9.9/63.2	−1.01
**1d**	COOMe (0.45)	503	484	S_0_→S_1_ 0.52/−0.31	0.26/93.0	−4.45
**1e**	CF_3_ (0.54)	500	482	S_0_→S_1_ 0.52/−0.31	2.23/86.1	−5.81
**1** **f**	NO_2_ (0.78)	501	470	S_0_→S_1_ 0.54/0.30	0.00/−92.9	−5.85

							Transition state		
	*R* (Hammet constant *σ*)	Δλ_max_ (nm)		*t*_1/2_ (ms)	*E*_a,E-Z_ (kcal/mol)		*α*(C2-N4-C5)/	Δ*μ*GS-TS,Z	
		exp.	calc.	exp.	exp.	calc.	*θ*_2_(C2-N4-C5-C6)	(D)	φ_Z-E_ (%)
**1a**	H (0.00)	**86**, 123	107	18.5 ± 1.4	14.4	14.4	177.2/87.5	−0.02	6.2
**1b**	MeO (−0.27)	105	120	12.7 ± 0.5	14.2	13.0	177.7/0.0	−2.45	4.5
**1c**	Me (−0.17)	77, **114**	113	21.1 ± 1.2	14.5	14.0	177.7/0.0	−2.68	5.4
**1d**	COOMe (0.45)	76	85	4.0 ± 0.3	13.6	13.1	177.6/92.0	0.38	6.3
**1e**	CF_3_ (0.54)	76	91	9.9 ± 1.0	14.1	13.6	177.6/90.1	0.94	4.9
**1** **f**	NO_2_ (0.78)	–	80	2.8 ± 0.5	13.4	12.0	177.8/90.1	2.48	4.1

Shifts of *λ*_max_ for the Z (Top) and E (Middle) isomers of ITIs **1a**–**f** in MeOH. Experimental *λ*_max,E_ values are obtained from TA that show two absorption maxima which are both reported with the maximum with the highest absorption highlighted in bold. Theoretical *λ*_max_ values and the difference of GS and ES dipole moments (Δμ_ES-GS_) were obtained at the SMD-TD-M06-2X/6-311++G(2df,2p) level using the SMD-M06-2X /6-31+G(d) geometries, from which also twisting angles (θ_1_ and θ_2_, Fig. 2a) were derived. Bottom: Thermal relaxation of ITIs **1a**–**f** in MeOH. Experimental half-lives were calculated from ms TA spectroscopy. The differences of dipole moment of the transition state and that of the Z-form in their GS (Δμ_GS-TS,Z_) were obtained at the SMD-M06-2X/6-31+G(d) level, at which also the transition state twisting angles (*θ*_1_ and *θ*_2_) as well as the activation barriers for thermal relaxation were derived

Apart from changes in the absorption spectra of *Z* and *E*, substituents also influence the rate of thermal relaxation of the *E* isomer (Table 2). No clear correlation between the Hammett parameter and the half-lives of the *E* isomer was observed, albeit the data suggested a trend in EWG groups results in faster re-isomerization (Supplementary Figs. 32, 65, 66, Supplementary Note 6, and Supplementary Table 9). The same correlation between Hammett parameter R and the half-lives of the *E* isomer was observed at −60 ^o^C upon 455 nm irradiation in the NMR experiment (Supplementary Notes 9 and 11, Supplementary table 13, Supplementary Figs. 67–76). DFT results were in line with these observations, revealing that the weak correlation of activation energy with the Hammett constants could be caused by qualitatively different relaxation paths for the EDG- and EWG-substituted (and neutral) ITIs. Whereas the *E*-*Z* relaxation proceeded through a planar TS structure in the case of **1b**-**c**, **1a,d-f** adopted a twisted conformation in the TS (Supplementary Fig. 31). The different behavior is a result of interplay between the stabilization of the TS due to π-electron delocalization (favoring the planar conformation) and the stabilization due to polarity of the TS (favoring the more polar twisted structure). By decreasing the electron density on the phenyl ring, EWG substituents enhance the interaction of the 2p orbital on nitrogen with π-orbitals of the phenyl ring favoring the twisted structure (Supplementary Figs. 27, 30 and 32).

### Isomerization of ITI in aqueous solutions

In the field of photopharmacology, photo-control over the stereochemistry of a double bond is used to establish a difference in biological activity between both photo-isomers, as has been demonstrated for azobenzene and hemithioindigo photoswitches^6,48^. For such biological applications of photoswitches, solubility at medicinally relevant conditions and photo-isomerization under aqueous and physiological conditions are crucial, yet are rarely observed for fully-visible-light switches. For example, photo-isomerization of HTI at physiological conditions has not been reported. To evaluate the performance of ITI in aqueous solutions, unsubstituted ITI **1a** was dissolved in phosphate buffered saline (PBS, pH 7.4, 1.7% DMSO) at ~30 μM. Irradiation with 400 nm light did not results in observable degradation (Supplementary Fig. 88). We also demonstrated that ITI has resistance against glutathione (GSH), which is found in concentrations up to 10 mM in cells and is the key factor for degradation of other molecular photoswitches^49^.

Isomerization of ITIs in aqueous PBS (pH 7.4, 6.7% DMSO) was studied using the most red-light shifted *p*-MeO-ITI **1b** (Fig. 5C, Supplementary Figs. 59, 60) with ms transient absorption spectroscopy. The *Z* isomer of **1b** has an absorption maximum at 459 nm. Upon irradiation with blue light, the *E* isomer was observed with an absorption maximum at 560 nm, demonstrating that a spectacular difference of absorption maxima is also maintained in aqueous solutions (Fig. 5c). From the same experiment, the half-life of the *E* isomer was found to be 10.0 ± 0.8 ms at room temperature.

## Discussion

For application in biological systems, new and improved switches are needed. This is underlined e.g. by a recent report by the group of Thorn-Seshold^48^, in which the first HTI-based photo-controlled pharmacophore was reported. This study demonstrates both the potential of indigoid-based photoswitches as well as the need for improved band separation of photo-isomers and improved water solubility.

Here we described the discovery of Iminothioindoxyls, a class of small, synthetically accessible visible-light photoswitches with excellent photochemical properties, showing very fast switching and an absorption band separation of photo-isomers of over 100 nm. Importantly, ITIs switch in solid state and in solvents ranging in polarity from cyclohexane to water, being therefore suitable for a very wide range of applications, varying from responsive materials to photopharmacology.

ITIs show unique properties when compared to other fully-visible-light-responsive photoswitches. A promising feature of ITIs is the millisecond half-life, making them useful for applications requiring fast responses. Indeed, many biological processes, such as signal transduction and neuronal communication, operate at the millisecond scale and their photomodulation has been achieved with quickly re-isomerizing switches^50,51^. Furthermore, ITIs forward switching is faster and shows better band separation than hemithioindigo, while also operating on a completely different mechanism for thermal relaxation. Finally, photo-isomerization of HTI in aqueous solutions at physiological pH has so far not been realized, while for ITI it could be readily observed. Also if compared to red-shifted azobenzenes, ITIs present favorable properties: they are slightly smaller in structure and synthetically more accessible, showing faster switching and a larger absorption band separation between the two isomers, high stability under irradiation and under heavily reducing conditions such as those encountered in living cells.

Currently, the fast re-isomerization of ITIs prevents the use of their bi-directional photochemical isomerization at room temperature. To fully exploit the various properties of this class of photoswitches, an increased build-up and a longer lifetime of the *E* isomer is needed. This could be achieved through judiciously substitution patterns that improve the quantum yield and increasing the thermal barrier of re-isomerization. Similar situations have occurred in the past when other types of switches have been developed. In view of the successful studies that have followed to optimize these switches, we are confident that also for ITIs this will be a realistic target. We therefore consider the discovery of ITIs a break-through in the field of photocontrol, providing the starting point for developing improved photoswitches, resulting in major opportunities towards responsive systems well beyond those offered by the current very limited repertoire of all-visible light switches.

## Methods

### Organic synthesis

All reported starting materials, chemical reagents and organic solvents in this study were bought from Sigma–Aldrich, Acros, Fluka, Fischer, TCI and were used as received. Dry DCM was purified by passage through an MBraun SPS-800 solvent purification column. All aqueous solutions were prepared using deionized water. Kieselgel 60, F_254_ silica gel plates (Merck, TLC silica gel 60 F_254_) were used for TLC (Thin Layer Chromatography) analysis and UV light of 254 nm and potassium permanganate solution (KMnO_4_) were used for the detection of compounds. Drying of solutions was performed using dry MgSO_4_ and solvents and other volatiles were removed using a rotary evaporator.

### Analytical procedures

Nuclear Magnetic Resonance (NMR) spectra were recorded using an Agilent Technologies 400-MR (400/54 Premium Shielded) spectrometer (400 MHz), at room temperature (22–24 °C), unless indicated otherwise. The multiplicities of the signals are reported as follows: s (singlet), d (doublet), t (triplet), q (quartet) or m (multiplet). All ^13^C-NMR spectra are ^1^H-broadband decoupled. Melting points (Mp) were measured using a Stuart analogue capillary melting point SMP11 apparatus. High-resolution mass spectrometric (HRMS) measurements were performed using a Thermo scientific LTQ OrbitrapXL spectrometer, which is equipped with ESI ionization. In the experimental procedures, the mass of the molecule-ion [M + H]^+^ are reported in m/z-units. Absorption spectra were measured using an Agilent 8453 UV/Vis diode array. All solutions for absorption spectra were prepared in Uvasol® grade solvents and were measured in quartz cuvettes with a 1 cm path-length. Purity was determined using LCMS, for which the following setup was used: Column**:** ACQUITY UPLC**®** HSS T3 1.8 µm, 2.1 × 150 mm; Detection**:** Total Ion Count (TIC), *λ*_1_ = 254 nm, *λ*_2_ = 430 nm; Flow**:** 0.3 mL/min; Eluent A**:** 0.1% formic acid in HPLC grade demineralized H_2_O; Eluent B**:** 0.1% formic acid in acetonitrile; Gradient Program: (0–1 min) 5% eluent B; (1–8 min) linear gradient to 90% eluent B; (8–11 min) 90% eluent B; (11–12 min) linear gradient to 5% eluent B; (12–17 min) 5% eluent B.

### Computational studies

The ground state (GS) structures of the *Z*/*E*-isomers and the GS transition state (TS) of the backward reaction (*E*→*Z*; thermal relaxation process) for ITIs (**1a-f**) were optimized at the M06–2X level^36^ using the 6–31+G(d) atomic basis set^37^, since this exchange-correlation functional is known to perform well not only for the GS thermochemistry, but also in describing excited states^52^. In addition, ITIs are not subject to the known TD-DFT limitations such as charge-transfer (see Supplementary Table 6), double excitations (see t1 and t2 amplitudes), singlet-triplet transition, etc. All minima were checked against the presence of imaginary frequencies. The TS structures were obtained by geometry optimization starting from a structure with the angle C2-N4-C5 set to very close to 180°. This choice was based on the potential energy scan for the out-of-plane distortion from the in-plane-TS structure showing that the distortion is energetically unfavourable (Supplementary Fig. 28). The optimized TS structures (first-order saddle points) were checked against the presence of a single imaginary frequency. The optimized GS structures are presented in Supplementary Note 1 and Supplementary Fig. 22. The solvent effects were considered employing the solvation Model based on Density (SMD)^38^. Cyclohexane (CHX), toluene (TOL), chloroform (CHL), methanol and dimethylsufoxide (DMSO) are used consistently with experimental data. The IR spectra were simulated at the B3LYP/6–31 + + G(d,p) level^53,54^ which was found to provide a reasonable agreement with the experimental FTIR spectra for the Z-isomers (Supplementary Note 7, Supplementary Figs. 33–36). The IR band assignment was based on the potential energy distribution (PED) analysis^55^ by using the VEDA 4 program^56^. Vertical excitation energies (VEE) were obtained with a larger basis set, namely 6–311 + + G(2df,2p). SMD was combined with the corrected linear response (cLR) approach^57^ to model VEE within the non-equilibrium regime. (TD)-DFT calculations were performed using the Gaussian09 and Gaussian16 programs^58,59^. All Gaussian default thresholds and algorithms were used except for improving optimization. In the latter case, a threshold of 10^–5^ a.u. on average residual forces was imposed, a self-consistent field convergence criterion of 10^−10^ a.u., and the use of the ultrafine DFT integration grid. Gas phase CC2 and ADC(2) calculations of the excitation energies were performed using aug-cc-pVTZ basis set with the Turbomole program [TURBOMOLE V6.6 2014, a development of University of Karlsruhe and Forschungszentrum Karlsruhe GmbH, 198–2007, TURBOMOLE GmbH, since 2007; available from http://www.turbomole.com]. NMR shieldings for the protons in Z/E-isomers of **1a** were obtained with the Gauge-Independent Atomic Orbital (GIAO) method^60^ with the B3LYP functional and the 6–31 + + G(d,p) basis set. Chloroform environment was treated using the SMD model. Proton shielding for TMS selected as reference was computed under the same conditions using the M06–2X /6–31+G(d) geometry.

### Ultrafast spectroscopy

Ultrafast transient absorption spectra of unsubstituted Iminothioindoxyl **1a** were measured on a system consisting of a Ti:sapphire laser oscillator (Spectra Physics Tsunami) and regenerative amplifier system (BMI Alpha 1000) which produced pulses of 100 femtosecond at 800 nm with an average output power of 450 to 500 mW. Excitation pulses at a wavelength of 400 nm were obtained by second harmonic generation of the fundamental laser output in a 2 mm thick BBO crystal. For all measurements in methanol, the pump beam polarization was set either to perpendicular or parallel with respect to the parallel probe beam by rotating a λ/2 plate. For measurement in solvents other than methanol, polarization was set to magic angle so as to exclude rotational contributions to the transient signal. From the parallel and perpendicular intensities the anisotropy r(t) is calculated using Eq. (1).

1 $$r(t) = \frac{{I_\parallel - I_ \bot }}{{I_\parallel + 2I_ \bot }}$$

where $I_\parallel$and $I_ \bot$ are the signal intensity respectively recorded with parallel and perpendicular pump polarization. The isotropic signal in methanol is obtained from the parallel and perpendicular signals using Eq. (2).

2 $$I_{Iso} = \frac{{I_\parallel + 2I_ \bot }}{3}$$

The excitation powers were on the order of 50 to 100 nJ. The probe pulses were generated upon focusing the 800 nm radiation beam partially on a 2 mm thick sapphire window, after which it was passed through the sample. Subsequently the white light probe was sent to a flat field monochromator which was coupled to a home-made CCD detector (http://lens.unifi.it/ew). Transient spectra were recorded in a time interval spanning up to 500 ps. All measurements were performed in a quartz cell (2 mm thick) mounted on a movable stage in order to refresh the solution and avoid undesired photochemical degradation of the sample.

Analysis of the Transient data was performed using Singular Value Decomposition (SVD)^61^ and global analysis^62^, which allows the simultaneous fit at all the measured wavelengths with a combination of exponential decay functions. The kinetic scheme employed for data analysis, involving fast internal conversion among two close-lying excited states and excited state decay associated to partial *Z*-*E* isomerization, is shown in Fig. 4 of the main text. Data analysis has been performed using the software GLOTARAN^63^.

### Nanosecond transient absorption spectroscopy

Nanosecond transient absorptions were recorded with an in-house assembled setup. For all ITIs and all solvents, an excitation wavelength of 430 nm was used. The excitation wavelength of 430 nm was generated using a tunable Nd:YAG-laser system (NT342B, Ekspla) comprising the pump laser (NL300) with harmonics generators (SHG, THG) producing 355 nm to pump an optical parametric oscillator (OPO) with SHG connected in a single device. The laser system was operated at a repetition rate of 5 Hz. The probe light running at 10 Hz was generated by a high-stability short arc xenon flash lamp (FX-1160, Excelitas Technologies) using a modified PS302 controller (EG&G). Using a 50/50 beam splitter, the probe light was split equally into a signal beam and a reference beam with and focused on the entrance slit of a spectrograph (SpectraPro-150, Princeton Instruments). The probe beam (*A* = 1 mm^2^) was passed through the sample cell and orthogonally overlapped with the excitation beam on a 1 mm × 1 cm area. The excitation energy was recorded by measuring the excitation power at the back of an empty sample holder. In order to correct for fluctuations in the flash lamp spectral intensity, the reference was used to normalize the signal. Both beams were recorded simultaneously using a gated intensified CCD camera (PI-MAX3, Princeton Instruments) which has an adjustable gate of minimal 2.9 ns. A delay generator (DG535, Stanford Research Systems, Inc.) was used to time the excitation pulse, the flash lamp, and the gate of the camera. The setup was controlled by an in-house written LabView program.

### In situ NMR irradiation experiments

NMR spectra were recorded with an Agilent Technologies Inova 500 Spectrometer (500 MHz), and for in situ irradiation, a set-up based on LED and an option fiber were used, according to a reported system^64^. The fiber-optic cable (M28L05; Ø400 μm, 0.39 NA, SMA-SMA Fiber Patch Cable, 5m) and the LEDs were purchased from Thorlabs: royal blue 455 nm Fiber-coupled LED(M455F1, 11.0 mW); amber 595 nm Fiber-coupled LED(M595F2, 11.0 mW). NMR tubes were purchased from Wilmad-LabGlass (SP Scienceware):WGS-5BL, Coaxial Insert for 5 mm NMR Sample Tube and 535-PP-7, 5 mm Thin Wall Precision NMR Sample Tube 7” L, 600 MHz.

### FTIR

Low-temperature FTIR spectra were recorded on a FTIR Bruker IFS 120 HR spectrometer with maximum resolution 0.002 cm^−1^. For current measurements spectra were registered with 1 cm^−1^ spectral resolution. The instrument is equipped with a globar IR source and a MCT detector. The sample has been cooled using a liquid helium cold tip closed cycle cryostat (minimal nominal temperature 5 K), temperature has been monitored at the sample position using a K-type thermocouple (reading error 0.1 K)^65^.

The in situ irradiation source was a 80 mW laser diode, with a spot size of 6 × 4 mm, centered at 405 nm (FWHM ~10 nm). The sample was prepared as a KBr pellet, and contained in a home-made cell equipped with two calcium fluoride windows. Spectra without and under irradiation were measured at 184 K.

## Supplementary information

Supplementary Information

## Data Availability

The authors declare that the data supporting the findings of this study are available within the paper and its supplementary information files. Additional data on methods used are available from the corresponding author upon reasonable request.
