# Supplementary material for: Iminothioindoxyl as a molecular photoswitch with 100 nm band separation in the visible range
Source: Nat Commun. 2019 Jun 3;10:2390. doi: 10.1038/s41467-019-10251-8 (PMC6546742; doi:10.1038/s41467-019-10251-8)
Supplement: Supplementary file 1 — Supplementary Information [file 41467_2019_10251_MOESM1_ESM.pdf]

Supporting information to:

Iminothioindoxyl as a molecular photoswitch with 100 nm band separation in the visible range

Hoorens MWH *et al*.,

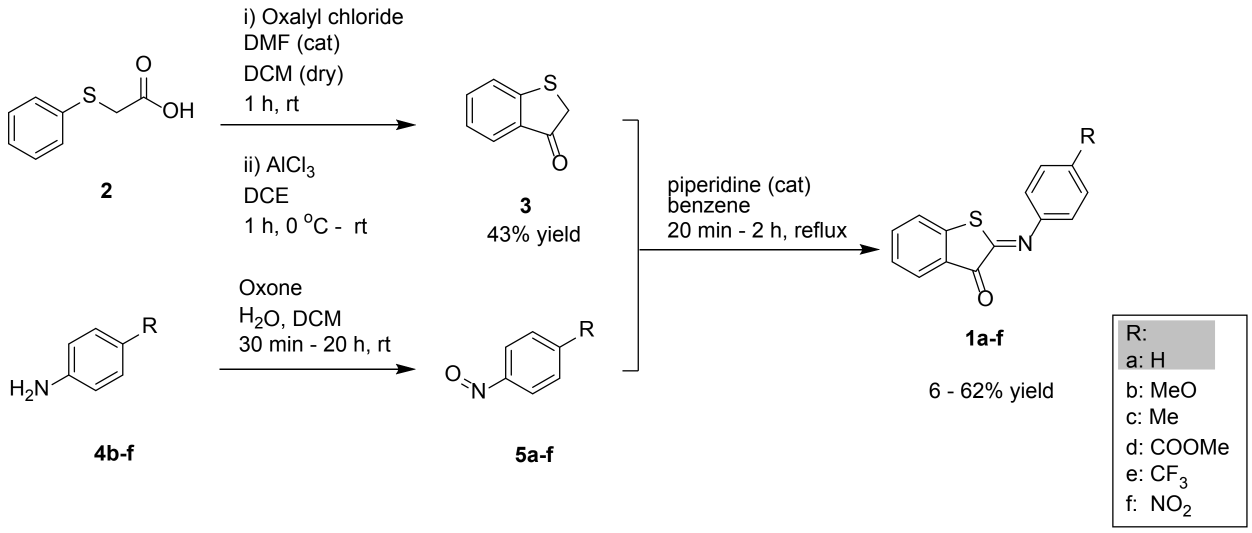

Supplementary Figure 1: *Synthesis of ITIs **1a-f**.*

Supplementary Methods:

Benzo[b]thiophen-3(2H)-one 3

2-(Phenylthio)acetic acid **2** (2.06 g, 12.3 mmol) was dissolved in DCM (dry, 5 mL). Oxalylchloride (1.7 mL, 2.5 g, 20 mmol) and DMF (1 drop) were added. The reaction mixture was stirred at room temperature for 1 h. Gas formation was observed during the reaction and after no gas formation was observed any more, the reaction mixture was concentrated *in vacuo* to remove all solvents and remaining oxalylchloride. The crude reaction mixture was dissolved in DCE (10 mL) and cooled to 0 °C. $AlCl_3$ (2.6 g, 20 mmol) was added portion-wise. The reaction mixture was allowed to reach rt and was stirred further for 1 h. The reaction was stopped when the reaction mixture formed large solid and subsequently the reaction mixture was diluted with ice water (100 mL) and was extracted with DCM (3 x 20 mL). The combined organic layers were washed with water (50 mL), dried with $MgSO_4$ and concentrated *in vacuo*. The product was purified by flash chromatography (Silicagel 40 – 63 nm, 100% Et_2O) and the product was obtained as a red solid (0.80 g, 5.4 mmol, 43% yield). Mp: 46 – 48 °C, lit: 62 – 64 °C,[1] ^{1}H NMR (400 MHz, $CDCl_3$) δ 3.79 (s, 2H, C**H**$_2$), 7.21 (t, *J* = 8.2 Hz, 1H, Ar**H**), 7.43 (d, *J* =

8.5 Hz, 1H, Ar**H**), 7.55 (t, *J* = 7.8 Hz, 1H, Ar**H**), 7.78 (d, *J* = 7.8 Hz, 1H, Ar**H**). The ^{1}H NMR spectrum corresponds to literature.[1]

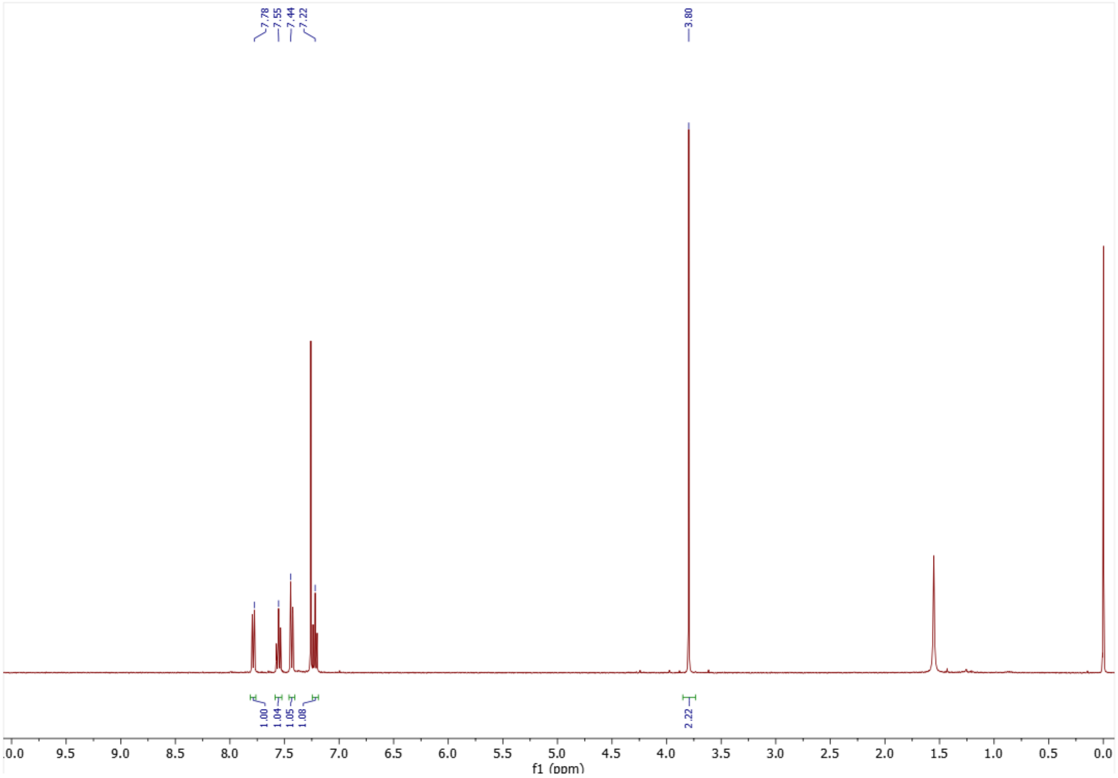

***Supplementary Figure 2:** ^{1}H NMR spectrum of compound **3** in $CDCl_3$*

1-Methoxy-4-nitrosobenzene 5b

4-Methoxyaniline **4b** (1.10 g, 8.94 mmol) was dissolved in DCM (20 mL) and H_2O (100 mL) and Oxone (5.41 g, 17.6 mmol) were added. The reaction mixture was stirred vigorously at room temperature for 30 min. After completion, 1N HCl (50 mL) was were added and the crude reaction mixture was extracted with DCM (3 x 50 mL). The combined organic layers were washed with 1N HCl (50 mL), water (50 mL) and brine (50 mL), dried with $MgSO_4$ and concentrated *in vacuo*. The crude product was flushed over a plug of silica gel (Silicagel 40 – 63 nm) in pentane and concentrated *in vacuo*. The crude product was obtained as a yellow solid and directly used without further purification.

1-Methyl-4-nitrosobenzene 5c

p-Toluidine **4c** (1.04 g, 9.75 mmol) was dissolved in DCM (40 mL) and H_2O (30 mL) and Oxone (5.92 g, 19.3 mmol) were added. The reaction mixture was stirred vigorously at room temperature for 55 min. After completion, 1N HCl (50 mL) was added and the crude reaction mixture was extracted with DCM (3 x 50 mL). The combined organic layers were washed with 1N HCl (50 mL), water (50 mL) and brine (50 mL), dried with $MgSO_4$ and concentrated *in vacuo*. The crude product was flushed over a plug of silica gel (Silicagel 40 – 63 nm) in pentane and concentrated *in vacuo*. The crude product was obtained as a yellow to green solid and directly used without further purification.

Methyl 4-nitrosobenzoate 5d

Methyl-4-aminobenzoate **4d** (2.00 g, 13.2 mmol) was dissolved in DCM (20 mL). To the solution, Oxone (8.17 g, 26.5 mmol) in water (80 mL) was added. The reaction was stirred at room temperature for 1 hour. After completion, DCM (100 mL) and water (100 mL) were added. and the aqueous phase was extracted with DCM (2 x 50 mL). The combined organic layers were washed with 1N HCl (1 x 50 mL), sat. aq. $NaHCO_3$ (2 x 50 mL), water (50 mL) and brine (50 mL). The organic phase was dried with $MgSO_4$ and concentrated *in vacuo*. The product was obtained as a yellow solid (1.12 g, 6.79 mmol, 52% yield) and directly used without further purification.

1-Nitroso-4-(trifluoromethyl)benzene 5e

4-(trifluoromethyl)aniline **4e** (1.01 g, 6.2 mmol) was dissolved in DCM (60 mL). To the solution, Oxone (3.91 g, 6.26 mmol) in water (60 mL) was added. The reaction was stirred at room temperature for 35 minutes. After completion, 1N HCl (50 mL) were added and the crude reaction mixture was extracted with DCM (3 x 50 mL). The combined organic layers were washed with 1N HCl (50 mL), water (50 mL) and brine (50 mL), dried with $MgSO_4$ and

concentrated *in vacuo*. The product was obtained as a yellow solid and directly used without further purification.

1-nitro-4-nitrosobenzene 5f

4-Nitroaniline **4f** (1.04 g, 7.53 mmol) was dissolved in DCM (40 mL) and H_2O (30 mL) and Oxone (4.75 g, 15.3 mmol) were added. The reaction mixture was stirred vigorously at room temperature for 20 h. After completion, 1N HCl (50 mL) was added and the crude reaction mixture was extracted with DCM (3 x 50 mL). The combined organic layers were washed with 1N HCl (50 mL), water (50 mL) and brine (50 mL), dried with $MgSO_4$ and concentrated *in vacuo*. The crude product was flushed over a plug of silica gel (Silicagel 40 – 63 nm) in pentane and concentrated *in vacuo*. The crude product was yielded was a yellow solid (yield not determined) and directly used without further purification.

(*Z*)-2-(phenylimino)benzo[b]thiophen-3(2H)-one 1a (ITI)

Benzo[b]thiophen-3(2H)-one **3** (100 mg, 0.66 mmol) was dissolved in benzene (5 mL). Nitrosobenzene (0.11 g, 1.0 mmol) and 1 drop of piperidine were added. The reaction mixture was stirred for 3 h at reflux. After completion, DCM (50 mL) and water (50 mL) were added and the mixture extracted with DCM (3 x 20 mL). The combined organic layers were washed with 1N HCl (50 mL), sat. aq. $NaHCO_3$ (50 mL) and brine (50 mL), dried with $MgSO_4$ and concentrated *in vacuo*. The product was purified by flash chromatography (Silicagel 40 – 63 nm, toluene). The compound was obtained as an orange solid (109 mg, 0.46 mmol, 69% yield). Mp: 132 – 134 °C. NMR (400 MHz, $CDCl_3$) δ 7.25 – 7.35 (m, 4H, Ar**H**), 7.39 (d, *J* = 7.8 Hz, 1H, Ar**H**), 7.45 (t, *J* = 7.6 Hz, 2H, Ar**H**), 7.61 (t, *J* = 7.6 Hz, 1H, Ar**H**), 7.95 (d, *J* = 7.6 Hz, 1H, Ar**H**). ^{13}C NMR (101 MHz, $CDCl_3$) δ 121.0, 124.9, 126.7, 137.3, 127.8, 129.3, 137.0, 144.5, 149.4, 156.4, 185.4, HRMS (ESI+) calc. for. [$M+H^+$] ($C_{14}H_{10}NOS^+$) Exact Mass: 240.0478, found: 240.0481. The NMR spectra correspond to literature.[2]

(*Z*)-2-((4-methoxyphenyl)imino)benzo[b]thiophen-3(2H)-one 1b (*p*-MeO-ITI)

Benzo[b]thiophen-3(2H)-one **3** (100 mg, 0.66 mmol) was dissolved in benzene (5 mL). Crude 1-methoxy-4-nitrosobenzene **5b** and 1 drop of piperidine was added. The reaction mixture was stirred for 75 minutes at reflux. After completion, DCM (50 mL) and water (50 mL) were added and the crude reaction mixture was extracted with DCM (3 x 20 mL). The combined organic layers were washed with 1N HCl (50 mL), sat. aq. $NaHCO_3$ (50 mL) and brine (50 mL), dried with $MgSO_4$ and concentrated *in vacuo*. The product was purified by flash chromatography (Silicagel 40 – 63 nm, pentane/Et_2O 1:1). The compound was obtained as a red solid (46 mg, 0.17 mmol, 26% yield). Mp: 129 – 130 °C, ^{1}H NMR (400 MHz, $CDCl_3$) δ 3.86 (s, 3H, C**H**$_3$), 7.00 (d, *J* = 9.0 Hz, 2H, Ar**H**), 7.33 (t, *J* = 7.5 Hz, 1H, Ar**H**), 7.41 (m, 3H, Ar**H**), 7.63 – 7.58 (t, *J* = 7.6 Hz, 1H, Ar**H**), 7.95 (d, *J* = 7.7 Hz, 1H, Ar**H**). ^{13}C NMR (101 MHz, $CDCl_3$) δ 55.5, 114.6, 124.6, 124.8, 126.6, 127.7, 127.8, 136.6, 141.3, 144.5, 152.5, 159.6, 185.7. HRMS (ESI+) calc. for. [M+H$^+$] ($C_{15}H_{12}NO_2S^+$) Exact Mass: 270.0583, found: 270.0583. The NMR spectra correspond to literature.[2]

(*Z*)-2-(*p*-tolylimino)benzo[b]thiophen-3(2H)-one 1c (*p*-Me-ITI)

Benzo[b]thiophen-3(2H)-one **3** (100 mg, 0.66 mmol) was dissolved in benzene (5 mL). Crude 1-methyl-4-nitrosobenzene **5c** and 1 drop of piperidine was added. The reaction mixture was stirred for 20 minutes at reflux. After completion, DCM (50 mL) and water (50 mL) were added and the crude reaction mixture was extracted with DCM (3 x 20 mL). The combined organic layers were washed with 1N HCl (50 mL), sat. aq. $NaHCO_3$ (50 mL) and brine (50 mL), dried with $MgSO_4$ was concentrated *in vacuo*. The product was purified by flash chromatography (Silicagel 40 – 63 nm, toluene). The compound was obtained as a yellow to light brown solid (100 mg, 0.39 mmol, 59% yield). Mp: 139 – 141 °C. NMR (400 MHz, $CDCl_3$) δ 2.39 (s, 3H, C**H**$_3$), 7.25 (m, 4H, Ar**H**), 7.33 (t, *J* = 7.8 Hz, 1H, Ar**H**), 7.40 (d, *J* = 7.9 Hz, 1H, Ar**H**), 7.60 (t, *J* = 7.6 Hz, 1H, Ar**H**), 7.95 (d, *J* = 7.7 Hz, 1H, Ar**H**). ^{13}C NMR (101 MHz, $CDCl_3$) δ 21.2, 121.6,

124.9, 126.6, 127.7, 127.8, 129.9, 136.8, 137.8, 144.6, 146.5, 155.0, 185.6. HRMS (ESI+) calc. for. [M+H$^+$] ($C_{15}H_{12}NOS^+$) Exact Mass: 254.0634, found: 254.0638

Methyl (*Z*)-4-((3-oxobenzo[b]thiophen-2(3H)-ylidene)amino)benzoate 1d (*p*-COOMe-ITI)

Benzo[b]thiophen-3(2H)-one **3** (100 mg, 0.66 mmol) was dissolved in benzene (5 mL). Crude methyl 4-nitrosobenzoate **5d** (0.17 g, 1.1 mmol) and 1 drop of piperidine were added. The reaction mixture was stirred for 80 minutes at reflux. After completion, DCM (50 mL) and water (50 mL) were added and the crude reaction mixture was extracted with DCM (3 x 20 mL). The combined organic layers were washed with 1N HCl (50 mL), sat. aq. $NaHCO_3$ (50 mL) and brine (50 mL), dried with $MgSO_4$ and concentrated *in vacuo*. The product was purified by flash chromatography (Silicagel 40 – 63 nm, toluene). The compound was obtained as an orange solid (0.14 g, 0.47 mmol, 71% yield). Mp: 162 – 164 °C. NMR ^{1}H (400 MHz, $CDCl_3$) δ 3.92 (s, 3H, COOC**H**$_3$), 7.21 (d, *J* = 8.6 Hz, 2H, Ar**H**), 7.35 (t, *J* = 7.6 Hz, 1H, Ar**H**), 7.38 (d, *J* = 7.9 Hz, 1H, Ar**H**), 7.62 (t, *J* = 7.6 Hz, 1H, Ar**H**), 7.93 (d, *J* = 7.7 Hz, 1H, Ar**H**), 8.11 (d, *J* = 8.6 Hz, 2H, Ar**H**) ^{13}C NMR (101 MHz, $CDCl_3$) δ 52.2, 120.1, 125.0, 127.0, 127.6, 127.9, 128.4, 131.0, 137.3, 143.9, 153.7, 158.4, 166.4, 185.0, HRMS (ESI+) calc. for. [M+H$^+$] ($C_{16}H_{12}NO_3S^+$) Exact Mass: 298.0532, found: 298.0538

(*Z*)-2-((4-(trifluoromethyl)phenyl)imino)benzo[b]thiophen-3(2H)-one 1e (*p*-CF_3-ITI)

Benzo[b]thiophen-3(2H)-one **3** (100 mg, 0.66 mmol) was dissolved in benzene (5 mL). Crude methyl 4-nitrosobenzoate **5e** and 1 drop of piperidine were added. The reaction mixture was stirred for 1 h at reflux. After completion, DCM (50 mL) and water (50 mL) were added and the crude reaction mixture was extracted with DCM (3 x 20 mL). The combined organic layers were washed with 1N HCl (50 mL), sat. aq. $NaHCO_3$ (50 mL) and brine (50 mL), dried with $MgSO_4$ and concentrated *in vacuo*. The product was purified by flash chromatography (Silicagel 40 – 63 nm, pentane/Et_2O 3:1). The compound was obtained as a yellow solid (0.018

g, 0.06 mmol, 6% yield). Mp: 114 – 116 °C. ^{1}H NMR (400 MHz, $CDCl_3$) δ 7.28 (d, *J* = 8.2 Hz, 2H, Ar**H**), 7.35 – 7.42 (m, 2H, Ar**H**), 7.65 (t, *J* = 8.0 Hz, 1H, ArH), 7.71 (d, *J* = 8.3 Hz, 2H, Ar**H**), 7.97 (d, *J* = 6.9 Hz, 1H, Ar**H**). ^{13}C NMR (101 MHz, $CDCl_3$) δ 120.5, 125.1, 126.6 (q, *J* = 3.6 Hz), 127.0, 127.6, 128.0, 128.8 (q, *J* = 32 Hz) 137.33, 143.7, 152.8, 158.9, 185.0. HRMS (ESI+) calc. for. [M+H$^+$] ($C_{15}H_9NOSF_3^+$) Exact Mass: 308.0352, found: 308.0356

(*Z*)-2-((4-nitrophenyl)imino)benzo[b]thiophen-3(2H)-one 1f (*p*-NO_2-ITI)

Benzo[b]thiophen-3(2H)-one **3** (100 mg, 0.66 mmol) was dissolved in benzene (5 mL). Crude 1-nitro-4-nitrosobenzene **5f** and 1 drop of piperidine were added. The reaction mixture was stirred for 1 h at reflux. After completion, DCM (50 mL) and water (50 mL) were added and the crude reaction mixture was extracted with DCM (3 x 20 mL). The combined organic layers were washed with 1N HCl (50 mL), sat. aq. $NaHCO_3$ (50 mL) and brine (50 mL), dried with $MgSO_4$ and concentrated *in vacuo*. The product was purified by flash chromatography (Silicagel 40 – 63 nm, toluene). The compound was obtained as a yellow solid (0.064 g, 0.22 mmol, 33% yield). Mp: 178 – 180 °C. ^{1}H NMR (400 MHz, $CDCl_3$) δ 7.26 (d, *J* = 8.7 Hz, 2H, Ar**H**), 7.39 (m, 2H, Ar**H**), 7.66 (t, *J* = 7.6 Hz, 1H, Ar**H**), 7.96 (d, *J* = 7.6 Hz, Ar**H**), 8.32 (d, *J* = 8.8 Hz, 2H, Ar**H**). ^{13}C NMR (101 MHz, $CDCl_3$) δ 120.5, 125.2, 125.3, 127.3, 127.4, 128.1, 137.6, 143.2, 145.9, 155.4, 159.9, 184.6. HRMS (ESI+) calc. for. [M+H$^+$] ($C_{14}H_9N_2O_3S^+$) Exact Mass: 285.0328, found: 285.0333

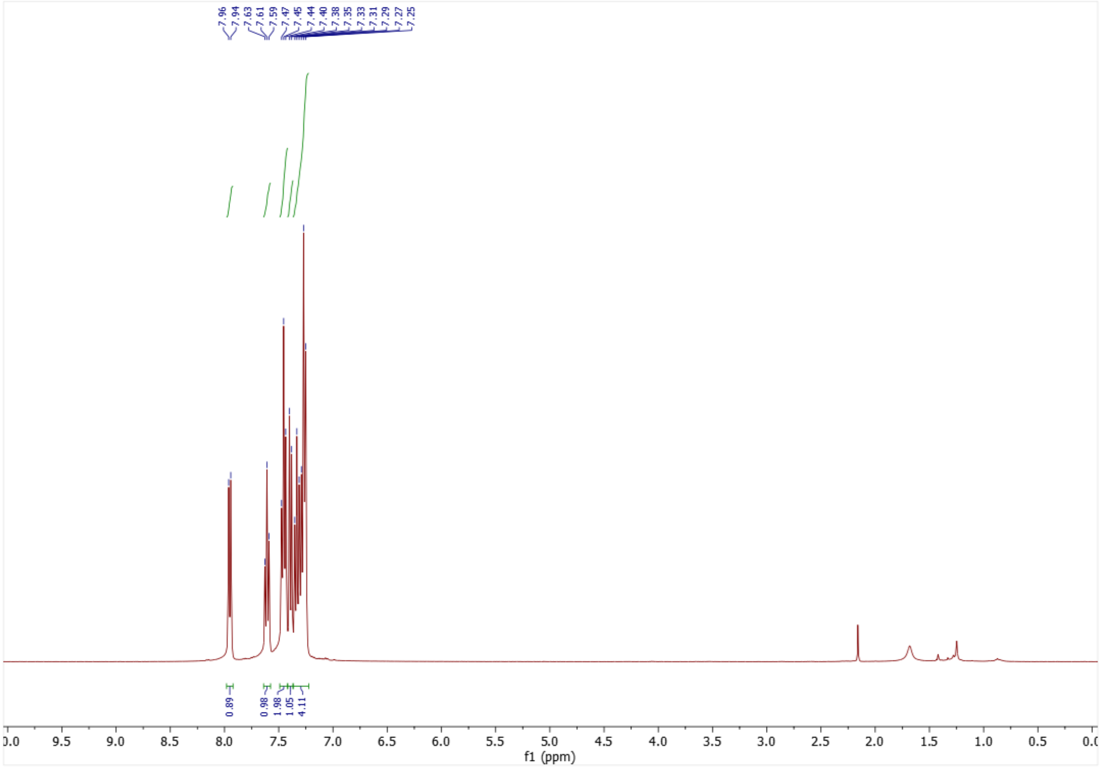

Supplementary Figure 3: *^{1}H NMR spectrum of **1a** in $CDCl_3$*

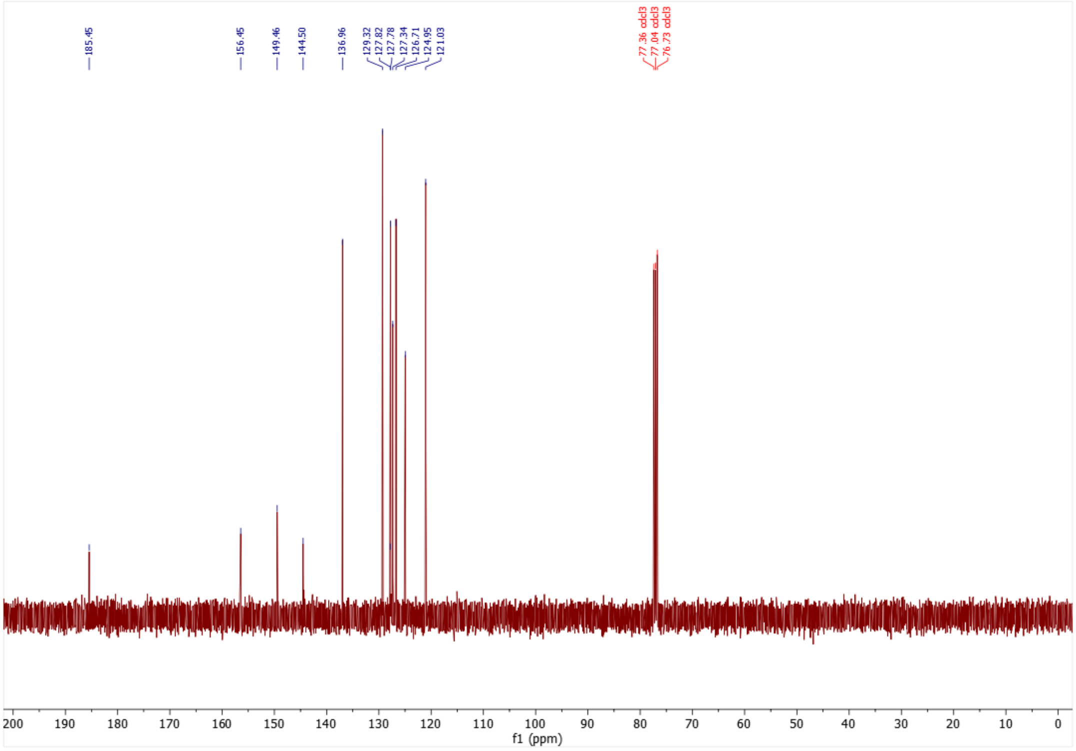

Supplementary Figure 4: *^{13}C NMR spectrum of **1a** in $CDCl_3$*

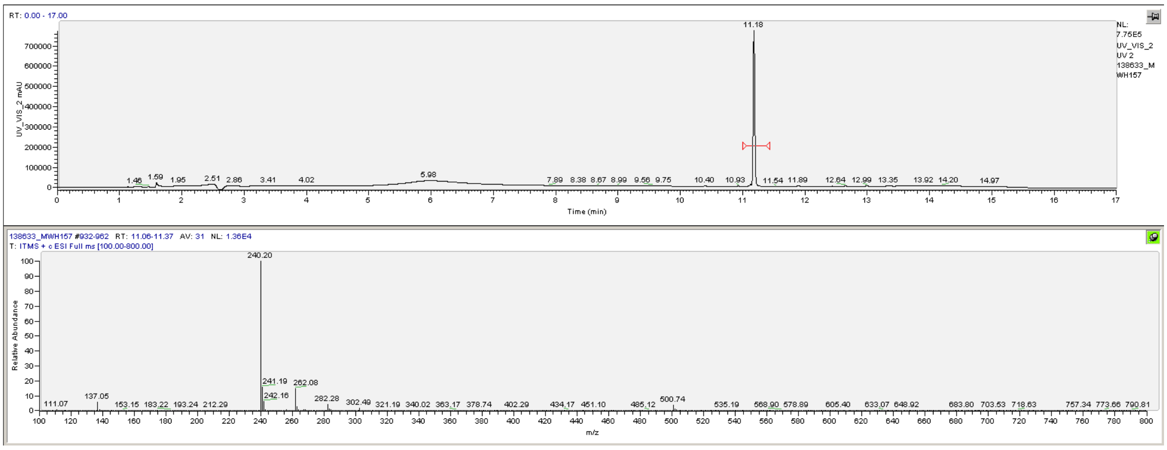

Supplementary Figure 5: *LC trace of* ***1a****, bottom: mass spectrum at selected retention time.*

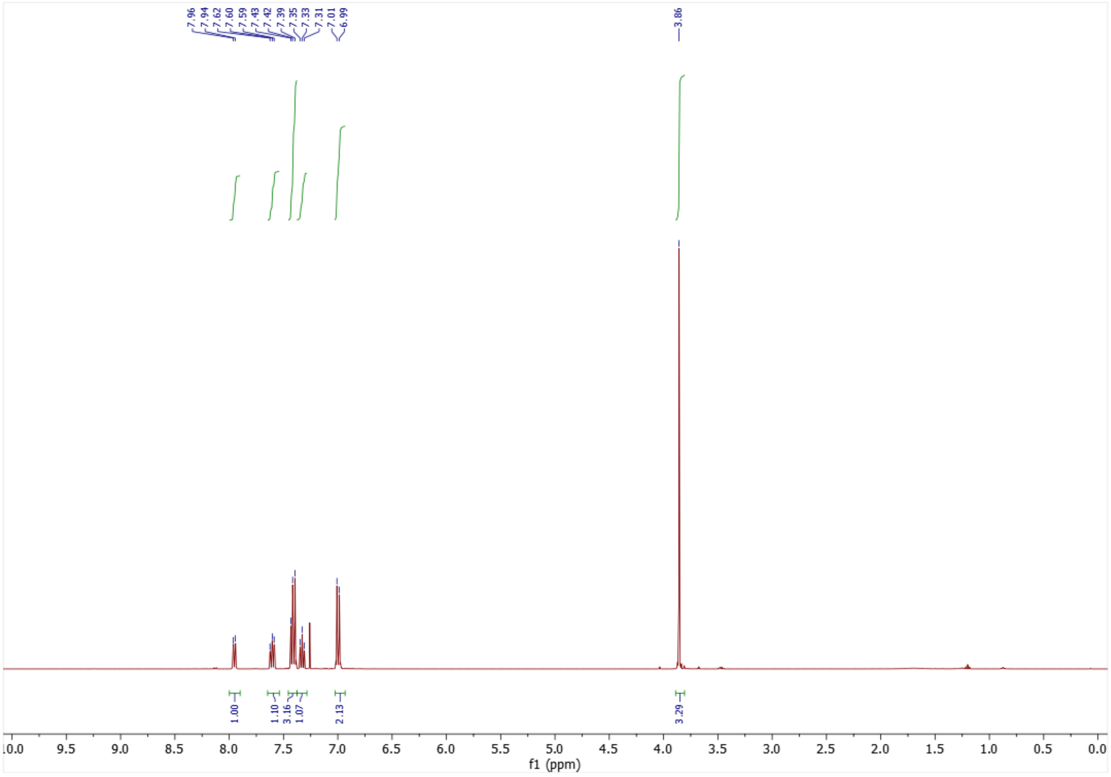

Supplementary Figure 6: *^{1}H NMR spectrum of* ***1b*** *in* $CDCl_3$

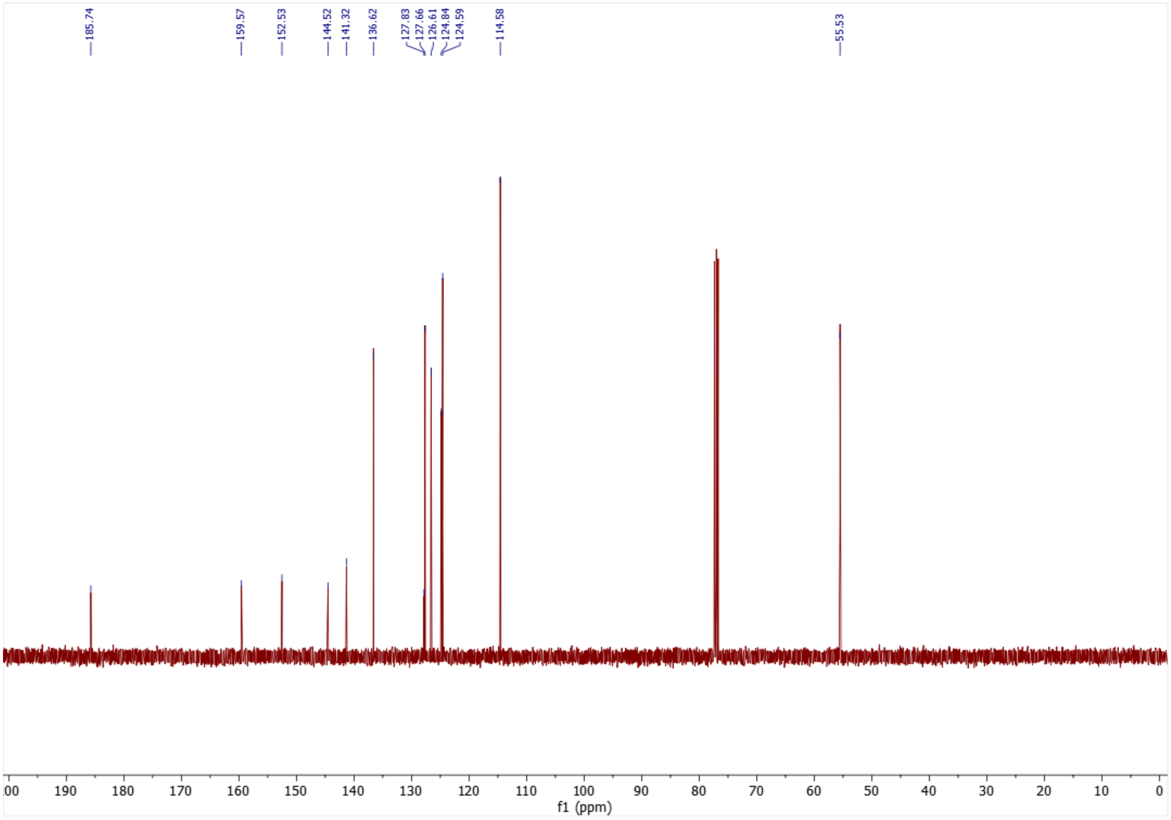

Supplementary Figure 7: _^{13}C NMR spectrum of **1b** in CDCl₃_

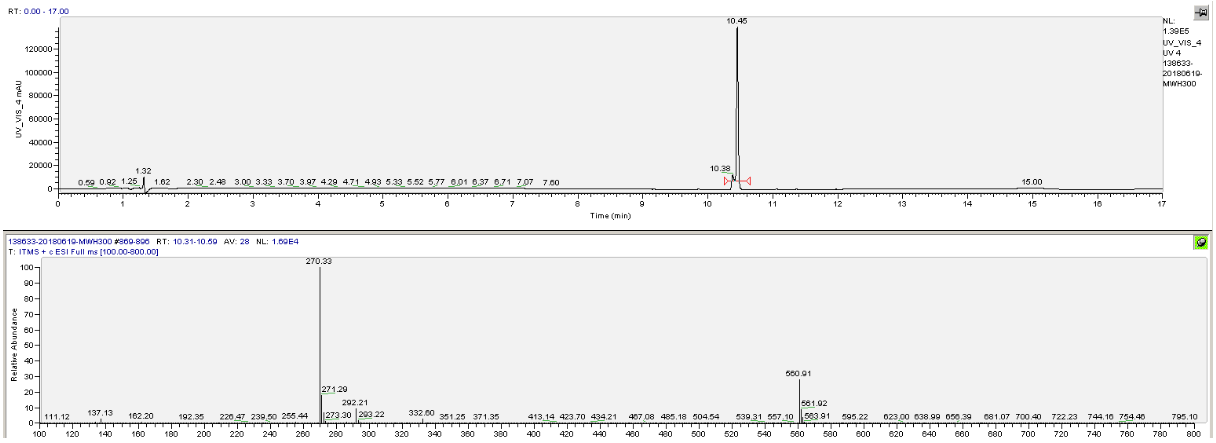

Supplementary Figure 8: _LC trace of **5b**, bottom: mass spectrum at selected retention time._

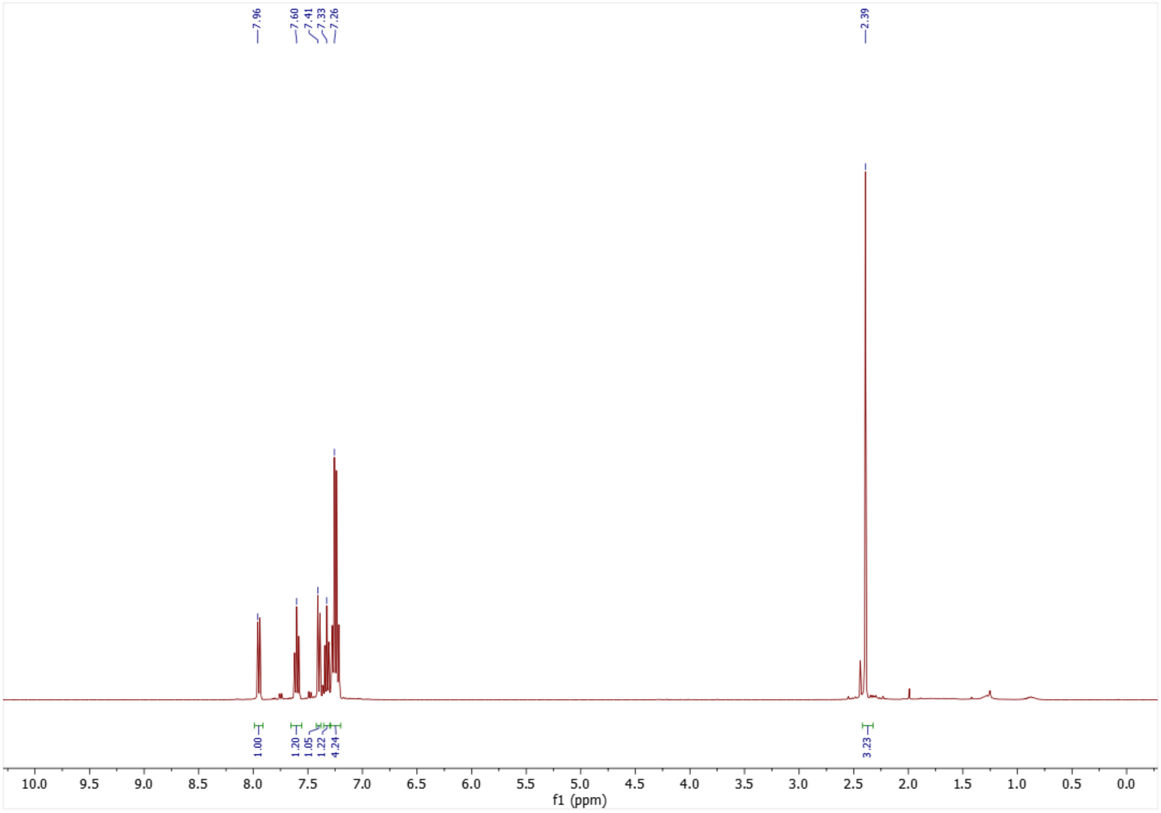

Supplementary Figure 9: *^{1}H NMR spectrum of* ***1c*** *in $CDCl_3$*

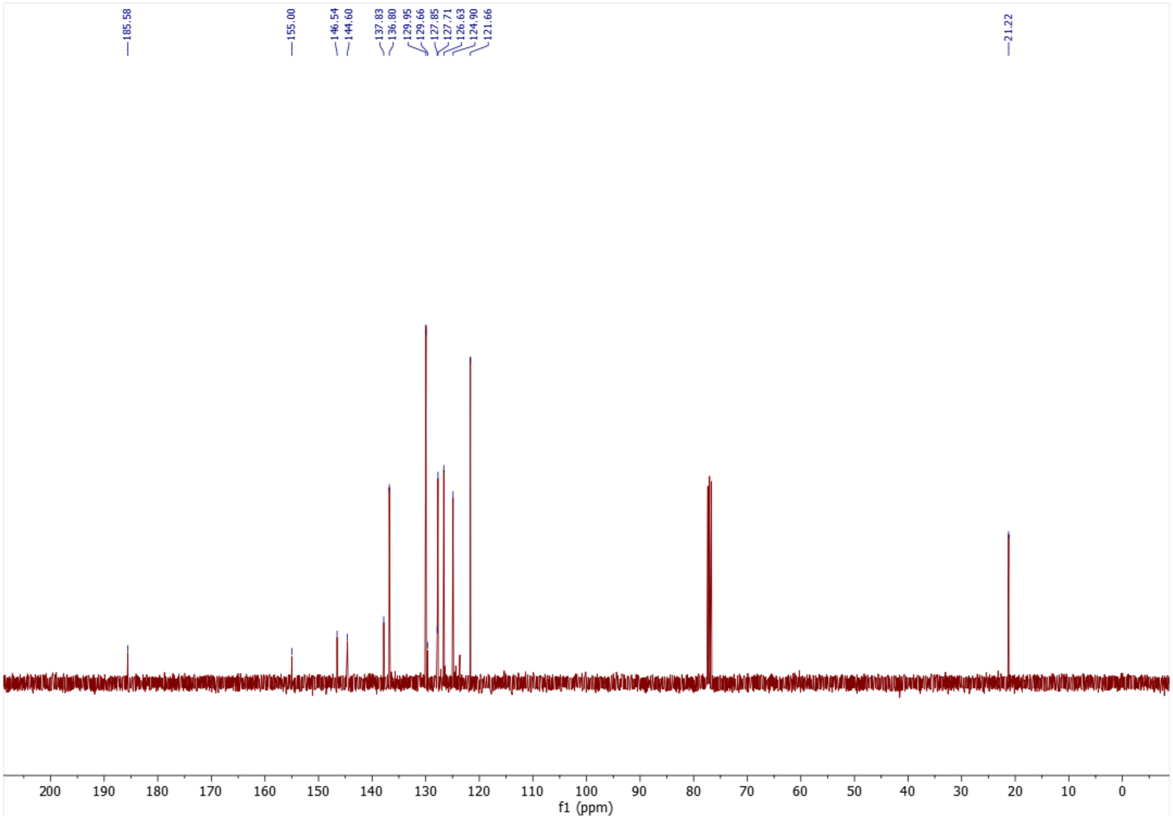

Supplementary Figure 10: *^{13}C NMR spectrum of* ***1c*** *in $CDCl_3$*

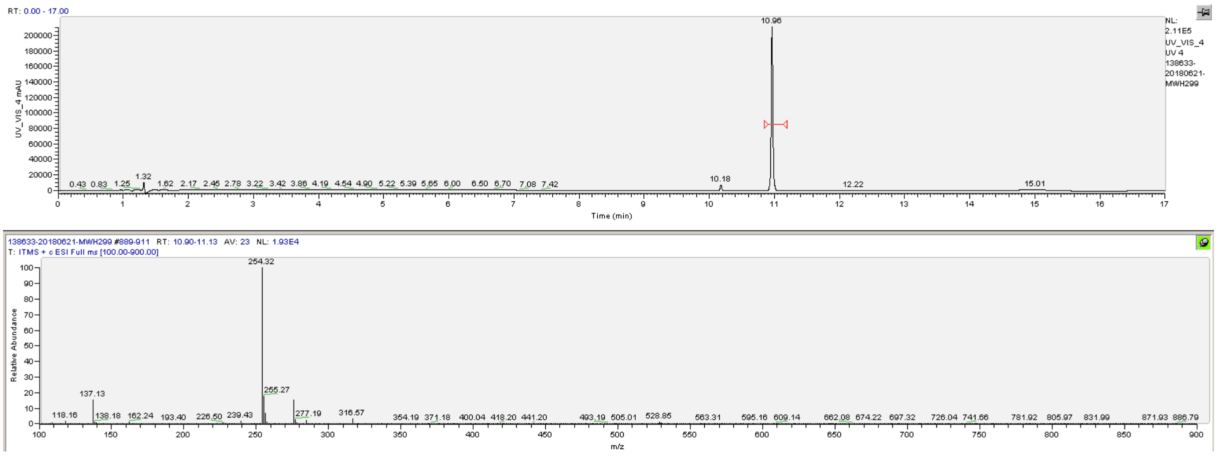

Supplementary Figure 11: *LC trace of **1c**, bottom: mass spectrum at selected retention time.*

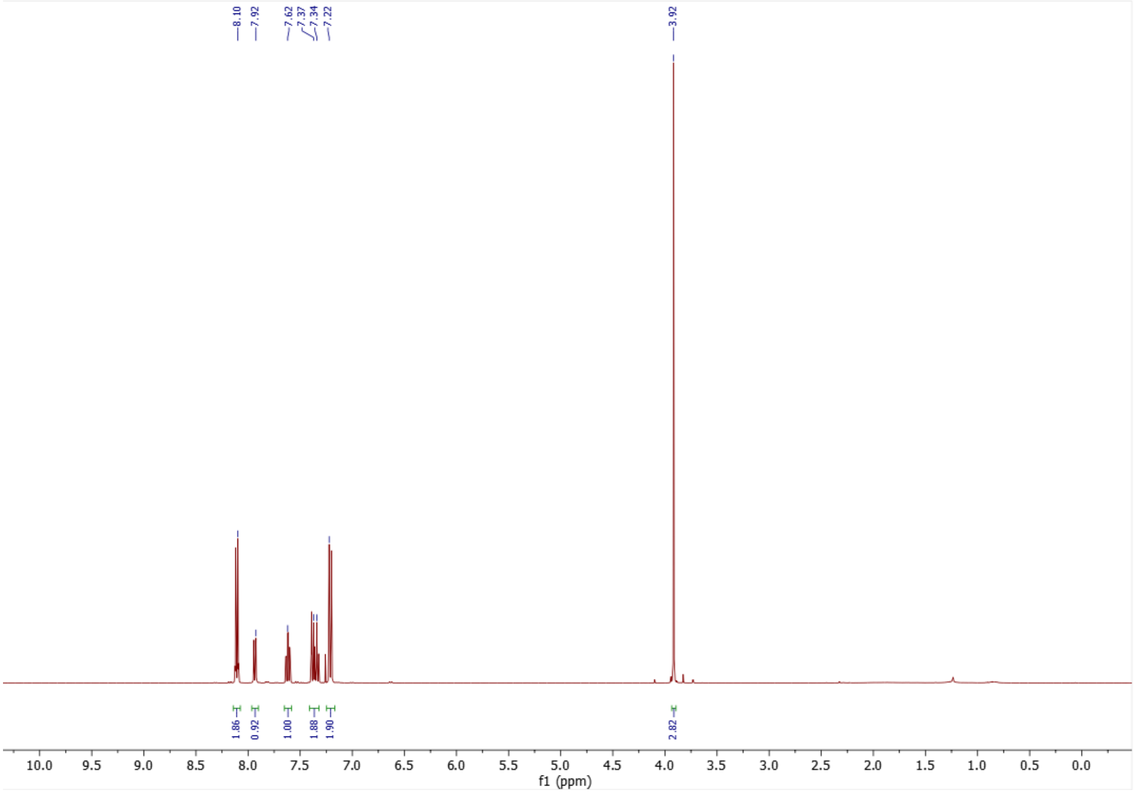

Supplementary Figure 12: *^{1}H NMR spectrum of **1d** in $CDCl_3$*

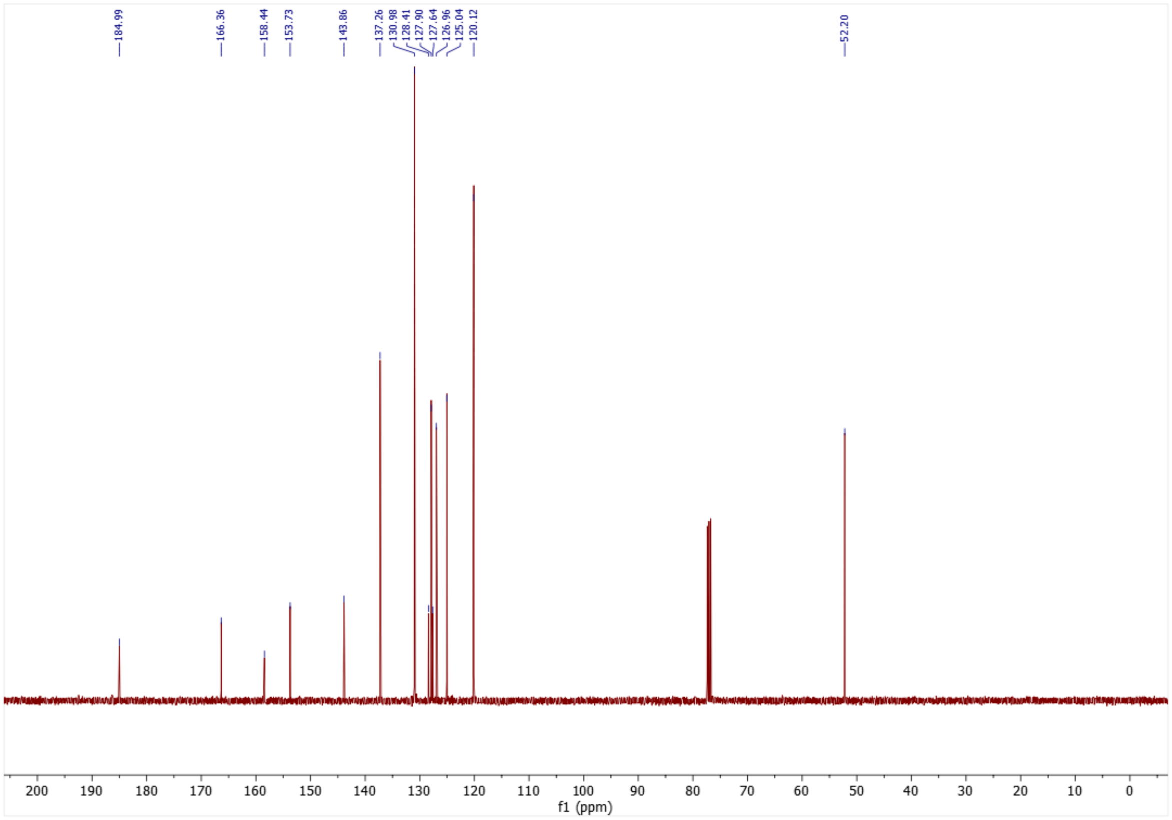

Supplementary Figure 13: *^{13}C NMR spectrum of **1d** in $CDCl_3$*

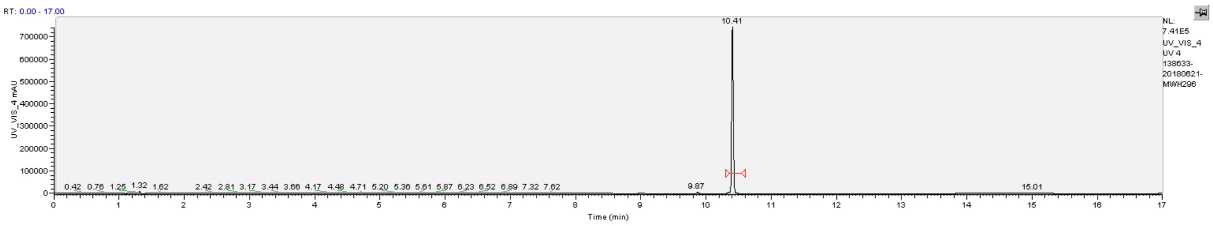

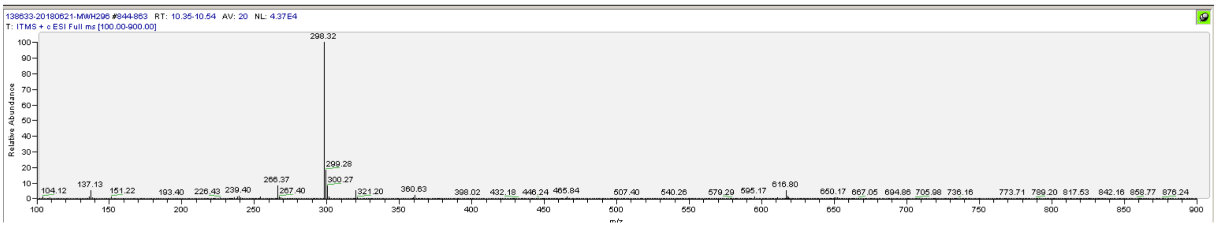

Supplementary Figure 14: *LC trace of **5d**, bottom: mass spectrum at selected retention time.*

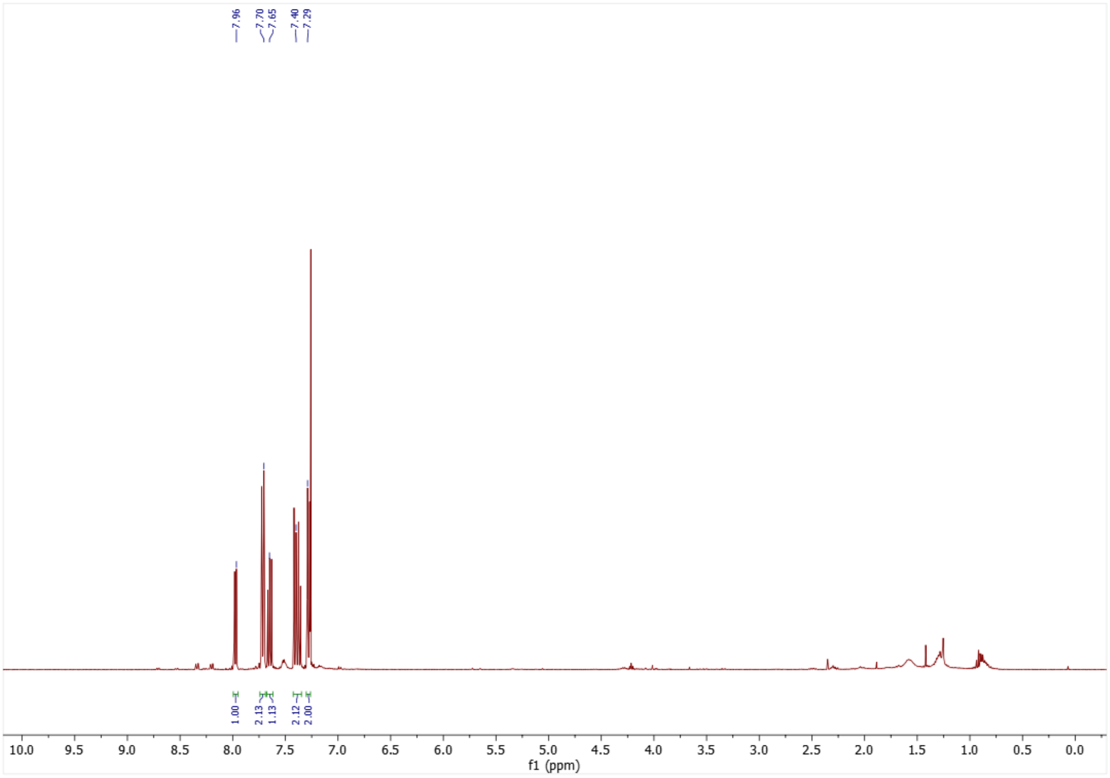

Supplementary Figure 15: *^{1}H NMR spectrum of **1e** in $CDCl_3$*

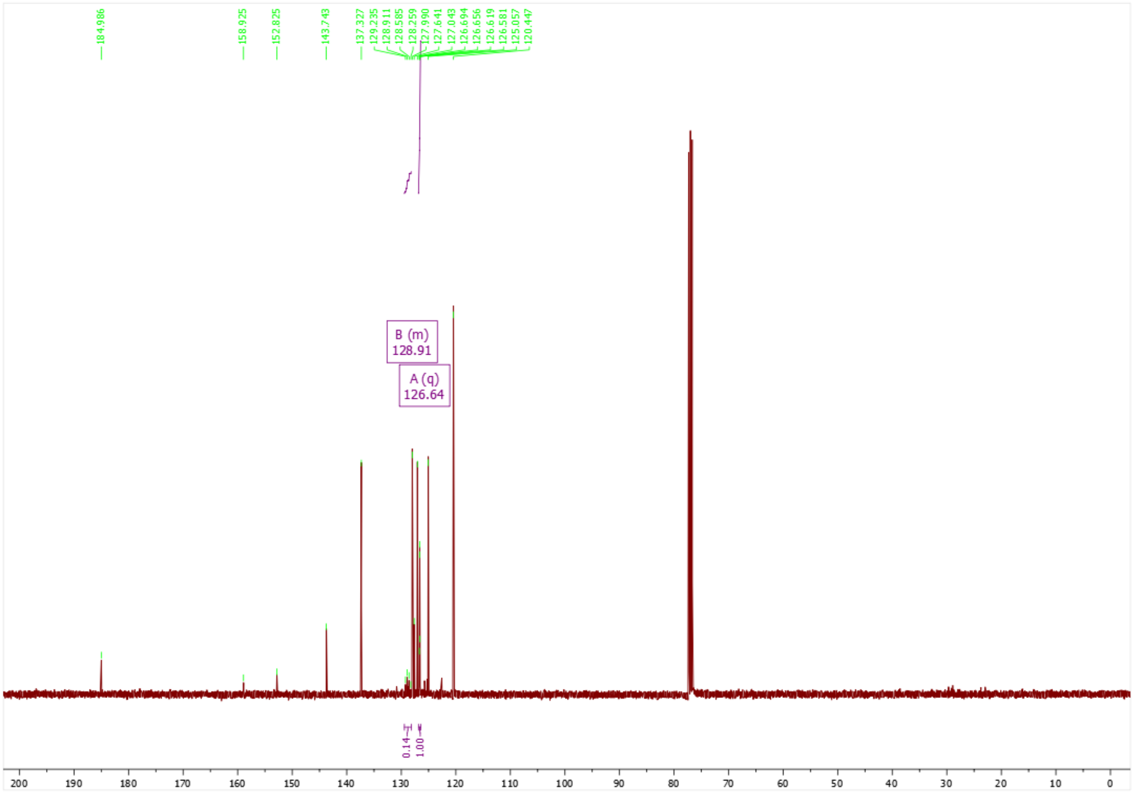

Supplementary Figure 16: *^{13}C NMR spectrum of **1e** in $CDCl_3$*

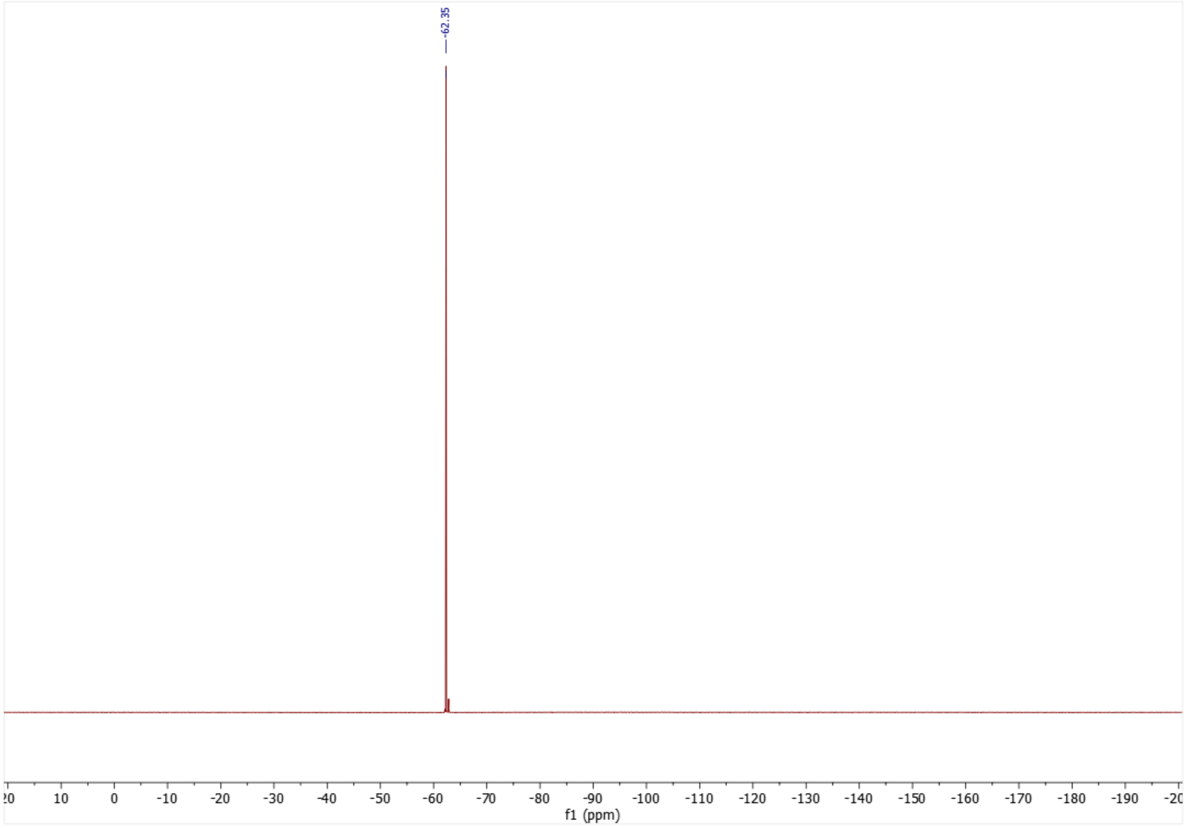

Supplementary Figure 17: *^{19}F NMR spectrum of **1e** in $CDCl_3$*

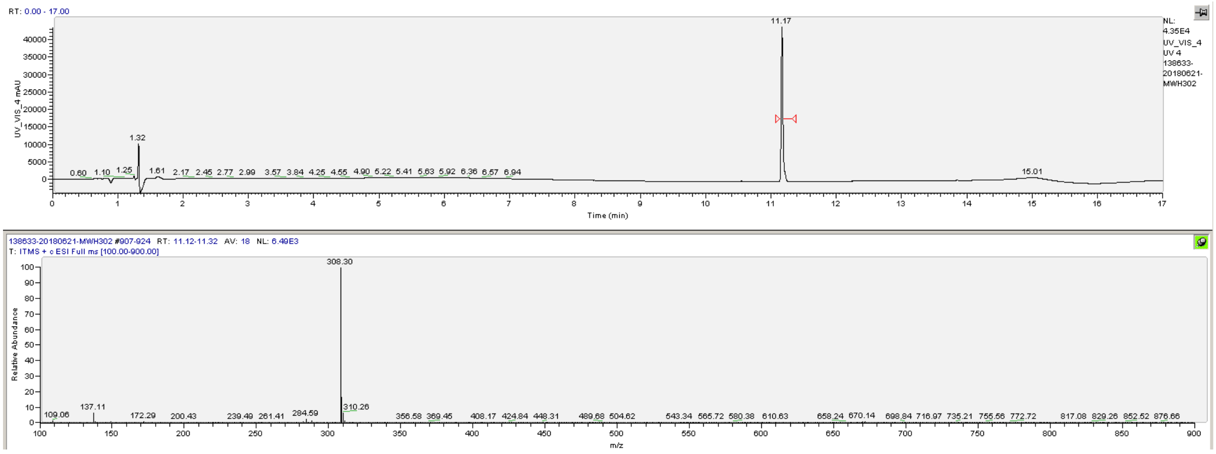

Supplementary Figure 18: *LC trace of **5e**, bottom: mass spectrum at selected retention time.*

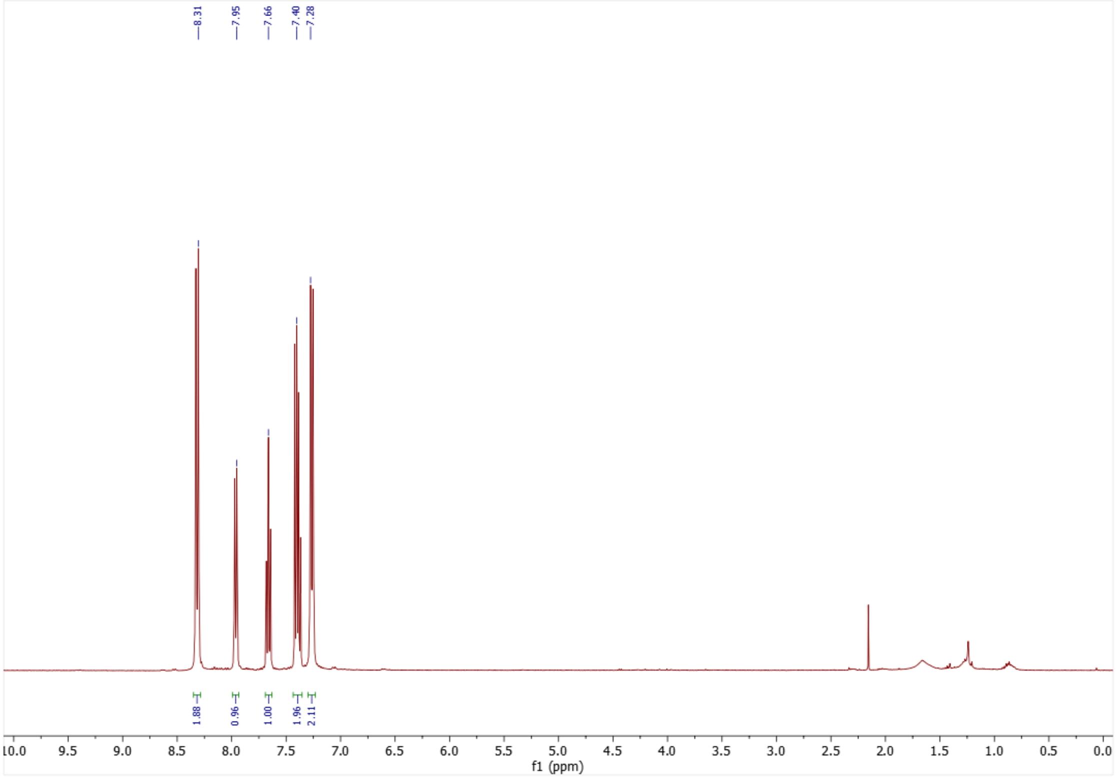

Supplementary Figure 19: *^{1}H NMR spectrum of **1f** in $CDCl_3$*

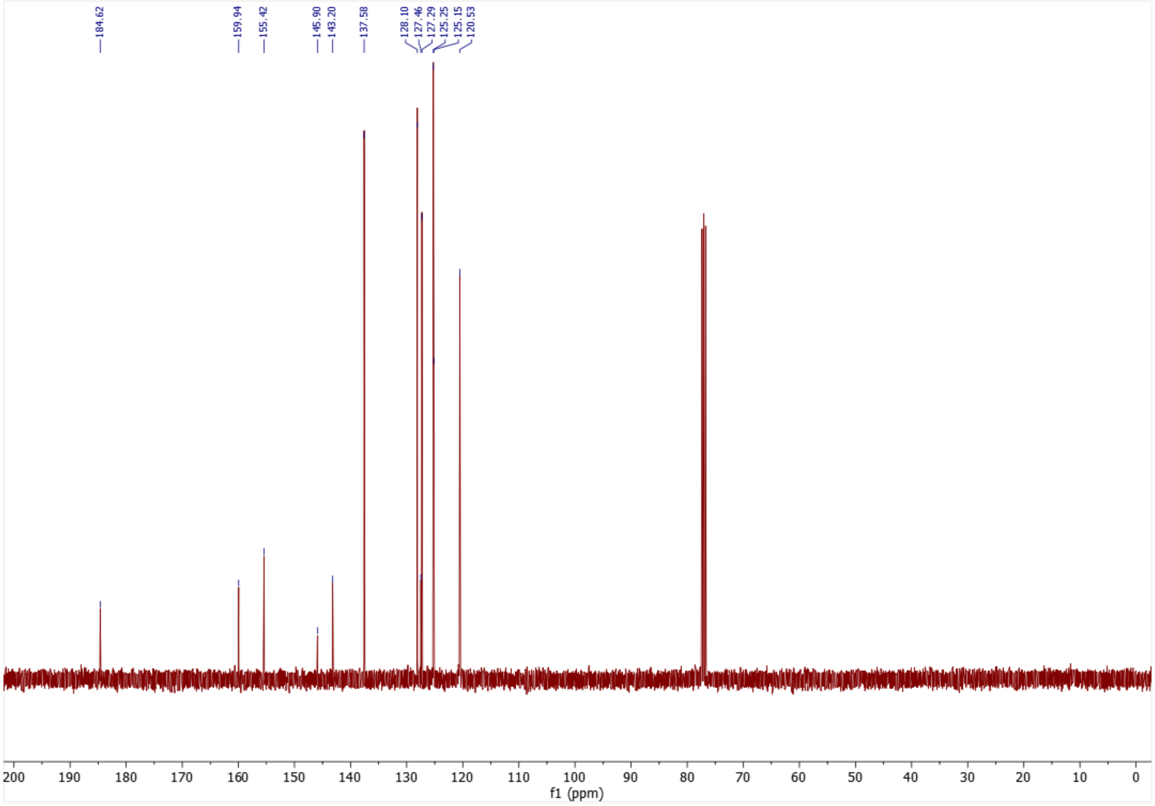

Supplementary Figure 20: *^{13}C NMR spectrum of **1f** in $CDCl_3$*

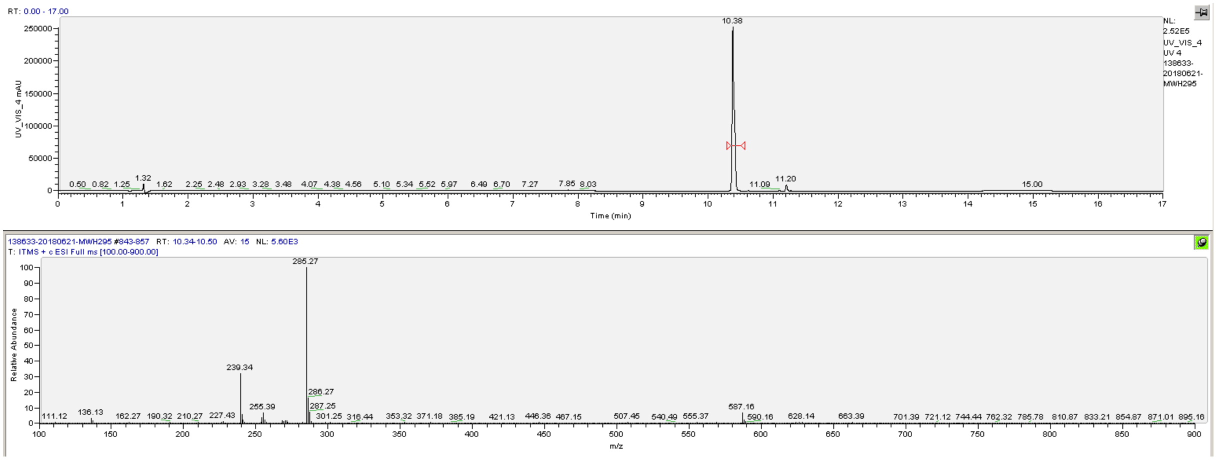

Supplementary Figure 21: *Top: LC trace of **1f**, bottom: mass spectrum at selected retention time.*

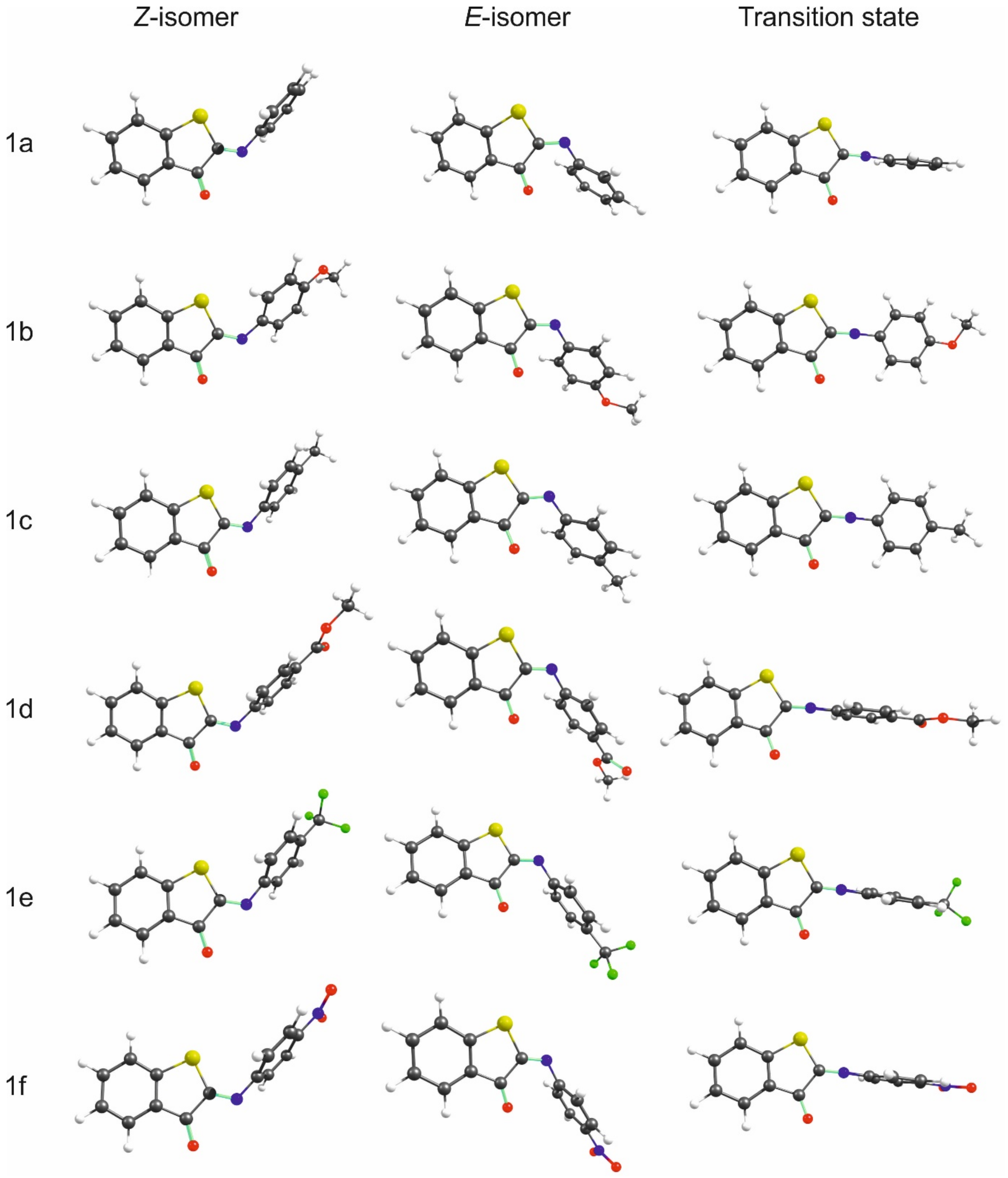

Supplementary Figure 22: *Structures of the Z-isomer, E-isomer and transition state of ITIs* ***(1a-1f)*** *in methanol optimized at the SMD-M06-2X/6-31+G(d) level of theory.*

Supplementary Note 1: Ordering of the states in gas phase

One of the well-known drawbacks of TD-DFT method is to determine the ordering of the state, especially for states having a different nature.[3] Therefore preliminary calculations are crucial to first validate the exchange correlation functional, especially in the present study as transient absorption spectra revealed that the optimal kinetic model to explore the ES relaxation is considering the second excited state (S_2) for *Z* isomer. In ITI, compared to HTI, the aza nitrogen atom is inducing a larger twist of the central bonds in both Z and E isomers, therefore a larger mixing of the orbitals (n, π_1 and π_2) upon excitation is observed. DFT molecular orbitals are depicted in Supplementary Figure 23. A comparison between high-level wavefunction-based theory (CC2[4] and ADC(2)[5]) and M06-2X and PBE0[6,7] exchange correlation functionals for both isomers have been performed in gas phase. As less Hartree-Fock exchange is present in the PBE0 functional, less mixing of between orbitals is expected compared to M06-2X or CC2/ADC(2). Supplementary Tables 1 and 2 present the results for the three lowest singlet excited states. For the *Z* isomer, CC2 and ADC(2) calculations indicate that the bright state is the second one, with a large oscillator strength. Such an ordering is only reproduced by M06-2X, whereas PBE0 with less pronounced mixing of MOs finds the reverse ordering of the two lowest excited states. Let us underline that for both isomers, the t2 amplitudes in the CC2 and ADC(2) calculations are below 12%, indicating that double excitations do not have the major contribution, thus corroborating the appropriateness of TD-DFT. For the *E* isomer, all methods consistently predict that VEE for the first excited state is notably smaller compared to the bright state of the *Z* isomer oscillator. Note that for the *E* form, the oscillator strength of the $S_0 \rightarrow S_1$ transition is rather small even with wavefunction-based methods. To better understand the relationships between the oscillator strength *f* and the planarity of the system, the dependence of the oscillator strength on the θ_1 and θ_2 dihedral angles has also been explored (Supplementary Figure 23). Although the planarization of the structure leads to increased values of *f*, it is not energetically favorable, so the Boltzmann averaging over distorted structures leads only to a slight increase of the oscillator strength.

Supplementary Table 1. *Ordering of the lowest singlet excited states for Z-ITI in gas phase obtained with the M06-2X/6-311++G(2df,2p)//M06-2X/6-31+G(d) and CC2(ADC(2))/aug-cc-pVTZ//M06-2X/6-31+G(d) approaches. Involved MOs are indicated when their weight is higher than 0.2. The most important MOs are displayed in Supplementary Figure 23.*

state	VEE (eV)	VEE(nm)	f	<S**2>	MOs (weight)	Type
**M06-2X**						
1	3.00	413	0.010	0.0	59 → 63 (0.56)	$^1(n,\pi^*)$
					61 → 63 (-0.27)	mixed
					62 → 63 (0.23)	$^1(\pi, \pi^*)$
2	3.38	367	0.114	0.0	62 → 63 (0.62)	$^1(\pi,\pi^*)$
3	3.69	336	0.010	0.0	61 → 63 (0.57)	mixed
**PBE0**						
1	2.87	432	0.078	0.0	59 → 63 (0.32)	$^1(n,\pi^*)$
					62 → 63 (0.59)	$^1(\pi,\pi^*)$
2	3.03	409	0.031	0.0	59 → 63 (-0.39)	$^1(n,\pi^*)$
					61 → 63 (0.45)	mixed
					62 → 63 (0.36)	$^1(\pi,\pi^*)$
3	3.34	371	0.030	0.0	59 → 63 (0.45)	$^1(n,\pi^*)$
					61 → 63 (0.49)	mixed
**CC2/aug-cc-pVTZ**						
1	3.05	407	0.018	0.0	58 → 64 (0.72)	
					57 → 64 (0.39)	
					62 → 64 (0.39)	
					56 → 64 (0.20)	
2	3.21	386	0.085	0.0	62 → 64 (-0.76)	
					61 → 64 (0.43)	

					56 → 64 (0.30)
					58 → 64 (0.23)
3	3.59	345	0.025	0.0	61 → 64 (0.73)
					56 → 64 (-0.48)
					62 → 64 (0.21)
**ADC(2)/aug-cc-pVTZ**					
1	2.92	425	0.003	0.0	58 → 64 (0.74)
					57 → 64 (0.40)
					56 → 64 (0.32)
2	3.20	387	0.099	0.0	62 → 64 (0.84)
					61 → 64 (-0.32)
					56 → 64 (-0.30)
3	3.55	349	0.014	0.0	61 → 64 (0.78)
					56 → 64 (-0.44)

Supplementary Table 2. *Ordering of the lowest singlet excited states for E ITI in gas phase obtained with the M06-2X/6-311++G(2df,2p)//M06-2X/6-31+G(d)* and CC2(ADC(2))/aug-cc-pVTZ// *M06-2X/6-31+G(d) approach*es. *Involved MOs are indicated when their weight is higher than 0.2. The most important MOs are displayed in Figure Supplementairy Figure 23.*

state	VEE (eV)	VEE(nm)	f	<S**2>	MOs (weight)	Type
**M06-2X**						
1	2.37	522	0.021	0.0	58 → 63 (0.26)	$^1(n,\pi^*)$
					61 → 63 (-0.24)	mixed
					62 → 63 (0.55)	$^1(\pi,\pi^*)$

2	3.30	375	0.035	0.0	61 → 63 (0.61)<br>62 → 63 (0.33)	mixed<br>$^1(\pi,\pi^*)$
3	3.91	317	0.077	0.0	56 → 63 (0.29)<br>58 → 63 (0.35)<br>61 → 63 (0.22)<br>62 → 63 (0.20)	mixed<br>$^1(n,\pi^*)$<br>mixed<br>$^1(\pi,\pi^*)$
**PBE0**						
1	2.19	565	0.037	0.0	62 → 63 (0.66)	$^1(\pi,\pi^*)$
2	2.91	426	0.021	0.0	61 → 63 (0.67)	mixed
3	3.41	364	0.051	0.0	60 → 63 (0.67)	$^1(\pi,\pi^*)$
**CC2/aug-cc-pVTZ**						
1	2.37	523	0.0127	0.0	62 → 64 (0.71)<br>58 → 64 (0.44)<br>57 → 64 (0.39)	
2	3.16	392	0.022	0.0	61 → 64 (0.87)<br>62 → 64 (-0.31)	
3	3.91	317	0.038	0.0	62 → 64 (-0.43)<br>56 → 64 (0.40)<br>58 → 64 (0.38)<br>57 → 64 (0.34)<br>61 → 64 (-0.30)	
**ADC(2)/aug-cc-pVTZ**						
1	2.30	539	0.009	0.0	62 → 64 (0.69)<br>58 → 64 (0.45)<br>57 → 64 (0.39)	
2	3.16	392	0.021	0.0	61 → 64 (0.86)<br>62 → 64 (-0.34)	

3	3.80	326	0.032	0.0	62 → 64 (-0.43)
					56 → 64 (0.42)
					58 → 64 (0.33)
					57 → 64 (-0.32)
					61 → 64 (0.30)

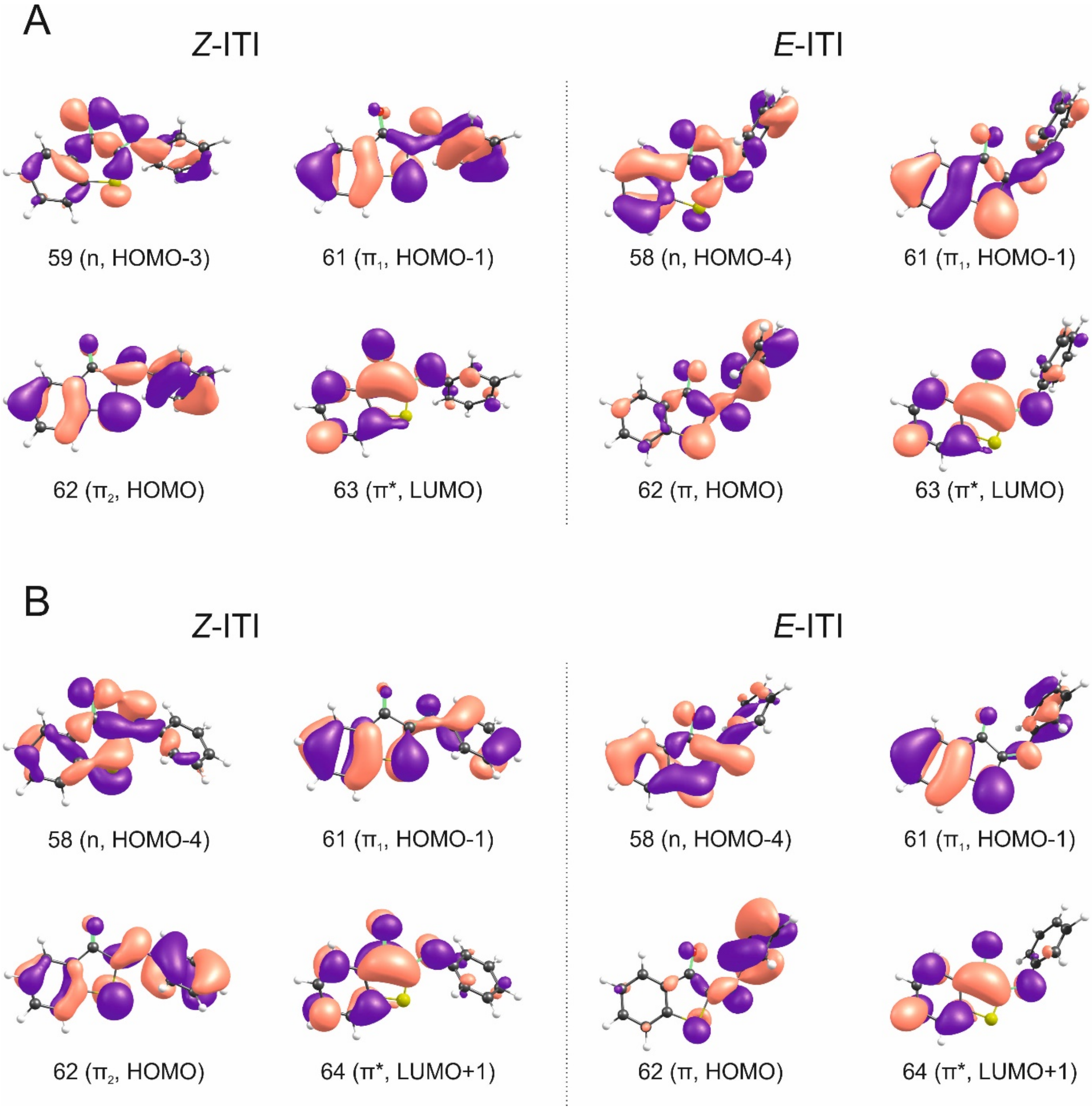

Supplementary Figure 23. *A: The most important MOs involved in the singlet excitations for Z (left) and E (right) ITI (**1a**) obtained with the TD-M06-2X/6-311++G(2df,2p)//M06-2X/6-31+G(d) approach. B: Hartree-Fock MOs involved in the excitation for for Z (left) and E (right) ITI (**1a**) obtained with the aug-cc-pVTZ basis set that were used in post-HF CC2 and ADC(2) calculations referred to in Supplementary Table 1 and 2.*

Supplementary Table 3. *Ordering of the lowest singlet excited states for Z-ITI (**1a**) in different solvents obtained with the SMD-TD-M062X/6-311++G(2df,2p)//SMD-M06-2X/6-31+G(d) approach. All vertical excitation energies were computed using the cLR formalism in the non-equilibrium regime. The most important MOs (weight > 0.20) are displayed in Supplementary Figure 24.*

solvent	transition	VEE (eV)	VEE(nm)	f	<S**2>	MOs (weight)	Type
CHX	$S_0 \rightarrow S_1$	3.04	408	0.026	0.0	58 → 63 (0.46)<br>59 → 63 (0.30)<br>61 → 63 (0.26)<br>62 → 63 (-0.29)	mixed
	$S_0 \rightarrow S_2$	3.31	373	0.312	0.0	62 → 63 (0.61)	$^1(\pi,\pi^*)$
	$S_0 \rightarrow S_3$	3.67	338	0.029	0.0	56 → 63 (-0.23)<br>61 → 63 (0.56)	$^1(\pi,\pi^*)$
	$S_0 \rightarrow S_4$	4.73	262	0.004	0.0	60 → 63 (0.62)	$^1(\pi,\pi^*)$
TOL	$S_0 \rightarrow S_1$	3.04	407	0.028	0.0	58 → 63 (0.52)<br>61 → 63 (0.26)<br>62 → 63 (-0.30)	mixed
	$S_0 \rightarrow S_2$	3.30	374	0.132	0.0	58 → 63 (0.20)<br>62 → 63 (0.61)	$^1(\pi,\pi^*)$
	$S_0 \rightarrow S_3$	3.67	338	0.032	0.0	56 → 63 (-0.23)<br>61 → 63 (0.56)	$^1(\pi,\pi^*)$
	$S_0 \rightarrow S_4$	4.73	262	0.005	0.0	60 → 63 (0.62)	$^1(\pi,\pi^*)$
CHL	$S_0 \rightarrow S_1$	3.08	402	0.039	0.0	58 → 63 (0.52)<br>61 → 63 (0.24)<br>62 → 63 (-0.34)	mixed

	$S_0 \rightarrow S_2$	3.28	378	0.106	0.0	58 → 63 (0.25)<br>61 → 63 (0.23)<br>62 → 63 (0.59)	$^1(\pi,\pi^*)$
	$S_0 \rightarrow S_3$	3.67	338	0.042	0.0	56 → 63 (0.25)<br>58 → 63 (-0.22)<br>61 → 63 (0.54)	$^1(\pi,\pi^*)$
	$S_0 \rightarrow S_4$	4.69	264	0.189	0.0	59 → 63 (0.66)	$^1(\pi,\pi^*)$
MeOH	$S_0 \rightarrow S_1$	3.12	398	0.097	0.0	58 → 63 (-0.34)<br>62 → 63 (0.58)	mixed
	$S_0 \rightarrow S_2$	3.26	381	0.022	0.0	58 → 63 (0.44)<br>61 → 63 (0.37)<br>62 → 63 (0.35)	mixed
	$S_0 \rightarrow S_3$	3.65	340	0.053	0.0	56 → 63 (-0.27)<br>58 → 63 (-0.28)<br>61 → 63 (0.51)	mixed
	$S_0 \rightarrow S_4$	4.58	271	0.196	0.0	59 → 63 (0.67)	$^1(\pi,\pi^*)$
DMSO	$S_0 \rightarrow S_1$	3.12	398	0.032	0.0	58 → 63 (0.54)<br>61 → 63 (0.24)<br>62 → 63 (-0.32)	mixed
	$S_0 \rightarrow S_2$	3.28	379	0.094	0.0	58 → 63 (0.23)<br>61 → 63 (0.21)<br>62 → 63 (0.60)	$^1(\pi,\pi^*)$
	$S_0 \rightarrow S_3$	3.68	337	0.045	0.0	56 → 63 (-0.24)<br>58 → 63 (-0.21)<br>61 → 63 (0.56)	$^1(\pi,\pi^*)$
	$S_0 \rightarrow S_4$	4.66	266	0.182	0.0	59 → 63 (0.66)	$^1(\pi,\pi^*)$

Supplementary Note 2:

In Supplementary Figure 24 the MOs of the *Z* and *E* forms of ITI (**1a**) in MeOH are shown. As presented in Supplementary Table 3, only the ordering of states in MeOH with SMD is changed for the *Z* form. In the following, the $S_0 \rightarrow S_1$ state is the investigated transition in MeOH for both *E* and *Z* isomer. When looking at the electronic density difference plots between ES and GS (Supplementary Figure 25) we do observe similar loss (blue) and gain (red) of the density upon excitation for both forms. Let us also note that the change of the state ordering in methanol was not observed, when the C-PCM approach (suitable for highly polar solvents) was applied. Nevertheless, to be consistent, we used the SMD model for all solvents.

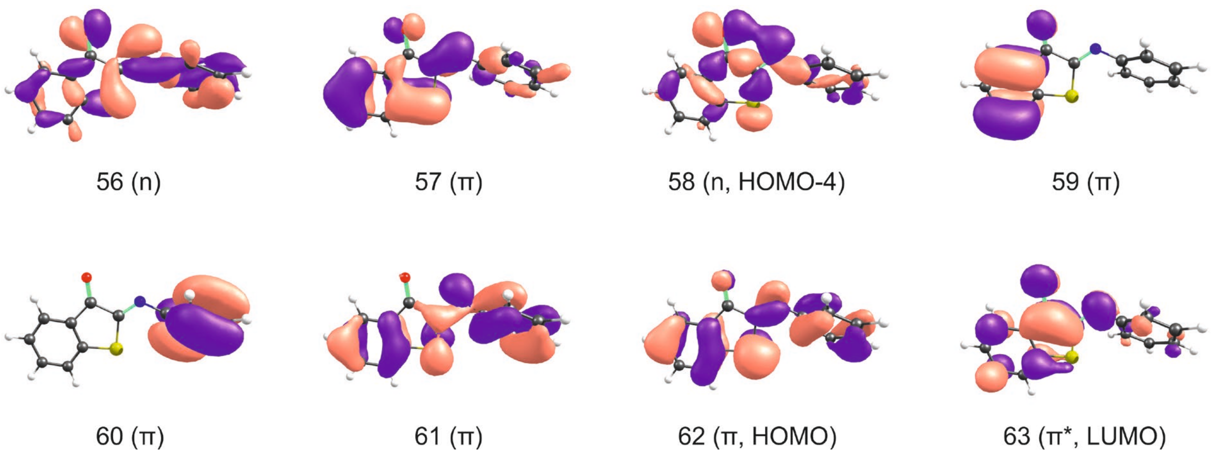

Supplementary Figure 24. *The most important MOs involved in the singlet excitations for Z-ITI* ***1a*** *in MeOH obtained with the M06-2X/6-311++G(2df,2p)/SMD//M06-2X/6-31+G(d)/SMD approach.*

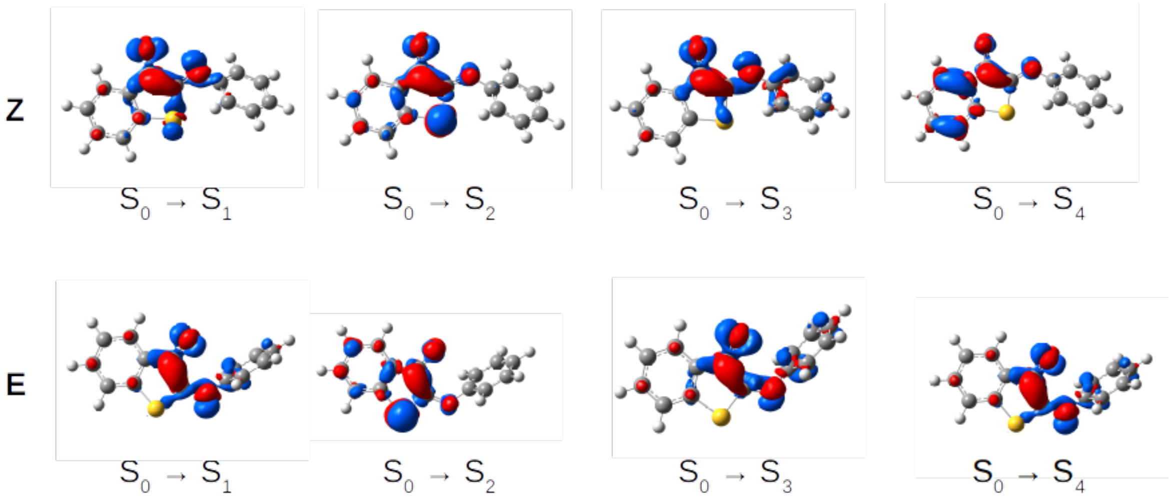

Supplementary Figure 25. *Electronic density difference (EDD) plots for the four lowest singlet excitations for ITI (**1a**) in methanol obtained with the SMD-M06-2X/6-311++G(2df,2p)/SMD-M06-2X/6-31+G(d) approach. The blue (red) regions correspond to decrease (increase) in electron density upon electronic transition. A contour threshold of 0.003 a.u. has been applied. Forces acting on individual atoms after vertical excitations to S_1 and S_2 states of **1a** in its Z-form*

Supplementary Note 3:

To gain insights into structural changes occurring after vertical excitations to S_1 (mixed character with significant n-π* contribution) and S_2 (high π-π* character) states of **1a** in its *Z*-form, the forces acting on individual atoms have been visualized in terms of changes during first ES optimization steps (Supplementary Figure 26). Let us note that both ES optimizations lead in later stages towards quasi-degenerate states (conical intersection), where the application of TD-DFT is no more appropriate.

Supplementary Figure 26 and the data in Supplementary Table 4 show that in both solvents the S_1 state undergoes more pronounced changes compared to the S_2 state. In particular, the angle C2-N4-C5 significantly increases, which is apparently related to change of the charge distribution (hybridization state) on the nitrogen atom due to significant n-π* contribution to S_1. The "opening" of the structure is accompanied with changes of the dihedral angle θ_1 (C1-C2-

N4-C5) leading towards the isomerization. On the other hand, the structure in the S_2 state is changed only very slightly (at least in this initial stages) indicating that much weaker forces act on atoms in this state. In both states the C2-N4 bond gets prolonged upon excitation which can be rationalized by the presence of a nodal plane perpendicular to the bond in the π^* (LUMO) orbital (see Figure 2D).

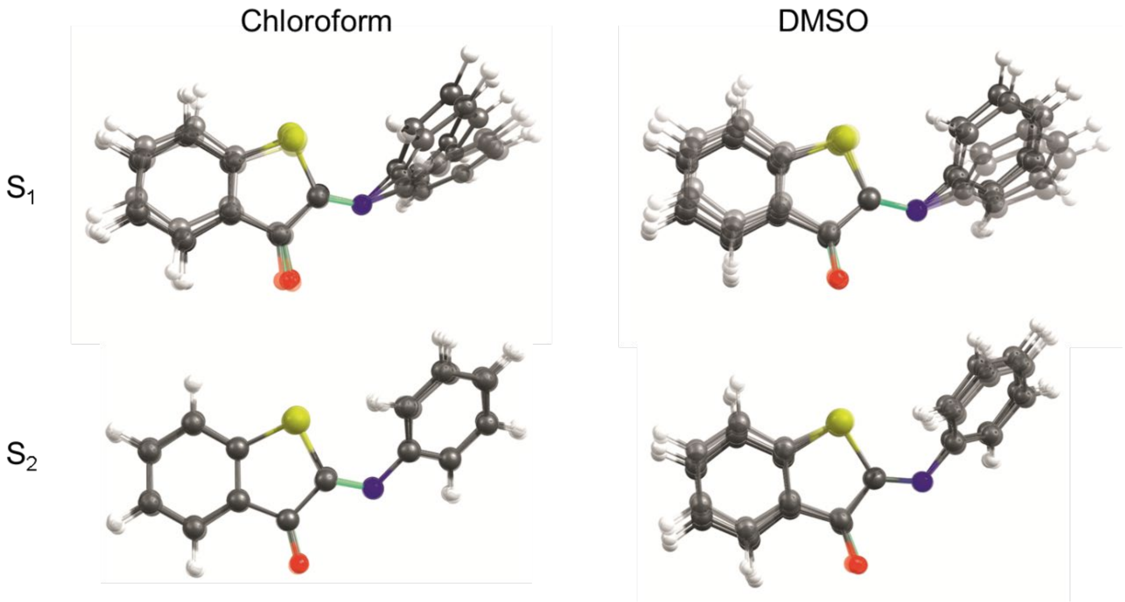

Supplementary Figure 26. *Structural changes of **Z-1a** in S_1 and S_2 states after vertical excitation during first optimization steps obtained at the SMD-TD-M06-2X/6-31+G(d) level of theory (solvents: chloroform (left) and DMSO (right)).*

Supplementary Table 4. *Structural changes of **Z-1a** in S_1 and S_2 states after vertical excitation during first optimization steps obtained at the SMD-TD-M06-2X/6-31+G(d) level of theory in terms of key parameters as defined in Figure 2C.*

**Solvent**	**chloroform**					
**State**	**S_1**			**S_2**		
Iter.	C2-N4	C2-N4-C5	θ_1	C2-N4	C2-N4-C5	θ_1
0 (GS)	1.262	122.6	180.0	1.262	122.6	180.0
1	1.274	130.6	-171.8	1.288	122.0	-178.6
2	1.301	145.4	-155.0	1.285	125.0	178.8
3	1.300	148.5	-145.6	1.280	124.1	178.0
**Solvent**	**DMSO**					
**State**	**S_1**			**S_2**		
Iter.	C2-N4	C2-N4-C5	θ_1	C2-N4	C2-N4-C5	θ_1
0 (GS)	1.263	121.9	180.0	1.263	121.9	180.0
1	1.276	129.6	-171.8	1.288	121.2	-179.7
2	1.303	143.0	-156.8	1.333	126.2	-168.0
3	1.301	145.3	-148.3	1.310	123.6	-174.0

Supplementary Note 4: Solvent effects on thermal relaxation of parent ITI

Supplementary Table 5 reports relative energies of *E* and *Z* forms as well as of the GS transition state (TS) for a backward reaction ($E \rightarrow Z$) in all investigated solvents (see Supplementary Figure 27 for the two possible TS structures). The relative stability of the *E* form w.r.t. the *Z* form slightly decreases with the increasing polarity of solvent. MP2/cc-pVTZ calculations indicate that the relative stability of *E*/*Z* forms is well described at the M06-2X/6-31+G(d) level of theory (both in gas phase and in methanol). Supplementary Figure 28 presents the GS potential energy surface (PES) showing that only the in-plane TS structure (N-inversion) was found. Out-of-plane distortion from the in-plane-TS structure leads to an increase of energy. A difference in the TS structures is observed in the gas phase (planar) compared to the solvent (twisted). This effect is related to the interplay between the stabilization effects due to π-conjugation and different polarity of the planar and twisted structures (the latter is more polar, see Supplementary Table 6). In fact, for less polar solvents (CHX, TOL, CHL, ETAc) the planar TS structure was also found, but it was less stable than the twisted one. Let us notice, however, that the energy difference between the twisted and planar TS structures for non-polar CHX is only 0.1 kcal/mol. The dependence of dipole moment of the TS in the ground state on the twisting angle is presented in Supplementary Figure 29. The changes can be explained by a different charge distribution in the planar and twisted structures related to the different valence picture on the nitrogen atom (Supplementary Figure 30).

Supplementary Table 5. *Ground state relative energies (in kcal/mol) of the Z/E-isomers and transition state of ITI* ***1a*** *with respect to the Z form calculated applying the SMD-M06-2X/6-31+G(d) approach. The values in the parentheses were obtained at the MP2/cc-pVTZ level.*

	Relative energy (kcal/mol)							
**Solvent**	**gas**	**CHX**	**TOL**	**CHL**	**ETAc**	**MET**	**DMS**	**WAT**
ε_r	1.00	2.02	2.37	4.71	5.99	32.61	46.83	78.36
Z form	0.0	0.0	0.0	0.0	0.0	0.0	0.0	0.0
TS (planar)[a]	16.9 (20.3)	17.1	17.2	18.0	18.2	NA	NA	NA
TS (twisted)	NA	17.0	17.0	17.6	17.6	18.7 (21.5)	18.0	20.0
E form	3.7 (3.8)	4.2	4.3	4.3	4.4	4.3 (4.1)	4.5	4.3
Thermal relaxation barrier	13.2[a]	12.8	12.7	13.3	13.1	14.4	13.5	15.7
	**Relative Gibbs energy (kcal/mol)**							
	**gas**	**CHX**	**TOL**	**CHL**	**ETAc**	**MET**	**DMS**	**WAT**
ε_r	1.00	2.02	2.37	4.71	5.99	32.61	46.83	78.36
Z form	0.0	0.0	0.0	0.0	0.0	0.0	0.0	0.0
TS-in-plane	16.0[a]	16.1	16.2	16.5	16.5	17.1	16.8	18.4
E form	3.1 (3.4)	3.1	3.2	3.0	3.4	3.7	4.0	4.1
Thermal relaxation barrier	13.0[a]	13.0	13.0	13.5	13.1	13.4	12.8	14.3

[a] *TS structure is not twisted only in the gas phase (see Supplementary Figure 27)*

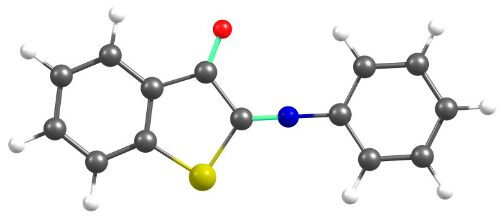

planar TS structure (gas phase)

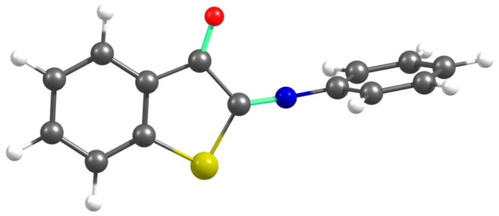

twisted TS structure (solvent)

Supplementary Figure 27. *Ground state structures of the transition state of ITI **1a** in gas phase (left) and in solvents (right).*

Supplementary Table 6. *Ground state dipole moments (in Debyes) of the E, Z and TS of ITI **1a** in different solvents calculated applying the SMD-M06-2X/6-31+G(d) approach.*

	Dipole moment (D)							
**Solvent**	**gas**	**CHX**	**TOL**	**CHL**	**ETAc**	**MET**	**DMS**	**WAT**
ε_r	1.00	2.02	2.37	4.71	5.99	32.61	46.83	78.36
Z form	3.99	4.60	4.74	4.74	5.38	6.47	5.92	6.69
TS-in-plane	1.20	1.30	1.33	1.52	1.47	NA	NA	NA
TS-in-plane (twisted)	NA	3.85	3.97	4.44	4.49	5.25	4.92	5.35
E form	3.20	3.86	4.02	4.51	4.66	5.27	5.12	5.39

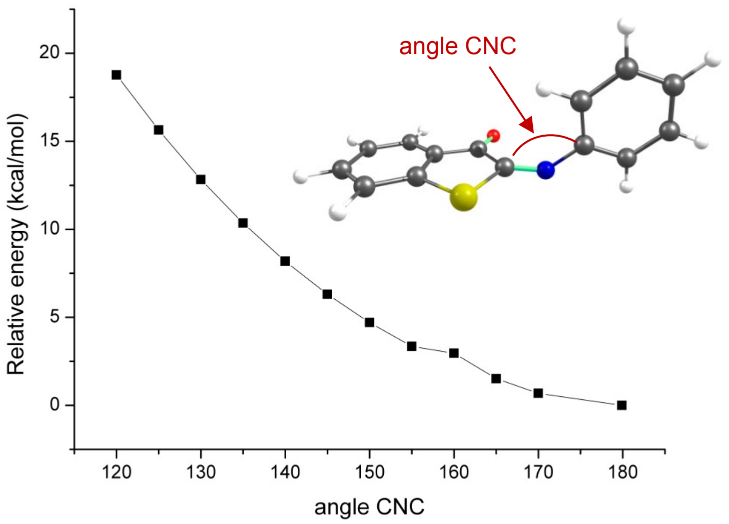

Supplementary Figure 28. *Ground state potential energy scan for the out-of-plane distortion from the in-plane-TS structure of **1a** (gas phase) calculated at the M06-2X/6-31+G(d) level. All internal coordinates except for the CNC angle were allowed to relax during the constrained geometry optimization.*

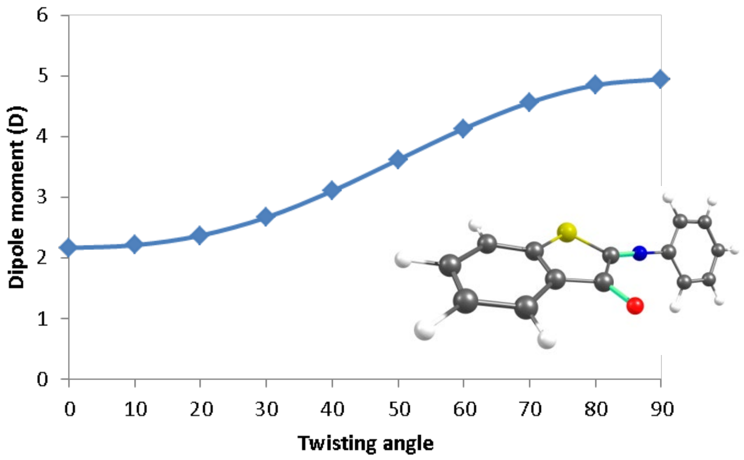

Supplementary Figure 29. *Dependence of the ground state dipole moment (in D) on the twisting angle θ_2 for the TS structure of **1a** in MeOH calculated with the SMD-M06-2X/6-31+G(d) approach.*

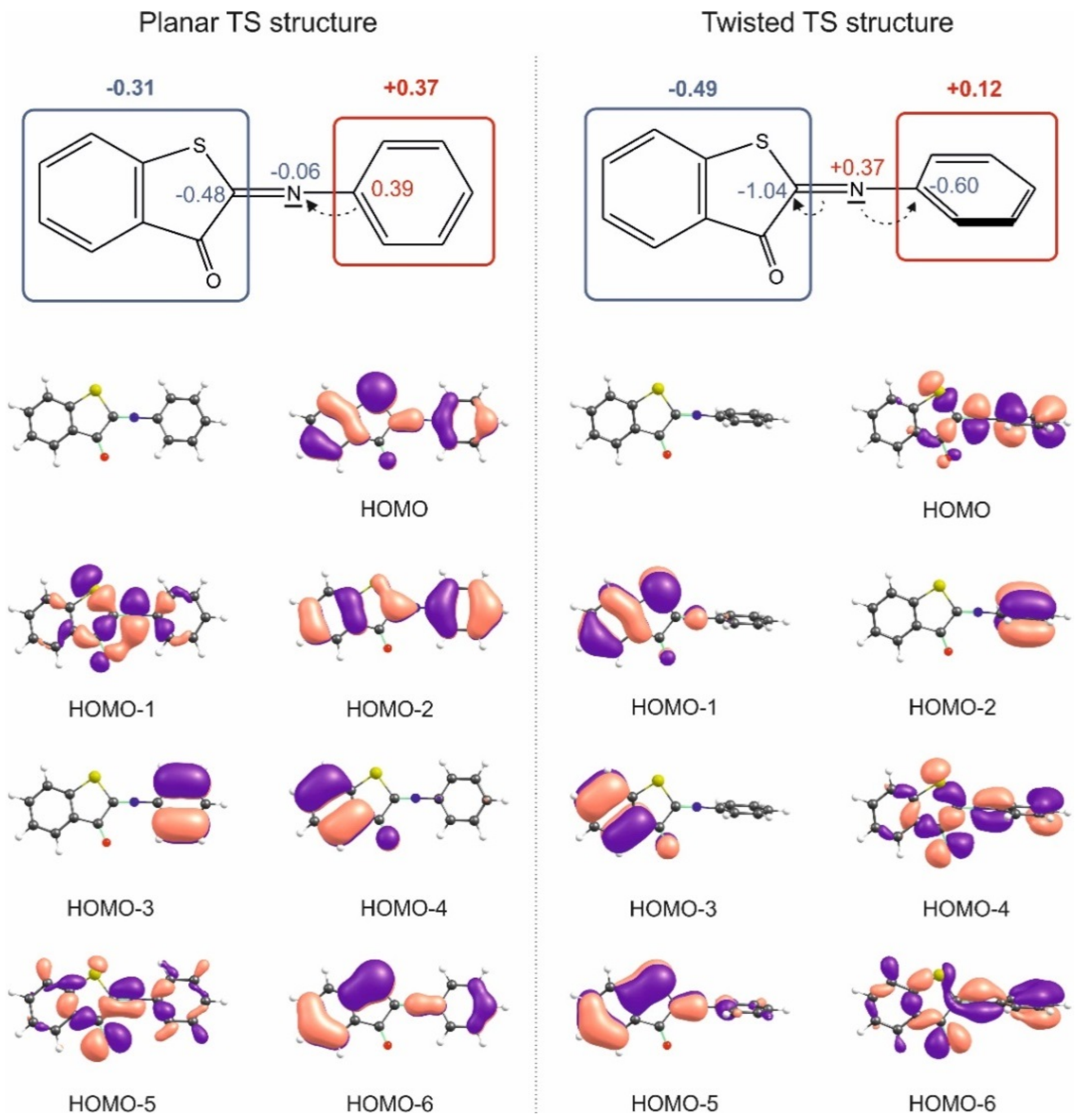

Supplementary Figure 30. *Origin of differences in polarity between the planar (left) and twisted (right) TS structures of* ***1a****. Whereas in the former the lone pair on nitrogen does not participate in bonding (cf. HOMO-1) and the second p orbital enables efficient charge delocalization bridging the two moieties (HOMO and HOMO-6), in the twisted structure both p orbitals form bonds (cf. HOMO-4 and HOMO-5) creating a positive charge on nitrogen.*

Supplementary Note 5. Substitution effects on absorption spectra of ITIs

For the *Z* isomer, theoretical VEEs are in agreement with the obtained experimental λ max. Compared to **1a**, a bathochromic shift of +19 and +5 nm is experimentally observed for ITI substituted by an EDG such as **1b** and **1c** while a hypsochromic shift is reached for ITI with an EWG, that is **1d** (-2 nm) and **1e** (-5 nm). Theoretically, a shift of +15 and +8 nm is calculated for **1b** and **1c** compared to **1a** while hypsochromic shifts of -2 and -7 nm are obtained for **1d** and **1e**, respectively. Geometrically, there is solely a slight modification of θ_1 (1°) for the *Z* isomer which is not sufficient to explain the observed auxochromic shifts (see Fig. 5 of the main text). However, θ_2 changes depending on the nature of the substituting group, e.g. θ_2 is smaller (larger) than the one of the unsubstituted form (**1a**) for ITI with EDG (EWG). θ_2 of **1b** and **1c** is, respectively, 38.6° and 50.3°, **1b** being the less twisted photochrome. Compounds **1d** and **1e** are characterized by a θ_2 angle of 60.8° and 62.0°, respectively. An increase (decrease) of λ_{max} for EDG (EWG) substituents is, therefore, obtained compared to 1a as 1b and **1c** (**1d** and **1e**) are less (more) "twisted" around the –N=C- central single bond.

Looking at the computed charge transfer parameters (Supplementary Table 8), relatively large charge transfer (though still smaller compared to typical push-pull molecules) upon excitation is observed for *Z* **1b**, **1c** with a μ_{CT} of 6.74 and 5.34 D. The amount of charge transfer is similar for all substituents in *Z* (0.66 e) and *E* (0.75 e) forms. The distance is obviously slightly larger for **1b** and **1c** as the central -N-C- bond is less twisted in the *Z* form. Supplementary Figure 31 shows a slight decrease of the electronic density upon excitation onto the substituted ring for **1a**, **1b** and **1c** while for EWG the gain/loss of density is only observed onto the thioindigo moieties. Interestingly, the variation in the ES and GS dipole moment ($\Delta\mu_{ES\text{-}GS}$) for *Z* form is larger and positive for **1b** (4.43 D) and **1c** (2.64 D) which re-inforce the geometrical contribution explaining the bathochromic shift, e.g. the ES of both compounds will be more stabilized than other substituents due to their larger dipole moment, especially in methanol. Large $\Delta\lambda_{max}$

values, and the corresponding auxochromic shifts observed in experiment and consistently reproduced by theoretical calculations (Fig. 5 in the main text), can be related to the modification of the dihedral angles between both isomers. For instance, in **1b** (with remarkably large experimental and theoretical values of $\Delta\lambda_{max}$ of +105 nm and +120 nm, respectively), θ_1 and θ_2 differ in *Z* and *E* (the values for *E* are larger). As mentioned before, θ_2 is mainly responsible for explaining the auxochromic effect, which is also the case for the *E* isomer. We note, however, that the $\theta 2$ values in E are larger than those in the *Z* form. However, as for the *Z* form, these values are smaller (larger) for **1b** and **1c** (**1d-f**) compared to **1a** explaining the observed bathochromic (hypsochromic) shifts. In addition, a twist around the -C=N- central double bond (θ_1 angle) in the E form is observed going from 0.26° (**1d**) to 12.4° (**1b**) inducing destabilization of the GS (and of the HOMO). In addition to the geometrical effect, Supplementary Table 8 indicates a different behavior of the GS and ES dipole moments of the *E* form compared to the *Z* form. Both μ_{ES} and μ_{GS} are smaller for the *E* isomer than the *Z* isomer for all ITIs, but the decrease is more pronounced for the ES values. Consequently, for EWG (**1d-f**) μ_{ES} is smaller than μ_{GS} with $\Delta\mu$ < -4 D (as well for **1a**) for *E* isomer while for EDG $\Delta\mu$ is positive for **1b** ($\mu_{ES} > \mu_{GS}$) meaning that the ES dipole moment is larger than the GS dipole moment in MeOH and slightly negative (1c) . Concerning the auxochromic effects for *E*, compared to **1a**, shifts of +38 and +33 nm (+28 and +14 nm) are observed experimentally (theoretical) for **1b** and **1c**, while for **1d**, **1e** and **1f** the shifts are -12, -15 and -14 nm (-21, -23 and -30 nm), respectively.

Supplementary Table 7. *Optical properties of Z and E **1a-f** in MeOH obtained with the SMD-TD-M062X/6-311++G(2df,2p)//SMD-M06-2X/6-31+G(d) approach. All vertical excitation energies were computed using the cLR formalism in the non-equilibrium regime. Electronic density plots are displayed in Supplementary Figure 31.*

Isomer	ITI	transition	VEE (eV)	VEE (nm)	f	MOs (weight)	Type
Z	**1a**	$S_0 \rightarrow S_1$	3.11	398	0.098	62 → 63 (0.58)<br>58 → 63 (0.34)	mixed
	**1b**	$S_0 \rightarrow S_1$	3.00	413	0.264	70 → 71 (0.62)<br>66 → 71 (0.29)	mixed
	**1c**	$S_0 \rightarrow S_1$	3.06	406	0.145	66 → 67 (0.61)<br>62 → 67 (0.32)	mixed
	**1d**	$S_0 \rightarrow S_1$	3.11	399	0.092	66 → 67 (0.56)<br>62 → 67 (-0.15)	mixed
	**1e**	$S_0 \rightarrow S_1$	3.17	391	0.064	78 → 79 (0.55)<br>74 → 79 (0.37)	mixed
	**1f**	$S_0 \rightarrow S_1$	3.18	390	0.078	73 → 74 (0.53)<br>69 → 74 (-0.33)<br>72 → 74 (0.21)	mixed
E	**1a**	$S_0 \rightarrow S_1$	2.46	505	0.015	62 → 63 (0.55)<br>58 → 63 (-0.30)<br>61 → 63 (0.28)	mixed
	**1b**	$S_0 \rightarrow S_1$	2.32	533	0.074	70 → 71 (0.58)<br>66 → 71 (-0.32)	mixed
	**1c**	$S_0 \rightarrow S_1$	2.39	519	0.033	66 → 67 (0.58)<br>62 → 67 (-0.31)	mixed

**1d**	$S_0 \rightarrow S_1$	2.56	484	0.000	77 → 78 (0.52)	mixed
					76 → 78 (0.32)	
					73 → 78 (-0.31)	
**1e**	$S_0 \rightarrow S_1$	2.57	482	0.001	77 → 79 (0.56)	mixed
					74 → 79 (0.29)	
					78 → 79 (-0.26)	
	$S_0 \rightarrow S_2$	3.15	394	0.021	77 → 79 (0.29)	$^1(\pi,\pi^*)$
					78 -> 79 (0.64)	
**1f**	$S_0 \rightarrow S_1$	2.64	470	0.000	72 -> 74 (0.61)	mixed
					69 -> 74 (0.30)	
	$S_0 \rightarrow S_2$	3.15	395	0.025	73 -> 74 (0.70)	$^1(\pi,\pi^*)$

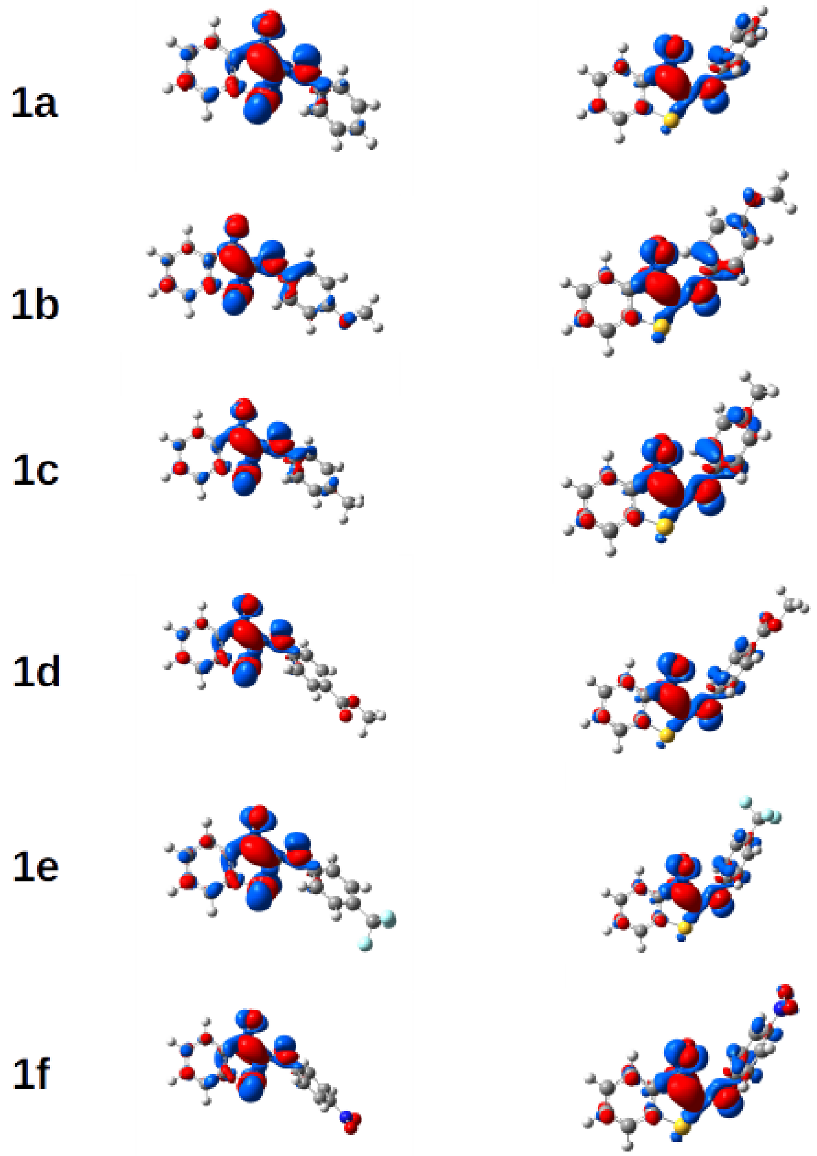

Supplementary Figure 31. *Electron density difference (EDD) plots for Z (left, first two transitions) and E (right, first transition) ITI (**1a-f**) in MeOH obtained with the SMD-M06-2X/6-311++G(2df,2p) //SMD-M06-2X/6-31+G(d) approach. The blue (red) regions correspond to decrease (increase) in electron density upon electronic transition. A contour threshold of 0.002 a.u. has been applied.*

Supplementary Table 8. *Charge transfer parameters (amount of charge q_{CT} in e, distance d_{CT} in Å and dipole moment μ_{CT} in D) as well as GS and ES dipole moment as obtained at the obtained with the SMD-TD-PBE0/6-311++G(2df,2p)//SMD-M06-2X/6-31+G(d) level of theory for Z and E substituted ITI in methanol.*

ITI	q_{CT}	d_{CT}	μ_{CT}	μ_{GS}	μ_{ES}	$\Delta\mu$
				Z		
**1a**	0.66	1.21	3.82	6.59	8.14	1.55
**1b**	0.68	2.07	6.74	5.07	9.49	4.43
**1c**	0.68	1.64	5.34	6.35	8.99	2.64
**1d**	0.65	0.82	2.56	5.68	6.53	0.85
**1e**	0.64	0.78	2.41	10.30	9.68	-0.62
**1f**	0.64	0.57	1.75	13.15	12.89	-0.26
				E		
**1a**	0.75	1.81	6.51	5.42	1.26	-4.17
**1b**	0.73	2.03	7.14	2.49	4.86	2.37
**1c**	0.75	1.94	6.95	4.18	3.17	-1.01
**1d**	0.77	1.82	6.72	7.39	2.94	-4.45
**1e**	0.76	1.70	6.15	9.37	3.56	-5.81
**1f**	0.75	1.71	6.17	12.18	6.33	-5.85

Supplementary Note 6: Substitution effects on thermal relaxation of ITIs

Substitution effects on the activation barrier of the thermal relaxation are presented in Supplementary Table 9. The structure of the TS of EDG ITIs derivatives is planar, whereas that of EWG derivatives is twisted as a result of interplay (competition) between the stabilization due to more efficient conjugation in the planar conformation and the stabilization of more polar twisted structure in the polar solvent (MeOH). EWG substituents enhance the interaction of the lone pair on nitrogen with the π-orbitals on the phenyl ring favoring the twisted structure (Supplementary Figure 32). The lowest barrier of the backward thermal relaxation for the NO_2 derivative (**1f**) can be rationalized by the strongly polar TS structure largely stabilized in methanol.

Supplementary Table 9. *Relative energies (in kcal/mol) of the Z/E isomers and TS of ITI derivatives (**1a-f**) in MeOH with respect to the Z form calculated applying the SMD-M06-2X/6-31+G(d approach.*

	**Relative energy (kcal/mol)**						**Relative Gibbs energy (kcal/mol)**					
**Compound**	**1a**	**1b**	**1c**	**1d**	**1e**	**1f**	**1a**	**1b**	**1c**	**1d**	**1e**	**1f**
R=	H	MeO	Me	COOMe	CF_3	NO_2	H	MeO	Me	COOMe	CF_3	NO_2
cis	0.0	0.0	0.0	0.0	0.0	0.0	0.0	0.0	0.0	0.0	0.0	0.0
TS-in-plane	18.7	17.6[a]	18.5[a]	16.4	17.0	14.8	17.1	16.6[a]	17.8[a]	15.9	16.1	14.6
trans	4.3	4.6	4.5	3.3	3.4	2.8	3.7	4.3	4.1	NA[b]	NA[b]	NA[b]
Thermal relaxation barrier	14.4	13.0	14.0	13.1	13.6	12.0	13.4	12.3	13.7	NA[b]	NA[b]	NA[b]

[a] *TS-in-plane structure is not twisted (see Supplementary Figure 32)*

[b] *Not sufficient accuracy to perform the vibrational analysis.*

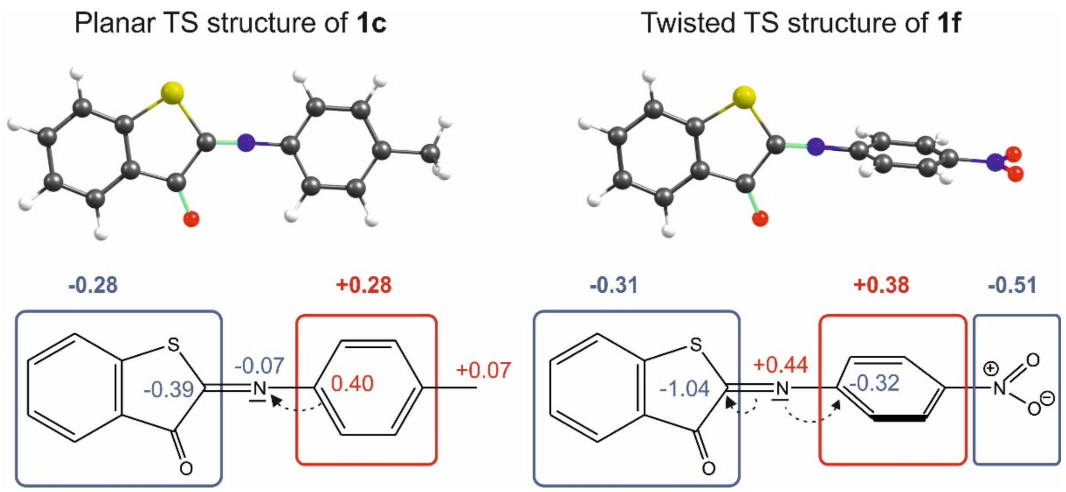

Supplementary Figure 32. *Ground state planar (left) and twisted (right) structures of the transition state of EDG (**1b,c**) and neutral/EWG (**1a, 1d-f**) ITIs, respectively. Mulliken atomic charges were obtained with the SMD-M06-2X/6-31+G(d) approach (solvent MeOH).*

Supplementary Table 10. *Ground state dipole moments (in Debyes) of the E/Z isomers and TS of ITI derivatives (**1a-f**) in MeOH calculated applying the SMD-M06-2X/6-31+G(d) approach.*

	**Dipole moment (Debye)**					
**Compound**	**1a**	**1b**	**1c**	**1d**	**1e**	**1f**
R=	H	MeO	Me	COOMe	CF_3	NO_2
cis	6.47	4.99	6.40	8.54	7.75	9.80
TS-in-plane	5.25	0.77[a]	1.93[a]	8.82	10.07	15.04
trans	5.27	3.23	4.61	8.02	9.13	12.56

[a] *TS structure is planar*

Supplementary Note 7. Substitutional effects on IR spectra of ITIs

Calculated IR spectra in gas phase have been performed using both harmonic and anharmonic approach. The anharmonic approach reproduces the main features of the experimental spectrum. The scaled harmonic spectrum is consistent with the anharmonic one, although the intensities of some peaks notably differ.

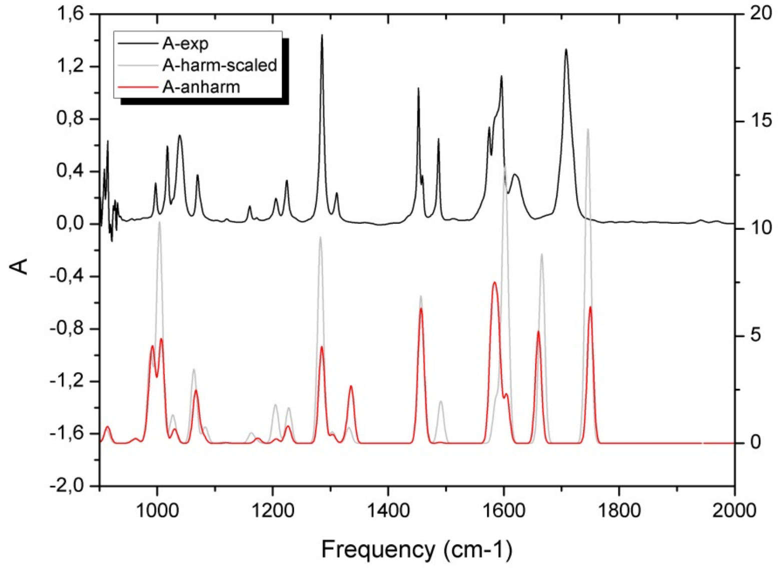

Supplementary Figure 33. *Comparison of experimental and theoretical (scaling factor f= 0.98) harmonic as well as fully anharmonic approximations, gas phase, B3LYP/6-31++G(d,p) GS IR spectra for Z-**1a**-ITI. The experimental spectrum was measured in $CDCl_3$ at room temperature.*

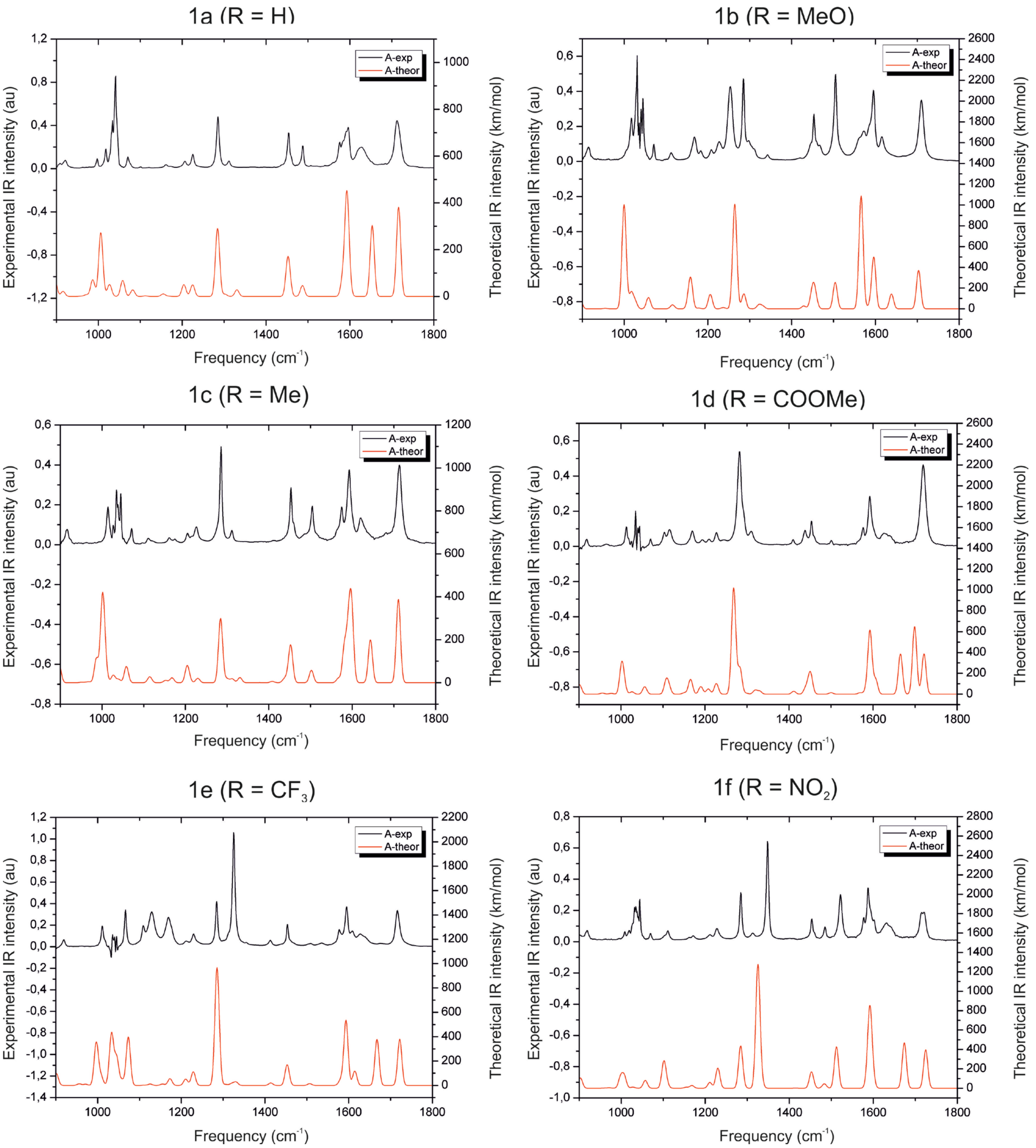

Supplementary Figure 34. *Comparison of simulated SMD-B3LYP/6-31++G(d,p)(solvent=ACN, harmonic approximation, scaling factor f= 0.98) and experimental (measured in ACN) GS IR spectra for Z-ITI derivatives at room temperature.*

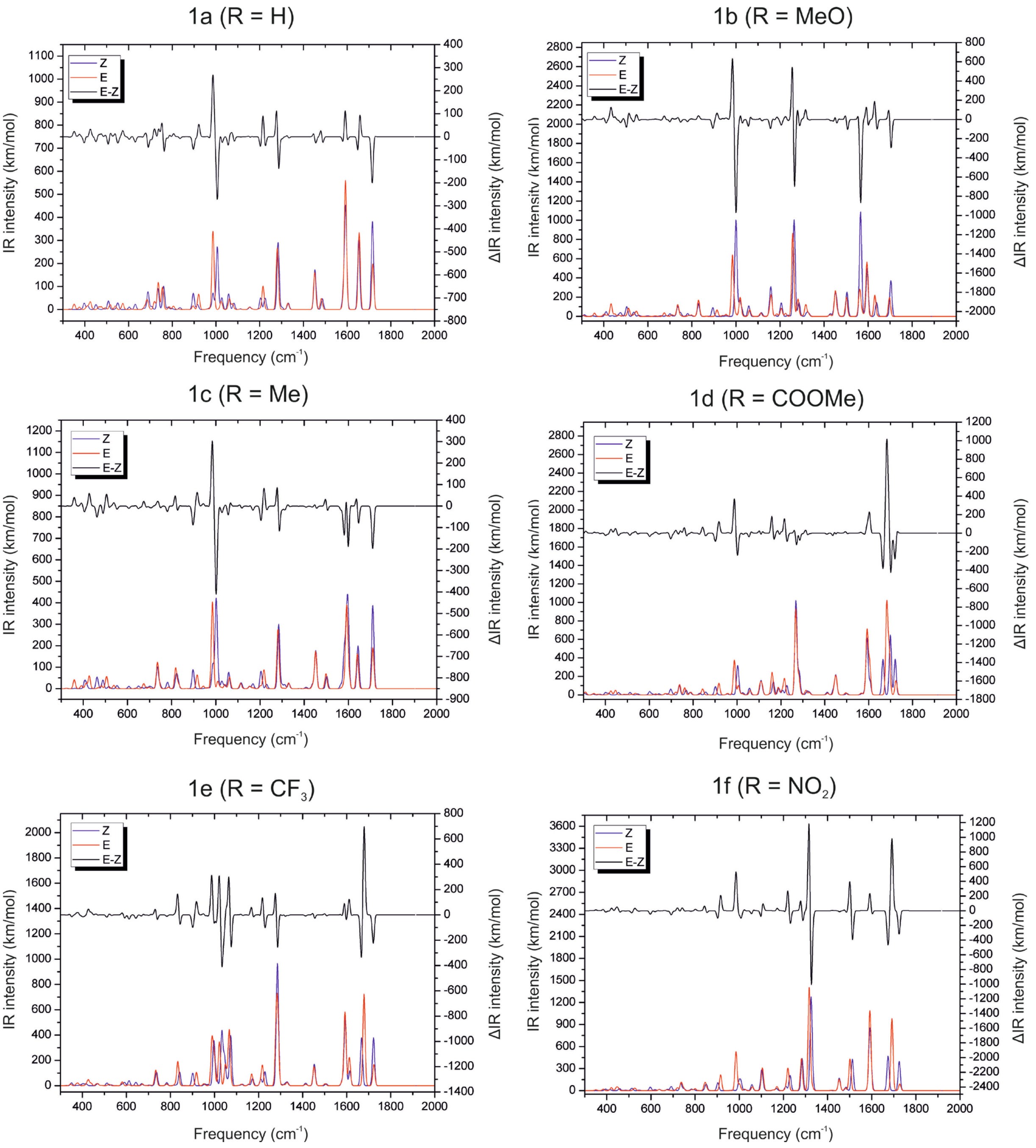

Supplementary Figure 35. *Difference IR spectra (black lines) obtained as the difference of scaled harmonic GS IR spectra (scaling factor f = 0.98) of the E- and Z-isomers of substituted ITIs in acetonitrile calculated with at the SMD-B3LYP/6-31++G(d,p) level.*

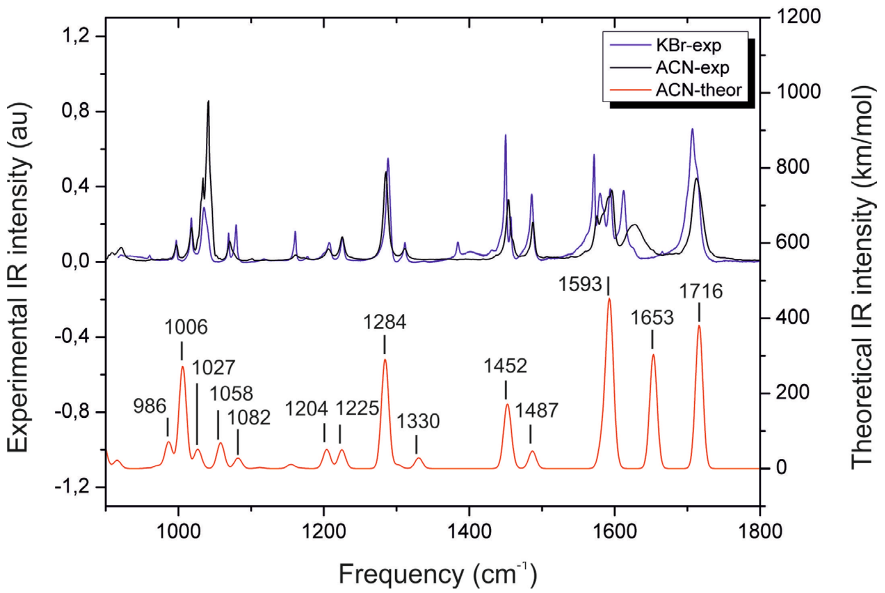

Supplementary Figure 36. *Comparison of simulated SMD-B3LYP/6-31++G(d,p)(solvent=ACN, harmonic approximation, scaling factor f = 0.98) and experimental FTIR spectra measured in solution (ACN) at room temperature (black) and in the solid state (KBr, 184 K) for Z-**1a**. For the band assignment see Supplementary Table 11.*

Supplementary Note 8:

Let us notice that the peak at 1385 cm^{-1} observed in the solid state low temperature spectrum is not present in the experimental spectrum obtained in solution which is apparently the reason of a discrepancy between the experimental and theoretical difference spectrum at 1384/1385 cm^{-1} (Fig. 3C). In addition, DFT fails to reproduce a complex feature in the 1550-1650 cm^{-1} region giving also rise to a discrepancy in the difference spectrum.

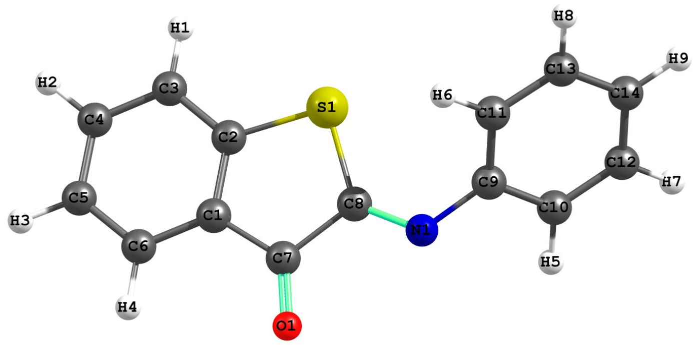

Supplementary Figure 37. *The numbering of atoms used in the band assignment of GS IR spectra of **1a** provided in Supplementary Table 11.*

Supplementary Table 11. Band assignment (ν_{calc}, cm^{-1}) supported by the potential energy distribution (PED) analysis[8] for the simulated SMD-B3LYP/6-31++G(d,p)(solvent=ACN, harmonic approximation, scaling factor f= 0.98) GS IR spectra of the Z and E forms of **1a**. The numbering of atoms is displayed in Supplementary Figure 37.

Z-form ν_{calc}	*E*-form ν_{calc}	Interpretation[a]	PED(%)[a]	Note
986	989[b]	**CCC bend of the benzene C6-ring** + C12-C14/C13-C14/C11-C13/C10-C12 stretchings	**34**+30	weak in *E*-form
1005	986[b]	**thioindigo (TI) C6-ring breathing/C7-C8 stretching**	**60**	intense
1027	1024	**symmetric C3-C4-C5/C7-C8 stretchings** + C3-H1/C4-H2/C6-H4 rocking	**45**+13	
1058	1061	**CCC bend of the TI C6-ring** + S1-C2 stretching	**40**+14	
1082	1076	**C14-H9/C13-H8/C11-H6 rocking** + C11-C13 stretching	**38**+25	
1204	1199	**C1-C7 stretching** + N1-C9 stretching	**25**+22	silent in *E* form
1225	1215	**N1-C9 stretching** + C1-C7 stretching	**23**+14	
1284	1280	**twist mode of the TI C6-ring** + asymmetric C2-C3, C5-C6 stretching	**48**+23	
1331	1329	**CC stretch (kekule) of the TI C6-ring**	**51**	
1452	1451	**C3-H1/C4-H2/C6-H4 rocking** + asymmetric C2-C3, C5-C6 stretching	**47**+20	
1487	1482	**C11-H6/C10-H5/C13-H8 rocking** + CCC bend of the benzene C6-ring	**62**+15	
1593	1593	**C2-C3-C4/C1-C6-C5 asymmetric stretchings**	**44**	
1653	1655	**C8-N1 stretching**	**70**	
1716	1718	**C7-O1 stretching**	**80**	

[a] The dominant motion is written in bold.

[b] The normal modes in the *E*-form are switched with respect to the *Z*-form giving rise to an intensive feature observed in the difference spectrum (see Figs 3C and Supplementary Figure 35 top-left panel).

Supplementary Note 9: NMR shieldings

The calculated averaged isotropic proton NMR shieldings and experimental values obtained in $CDCl_3$ are reported in Supplementary Table 12. For the phenyl ortho/meta protons (blue/orange bullets in Fig. 3A, respectively) the NMR shifts of 7.43/7.55 and 6.97/7.38 ppm for the *Z* and *E* isomers of **1a**, respectively, are observed in experiment (Fig. 3A). Calculated decrease of the δ values (-0.37 and -0.15 ppm) for ortho and meta protons going from *Z* to *E* isomer is in agreement with the experimental observations (-0.46 and -0.19 ppm).

Supplementary Table 12. Average NMR proton shieldings for the phenyl ortho/meta protons in the *Z* and *E* isomers of **1a** [SMD(chcl3)-GIAO-B3LYP/6-31++G(d,p)//SMD(chcl3)-M06-2X/6-31+G(d)] in ppm.

	δ^{exp} *(Z)*	δ^{exp} *(E)*	$\Delta\ \delta^{exp}$	δ^{theo} *(Z)*	δ^{theo} *(E)*	$\Delta\ \delta^{theo}$
C-H_{ortho}	7.43	6.97	-0.46	7.54	7.17	-0.37
C-H_{meta}	7.55	7.38	-0.17	7.83	7.68	-0.15

Supplementary Note 10: Ultrafast kinetics in different solvents

Sub-ps transient absorption spectra have been recorded in methanol, cyclohexane and DMSO. The results are qualitatively similar in all the investigated solvents, with spectral shapes and evolution similar to what is reported in Figure 4 of the main text for methanol. A comparison of selected kinetic traces measured in the three solvents is shown in Supplementary Figure 37.

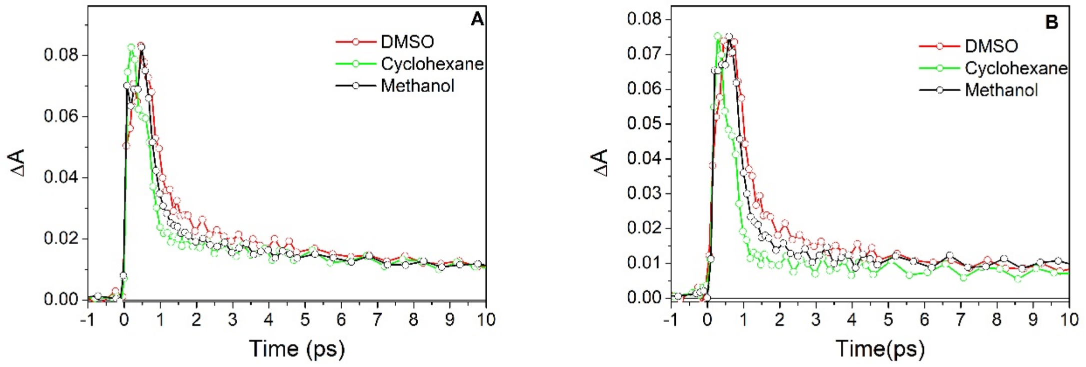

Supplementary Figure 38. *Kinetic traces measured at A) 490 nm and B) 530 nm in cyclohexane, DMSO and methanol.*

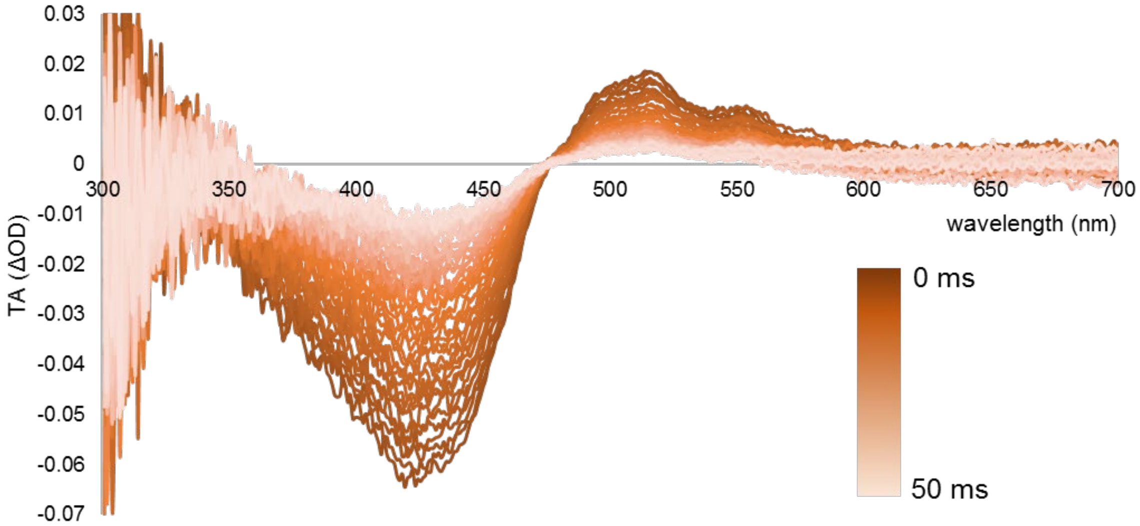

Supplementary Figure 39. *Transient absorption of 400 µM ITI **1a** in MeOH at room temperature. The sample was irradiated with a 430 nm light pulse, upon which the spectrum was recorded in steps of 1 ms increasing delay.*

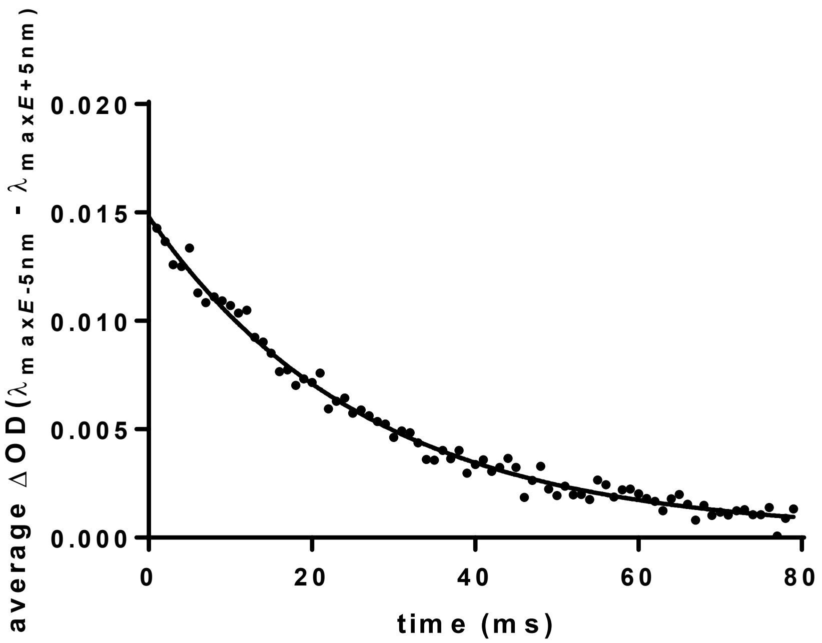

Supplementary Figure 40. *Transient absorption (averaged over λ_{max+5} to λ_{max-5}) over time from Supplementary Figure 39. The calculated half-life of the E isomer is 18.5 ± 1.4 (sd derived from curve fitting).*

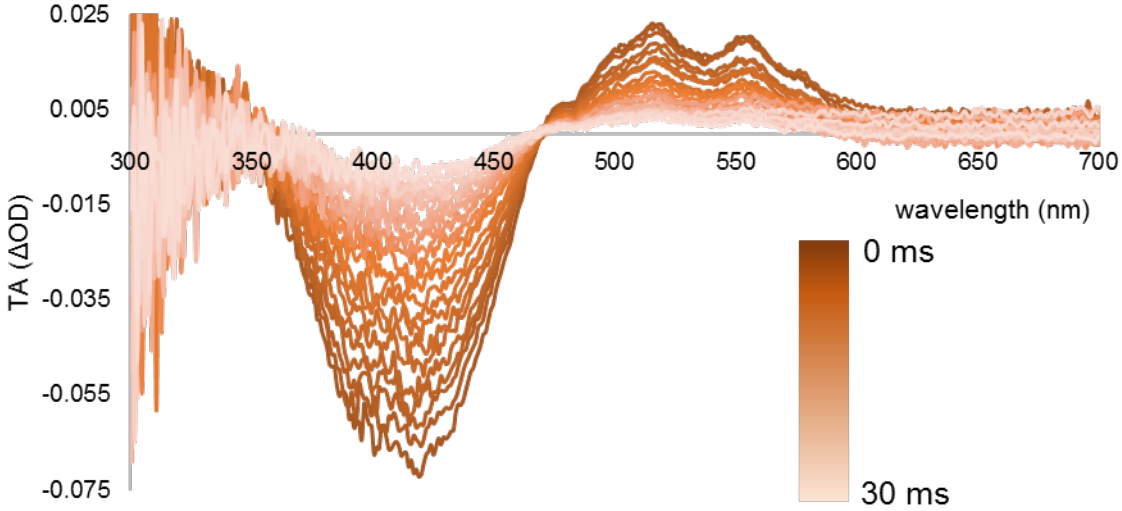

Supplementary Figure 41. *Transient absorption of 400 µM ITI **1a** in cyclohexane at room temperature. The sample was irradiated with a 430 nm light pulse, upon which the spectrum was recorded in steps of 1 ms increasing delay.*

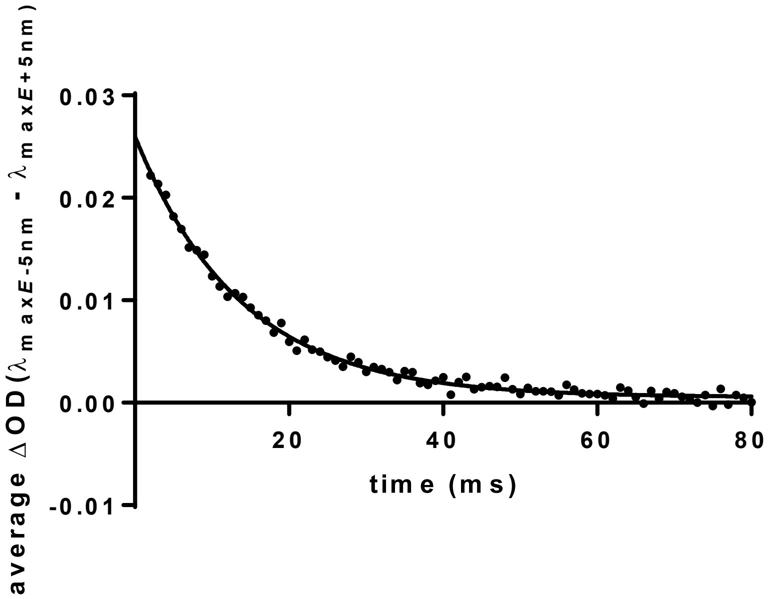

Supplementary Figure 42. *Transient absorption (averaged over λ_{max+5} to λ_{max-5}) over time from Supplementary Figure 41. The calculated half-life of the E isomer is 9.5 ± 0.4 (sd derived from curve fitting).*

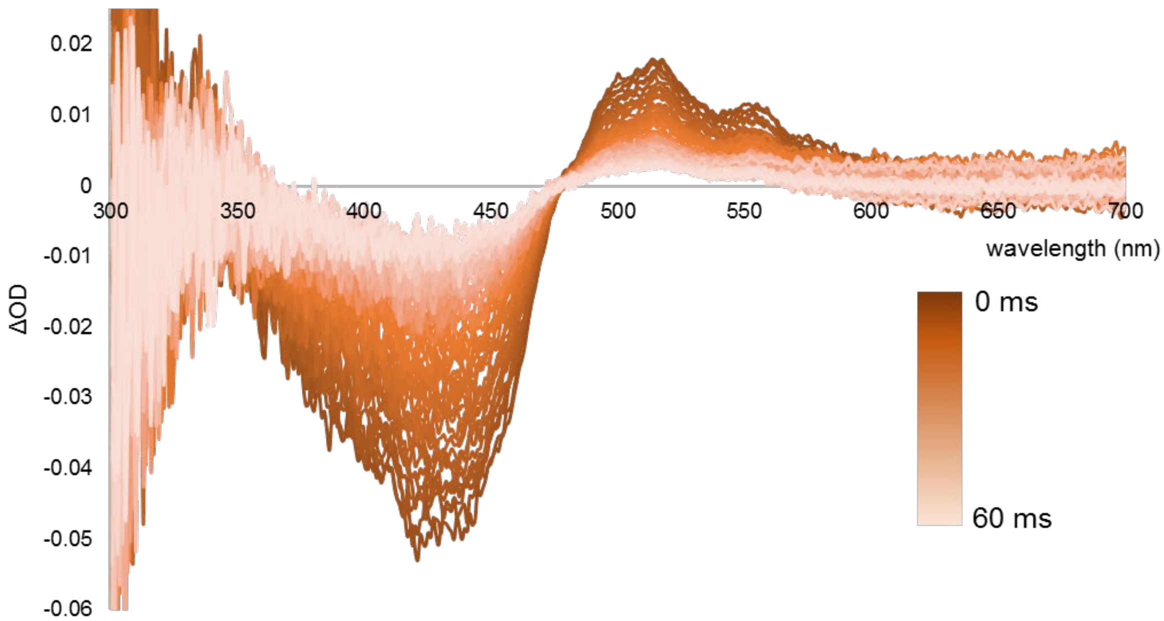

Supplementary Figure 43 *Transient absorption of 400 μM ITI **1a** in DMSO at room temperature. The sample was irradiated with a 430 nm light pulse, upon which the spectrum was recorded in steps of 1 ms increasing delay.*

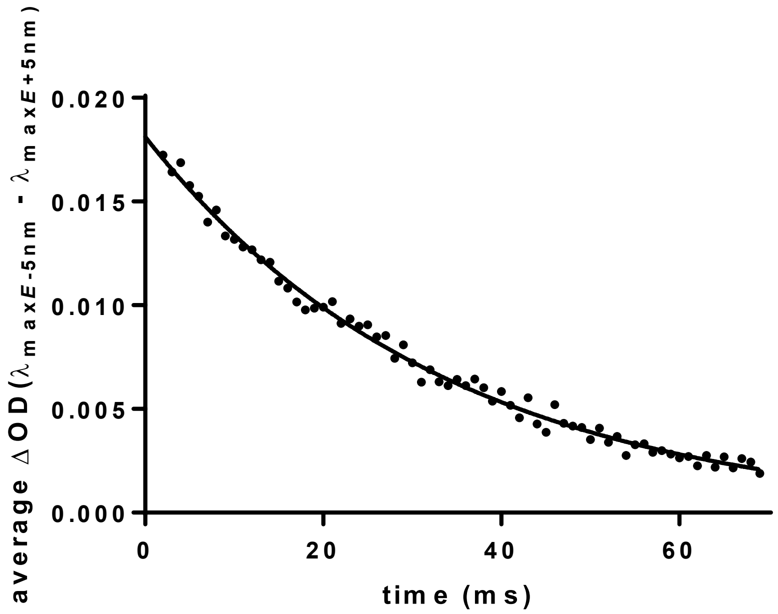

Supplementary Figure 44. *Transient absorption (averaged over λ_{max+5} to λ_{max-5}) over time from Supplementary Figure 43. The calculated half-life of the E isomer is 23.3 ± 2.0 (sd derived from curve fitting).*

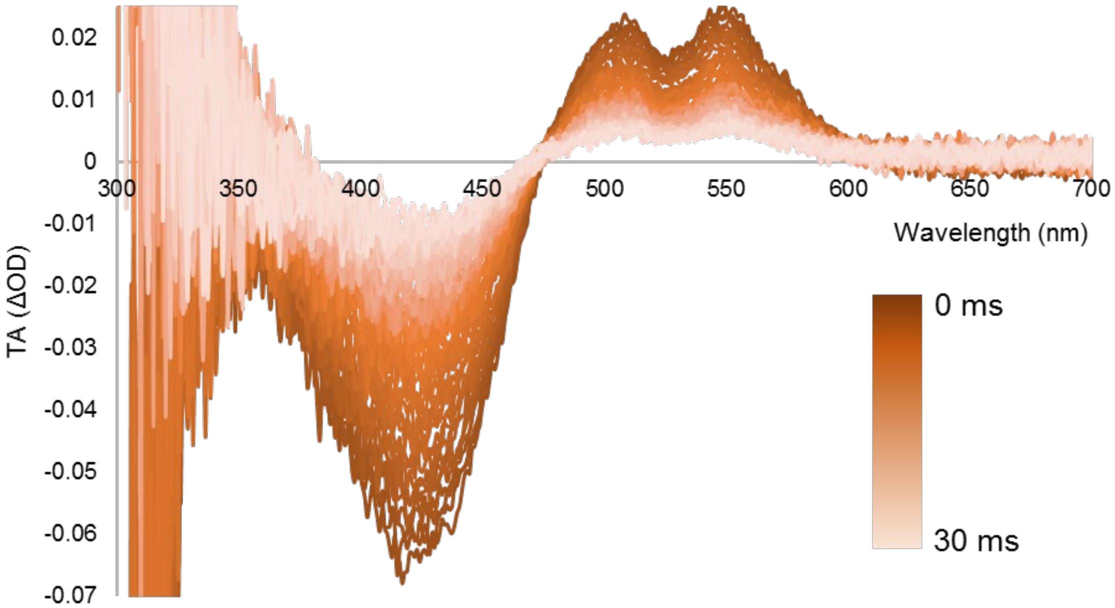

Supplementary Figure 45. *Transient absorption of 400 µM ITI* ***1a*** *in toluene at room temperature. The sample was irradiated with a 430 nm light pulse, upon which the spectrum was recorded in steps of 0.5 ms increasing delay.*

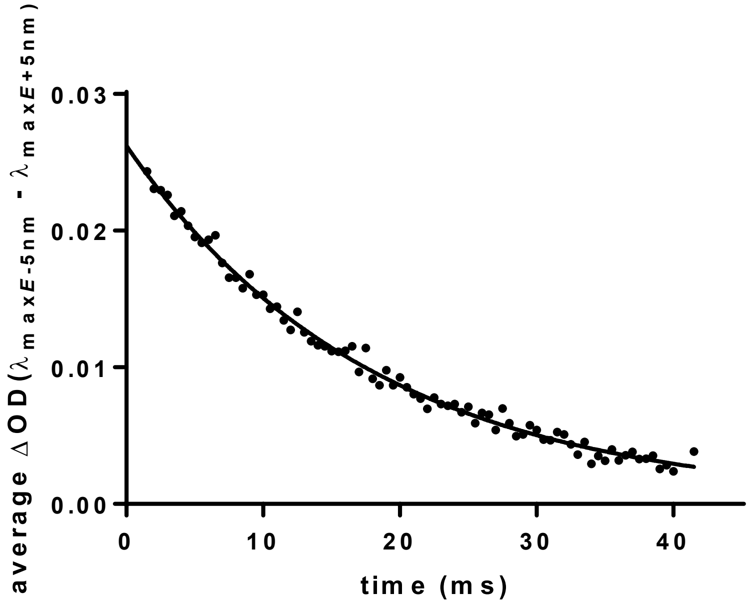

Supplementary Figure 46. *Transient absorption (averaged over λ_{max+5} to λ_{max-5}) over time from Supplementary Figure 45. The calculated half-life of the E isomer is 12.4 ± 0.9 (sd derived from curve fitting).*

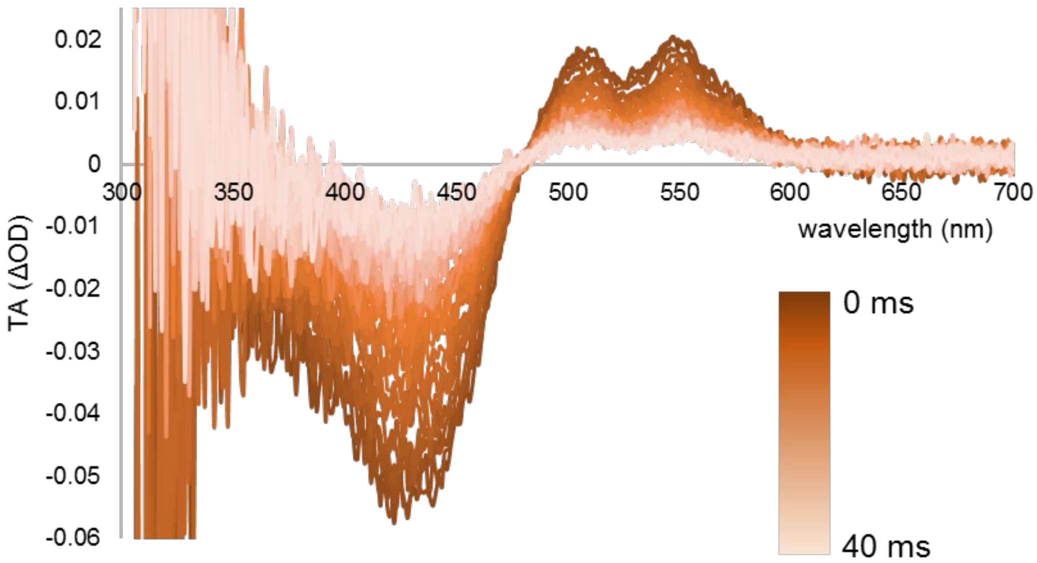

Supplementary Figure 47. *Transient absorption of 400 µM ITI **1a** in Chloroform at room temperature. The sample was irradiated with a 430 nm light pulse, upon which the spectrum was recorded in steps of 1 ms increasing delay.*

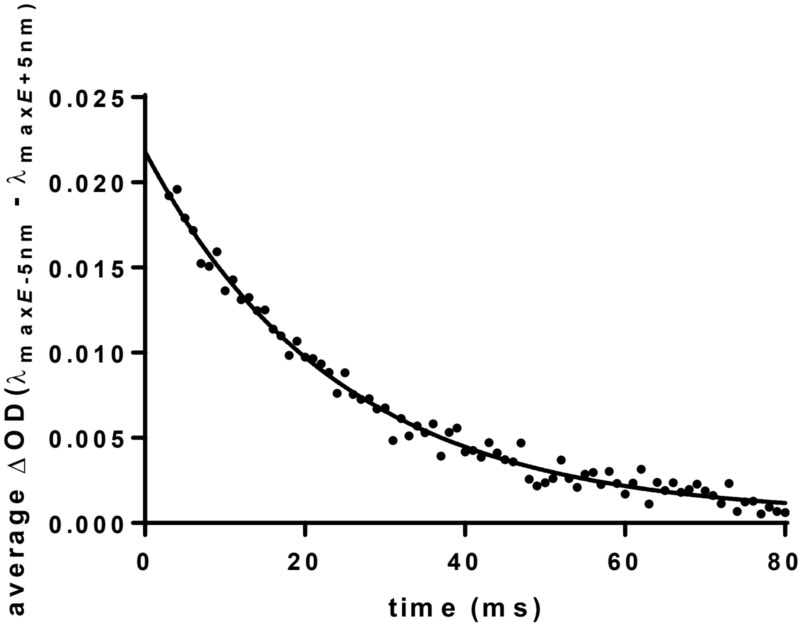

Supplementary Figure 48. *Transient absorption (averaged over λ_{max+5} to λ_{max-5}) over time from Supplementary Figure 47. The calculated half-life of the E isomer is 16.9 ± 1.2 (sd derived from curve fitting).*

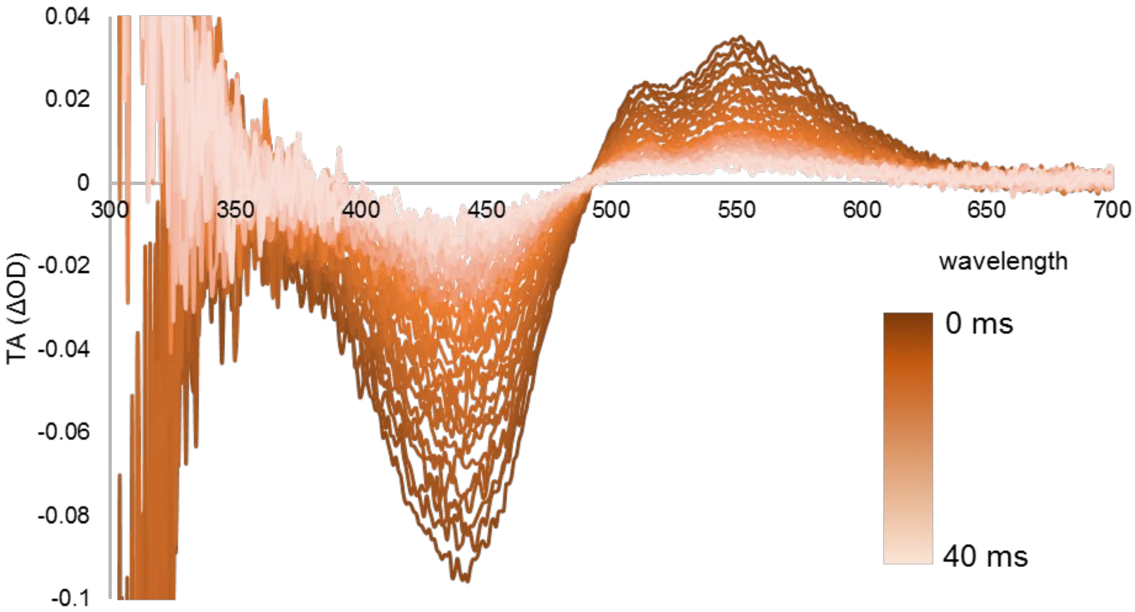

Supplementary Figure 49. *Transient absorption of 120 µM ITI* ***1b*** *(p-MeO) in MeOH at room temperature. The sample was irradiated with a 430 nm light pulse, upon which the spectrum was recorded in steps of 1 ms increasing delay.*

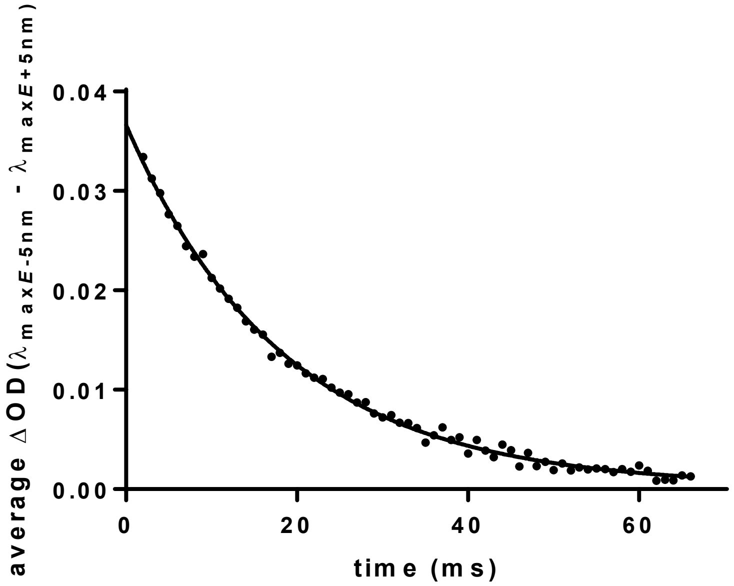

Supplementary Figure 50. *Transient absorption (averaged over λ_{max+5} to λ_{max-5}) over time from Supplementary Figure 49. The calculated half-life of the E isomer is 12.7 ± 0.5 (sd derived from curve fitting).*

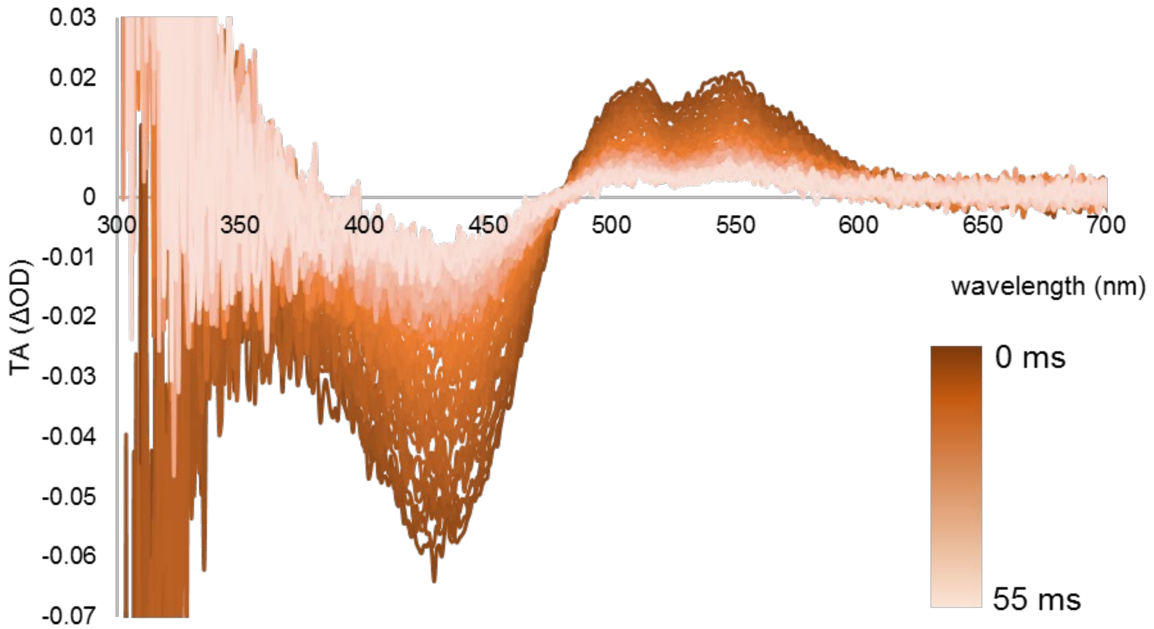

Supplementary Figure 51. *Transient absorption of 200 µM ITI **1c** (p-Me) in MeOH at room temperature. The sample was irradiated with a 430 nm light pulse, upon which the spectrum was recorded in steps of 1 ms increasing delay.*

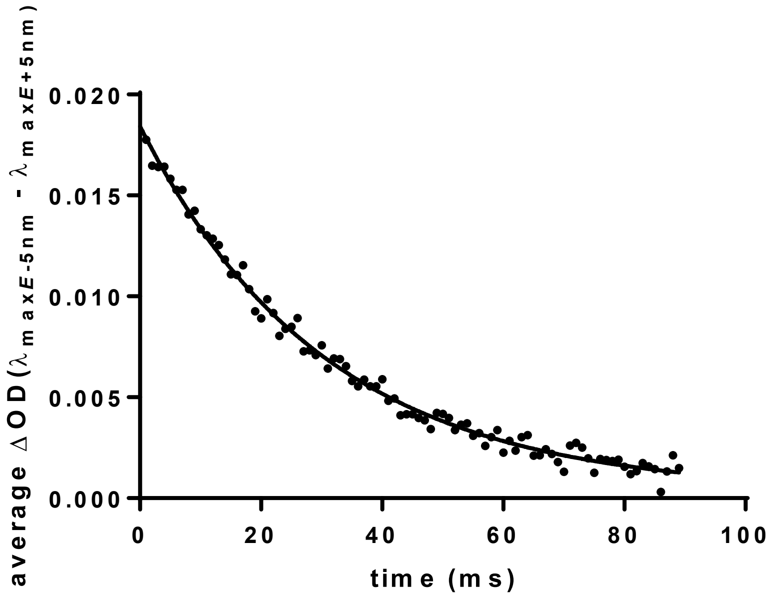

Supplementary Figure 52. *Transient absorption (averaged over λ_{max+5} to λ_{max-5}) over time from Supplementary Figure 51. The calculated half-life of the E isomer is 21.1 ± 1.2 (sd derived from curve fitting).*

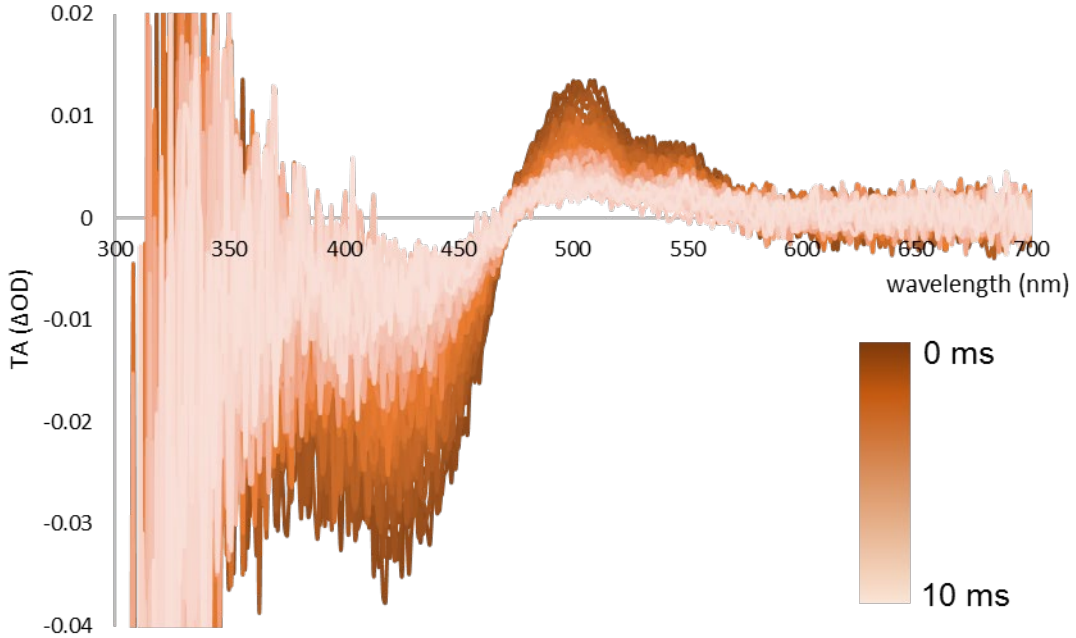

Supplementary Figure 53. *Transient absorption of 750 µM ITI* ***1d*** *(p-COOMe) in MeOH at room temperature. The sample was irradiated with a 430 nm light pulse, upon which the spectrum was recorded in steps of 0.2 ms increasing delay.*

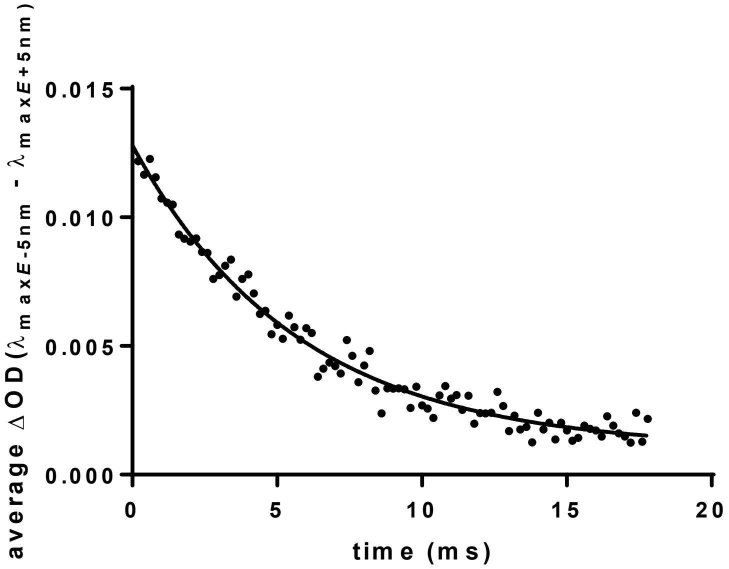

Supplementary Figure 54. *Transient absorption (averaged over λ_{max+5} to λ_{max-5}) over time from Supplementary Figure 53. The calculated half-life of the E isomer is 4.0 ± 0.3 (sd derived from curve fitting).*

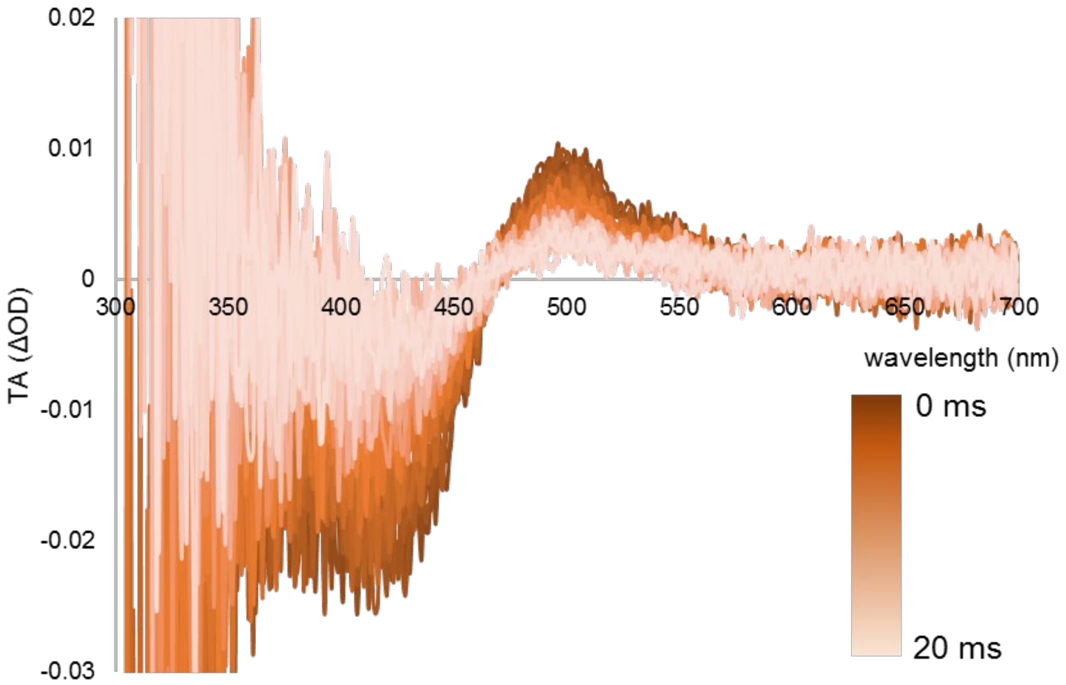

Supplementary Figure 55. *Transient absorption of 750 µM ITI **1e** (p-CF_3) in MeOH at room temperature. The sample was irradiated with a 430 nm light pulse, upon which the spectrum was recorded in steps of 0.5 ms increasing delay.*

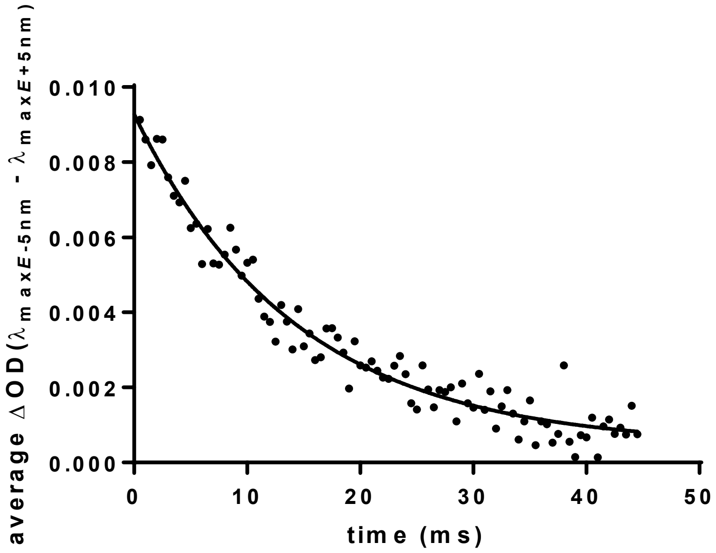

Supplementary Figure 56. *Transient absorption (averaged over λ_{max+5} to λ_{max-5}) over time from Supplementary Figure 55. The calculated half-life of the E isomer is 9.9 ± 1.0 (sd derived from curve fitting).*

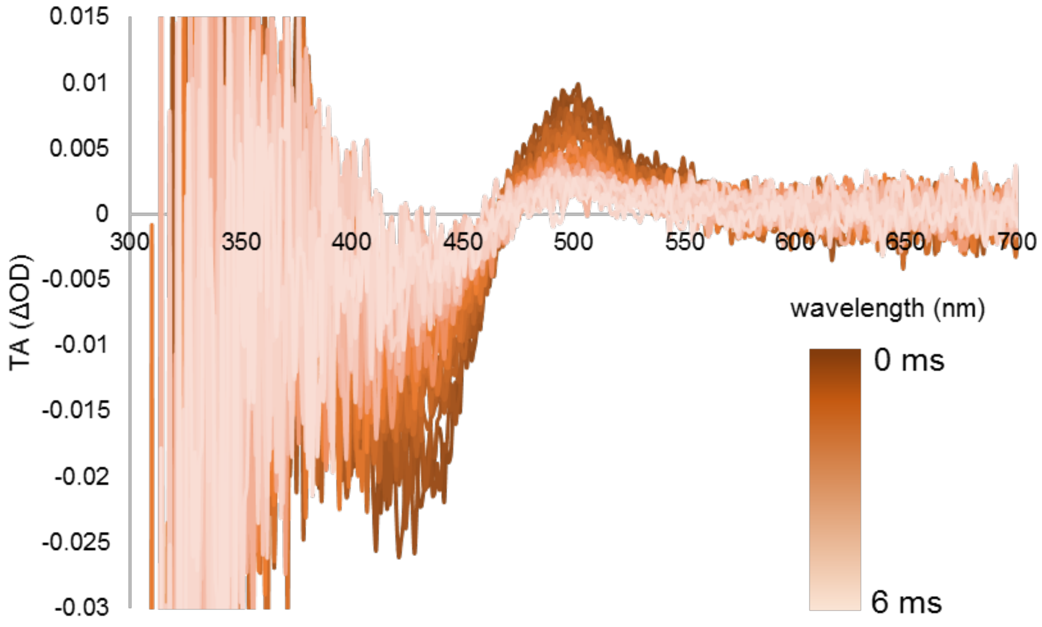

Supplementary Figure 57. *Transient absorption of 600 µM ITI* ***1f*** *(p-NO_2) in MeOH at room temperature. The sample was irradiated with a 430 nm light pulse, upon which the spectrum was recorded in steps of 0.2 ms increasing delay.*

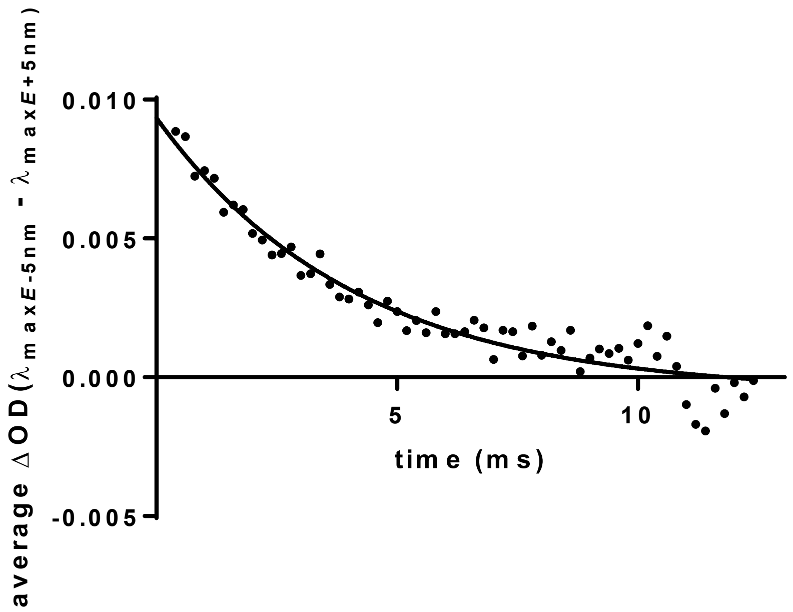

Supplementary Figure 58. *Transient absorption (averaged over λ_{max+5} to λ_{max-5}) over time from Supplementary Figure 57. The calculated half-life of the E isomer is 2.8 ± 0.5 (sd derived from curve fitting).*

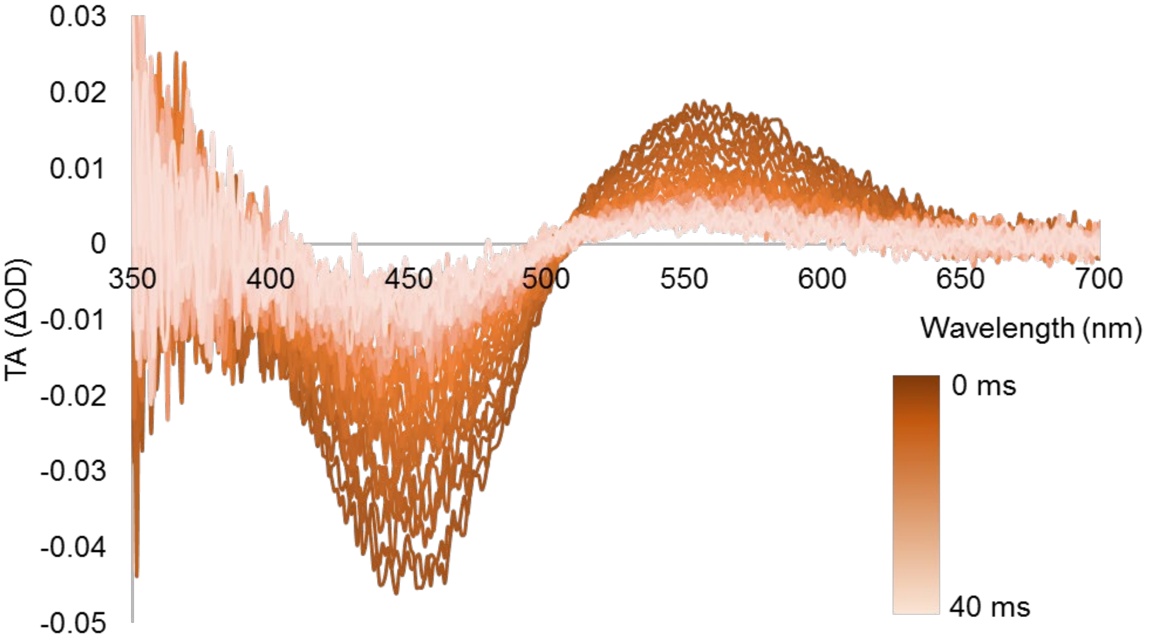

Supplementary Figure 59. *Transient absorption of 120 µM ITI **1b** (p-MeO) in PBS (pH 7.4, 6.67% DMSO) at room temperature. The sample was irradiated with a 430 nm light pulse, upon which the spectrum was recorded in steps of 1 ms increasing delay.*

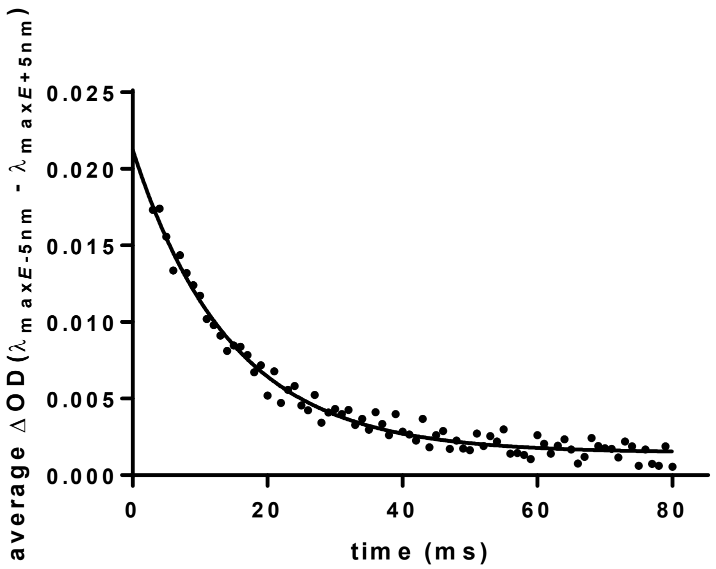

Supplementary Figure 60. *Transient absorption (averaged over λ_{max+5} to λ_{max-5}) over time from Supplementary Figure 59. S3.22. The calculated half-life of the E isomer is 10.0 ± 0.8 (sd derived from curve fitting).*

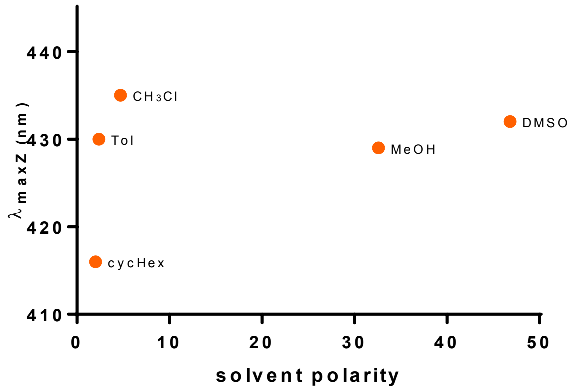

Supplementary Figure 61. *The correlation between the solvent polarity and* $\lambda_{max,Z}$.

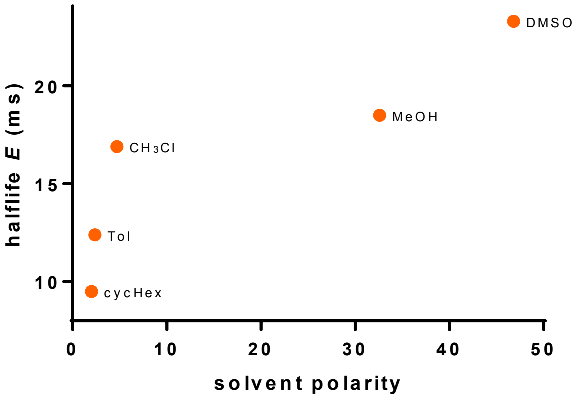

Supplementary Figure 62. *The correlation between solvent polarity and half-life of the E isomer.*

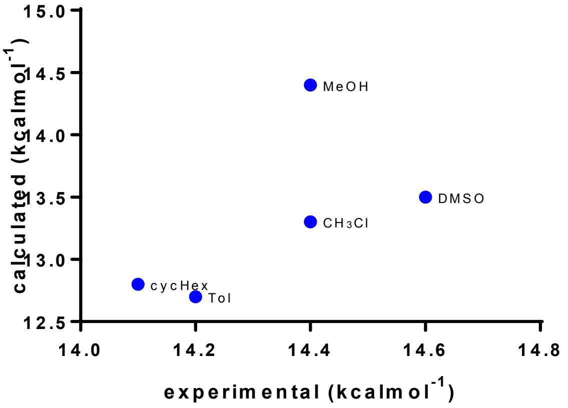

Supplementary Figure 63. *The correlation between calculated and experimental* $\Delta G^{\ddagger}_{E\text{-}Z}$

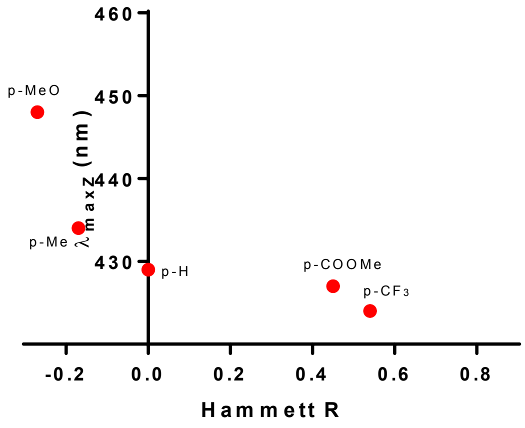

Supplementary Figure 64. *The correlation between Hammett value R and $\lambda_{max,Z}$*

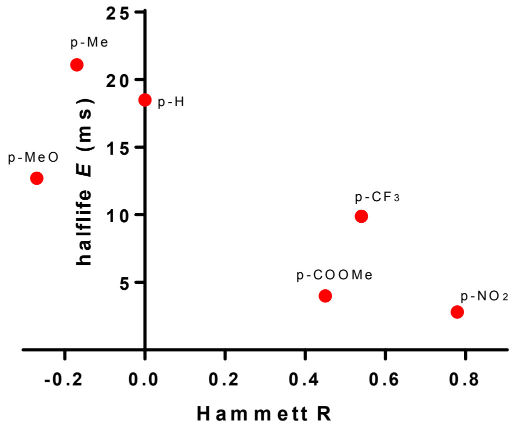

Supplementary Figure 65. *The correlation between Hammett value R and half-life of the E isomer.*

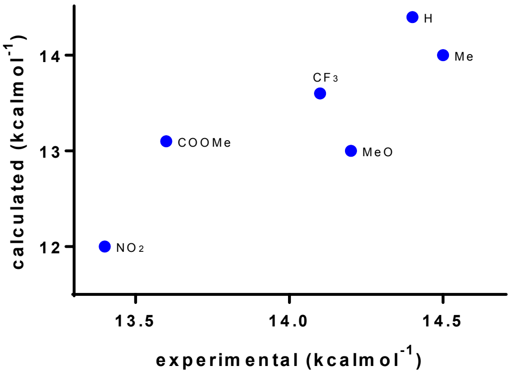

Supplementary Figure 66. *The correlation between calculated and experimental $\Delta G^{\ddagger}_{E\text{-}Z}$*

Supplementary Note 11: PSS at -60 °C.

All ITIs were dissolved at the solubility limit in CD_3OD and subsequently diluted 2 times with CD_3OD. At -60°C, the ITIs were irradiated with 455 nm light upon the photo-stationairy state PSS was reached. Continuous irradiation of *p*-NO_2-ITI **1f** resulted in degradation, all other ITIs did not show any sign of degradation.

Supplementary Table 13. *In NMR irradiation using 455 nm light. All experiments at -60°C in CD_3OD.*

Compound	PSS (455 nm)	Degradation observed?	$t_{1/2}$ (min)
p-H-ITI **1a**	65%	no	6.8 ± 0.5
p-MeO-ITI **1b**	83%	no	4.0 ± 0.3
p-Me-ITI **1c**	73%	no	10.0 ± 0.6
p-COOMe-ITI **1d**	17%	no	0.8 ± 0.1
p-CF_3-ITI **1e**	36%	no	2.6 ± 0.3
p-NO_2-ITI **1f**	-	yes	-

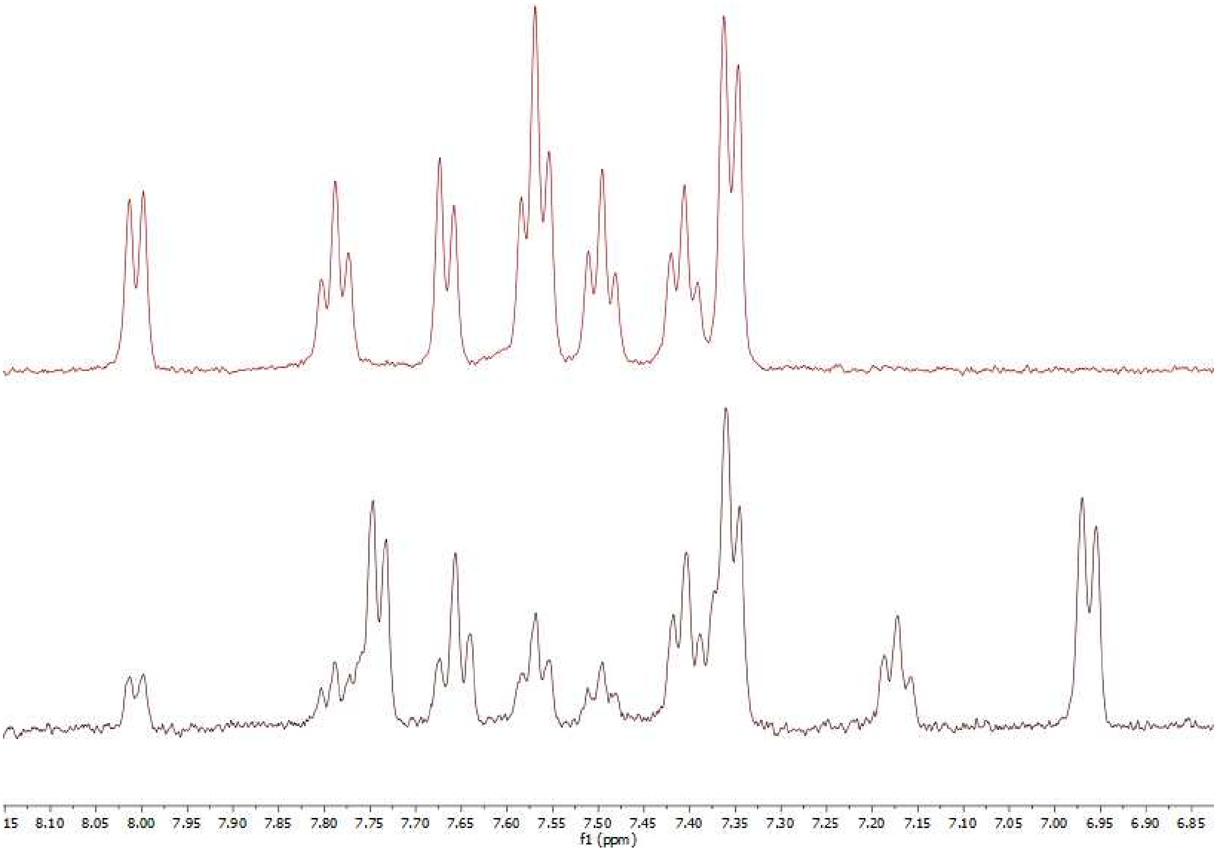

Supplementary Figure 67. *p-H-ITI* ***1a*** *in CD_3OD at -60 °C. Top: Thermal spectrum. Bottom spectrum: PSS at 455 nm irradiation.*

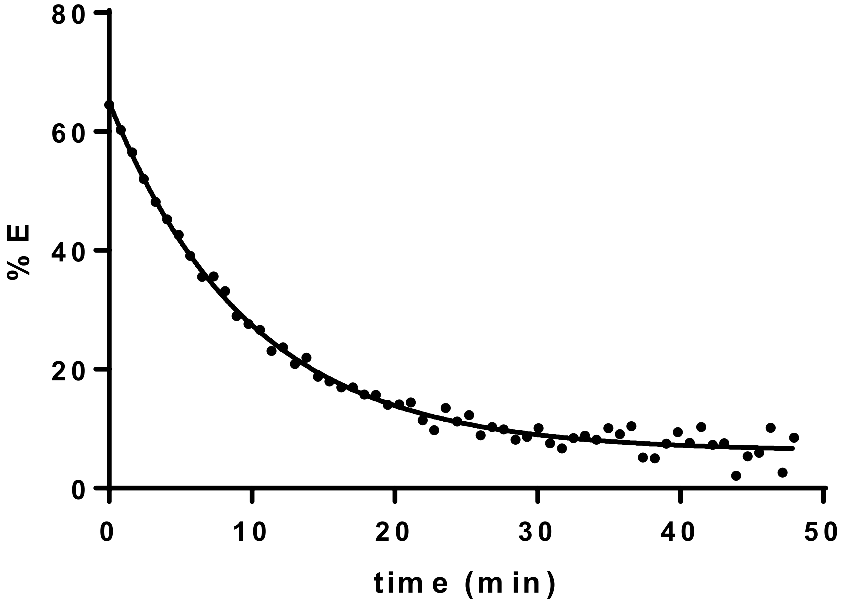

Supplementary Figure 68. *Thermal relaxation of p-H-ITI* ***1a*** *at -60 °C in CD_3OD.*

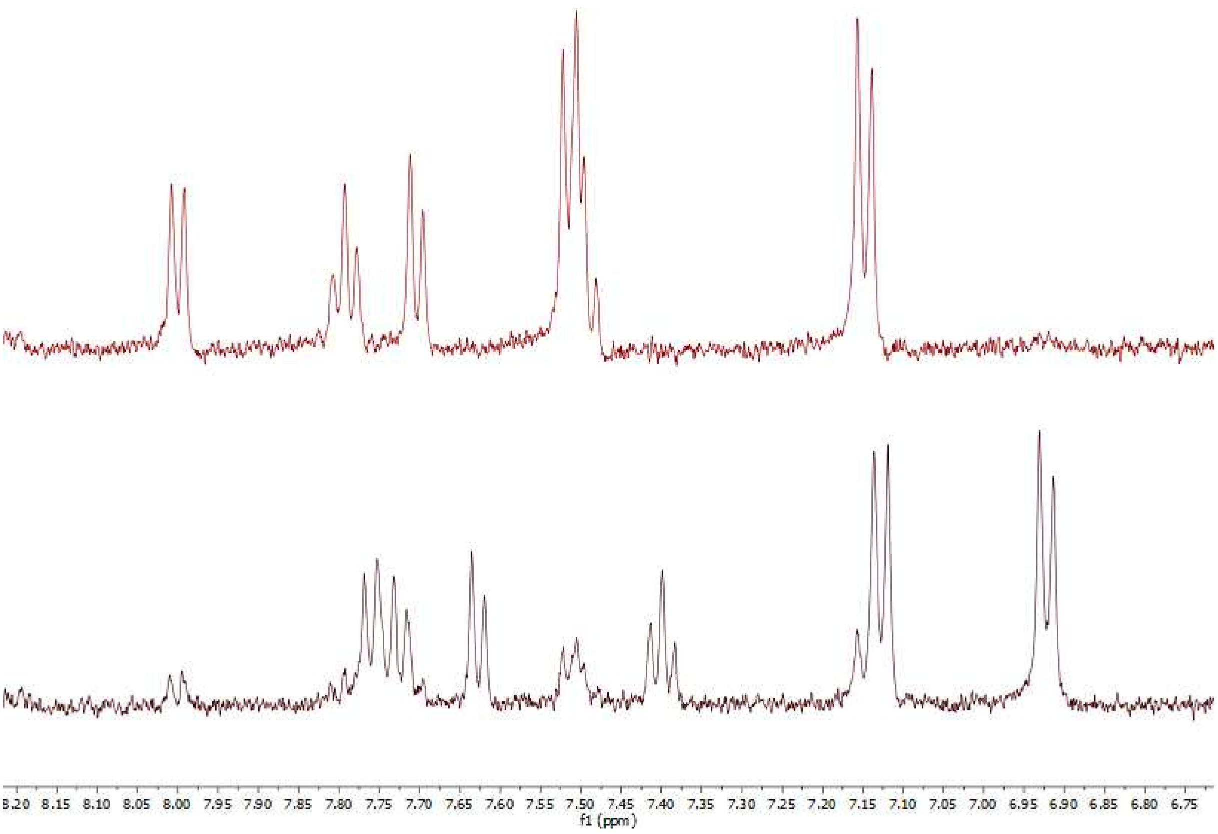

Supplementary Figure 69. *p-MeO-ITI* ***1b*** *in CD_3OD at -60 °C. Top: Thermal spectrum. Bottom spectrum: PSS at 455 nm irradiation.*

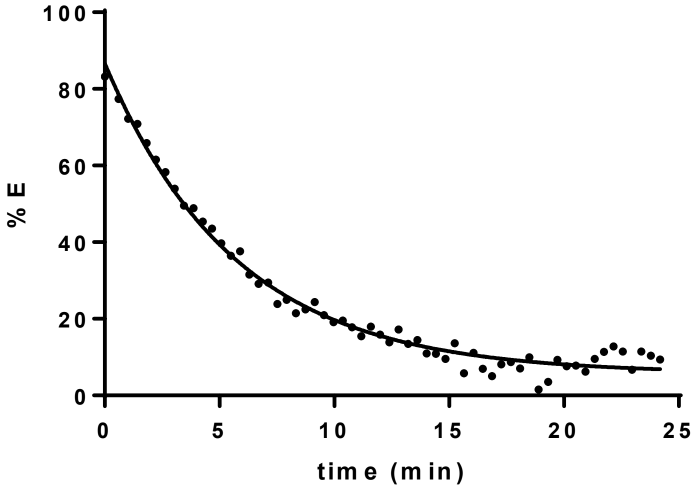

Supplementary Figure 70. *Thermal relaxation of p-MeO-ITI* ***1b*** *at -60 °C in CD_3OD.*

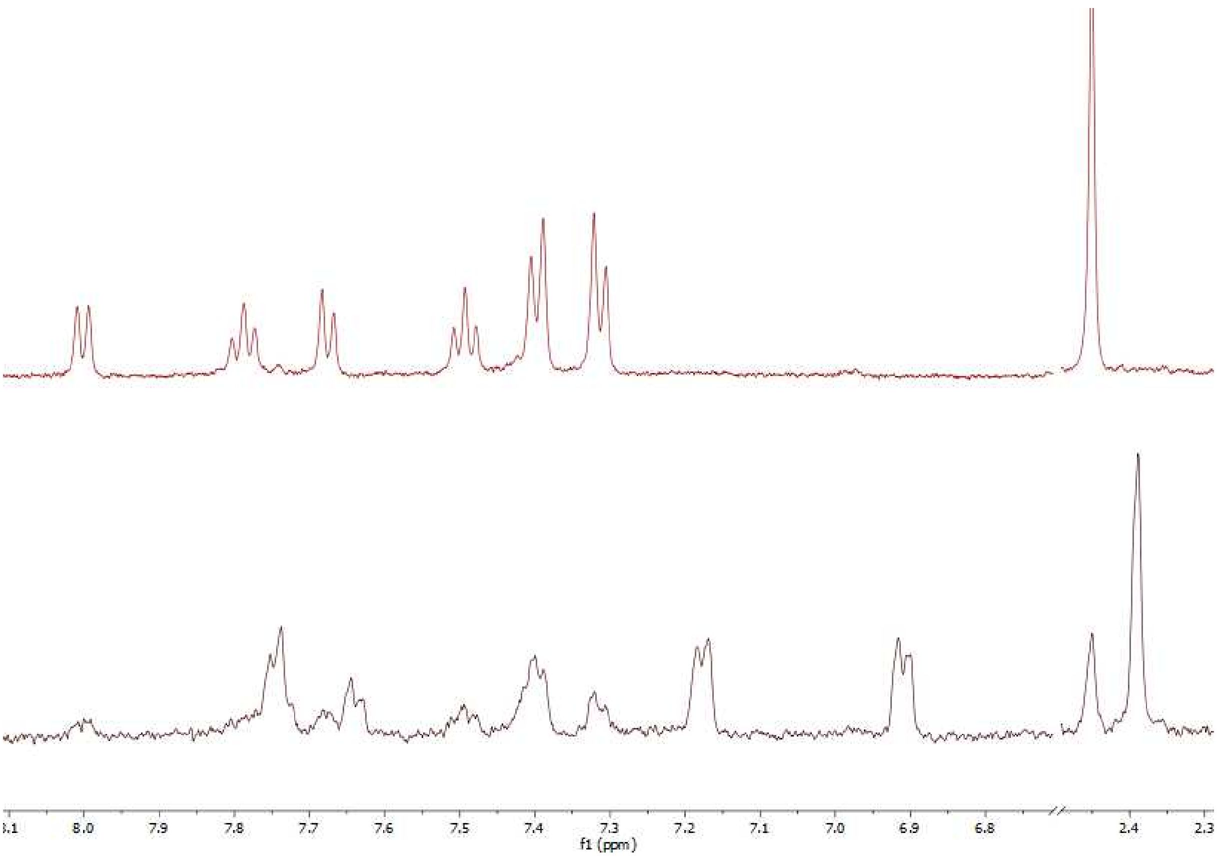

Supplementary Figure 71. *p-Me-ITI **1c** in CD_3OD at -60 °C. Top: Thermal spectrum. Bottom spectrum: PSS at 455 nm irradiation.*

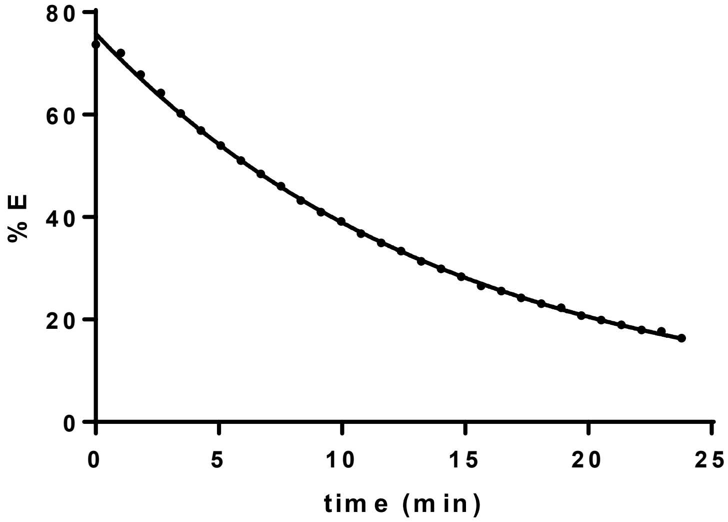

Supplementary Figure 72. *Thermal relaxation of p-Me-ITI **1c** at -60 °C in CD_3OD.*

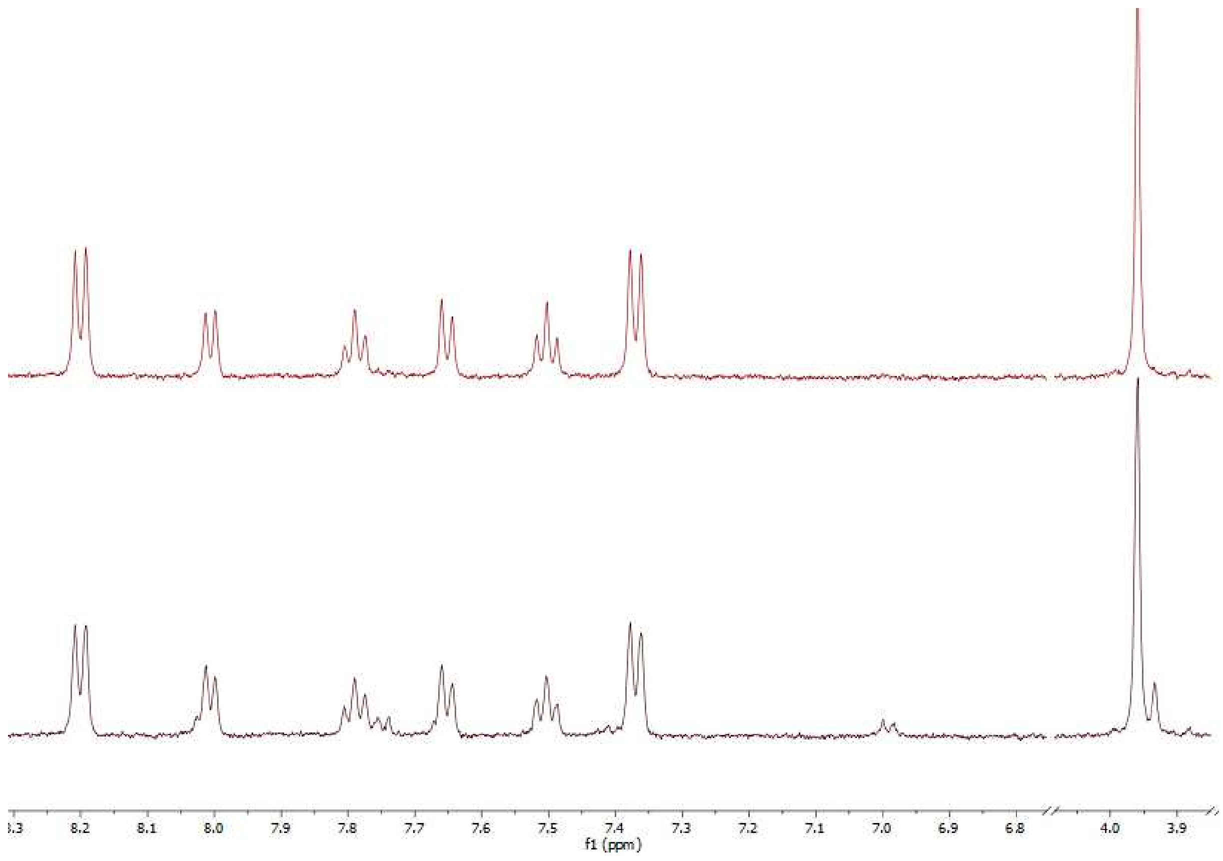

Supplementary Figure 73. *p-COOMe-ITI* ***1d*** *in* CD_3OD *at -60 °C. Top: Thermal spectrum. Bottom spectrum: PSS at 455 nm irradiation.*

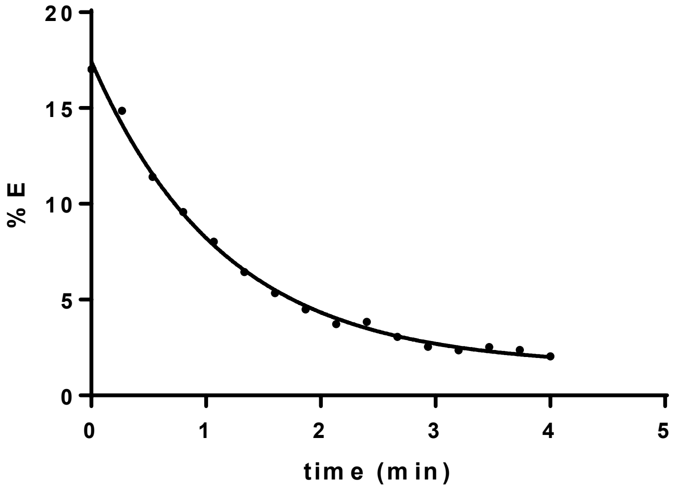

Supplementary Figure 74. *Thermal relaxation of p-COOMe-ITI* ***1d*** *at -60 °C in* CD_3OD*.*

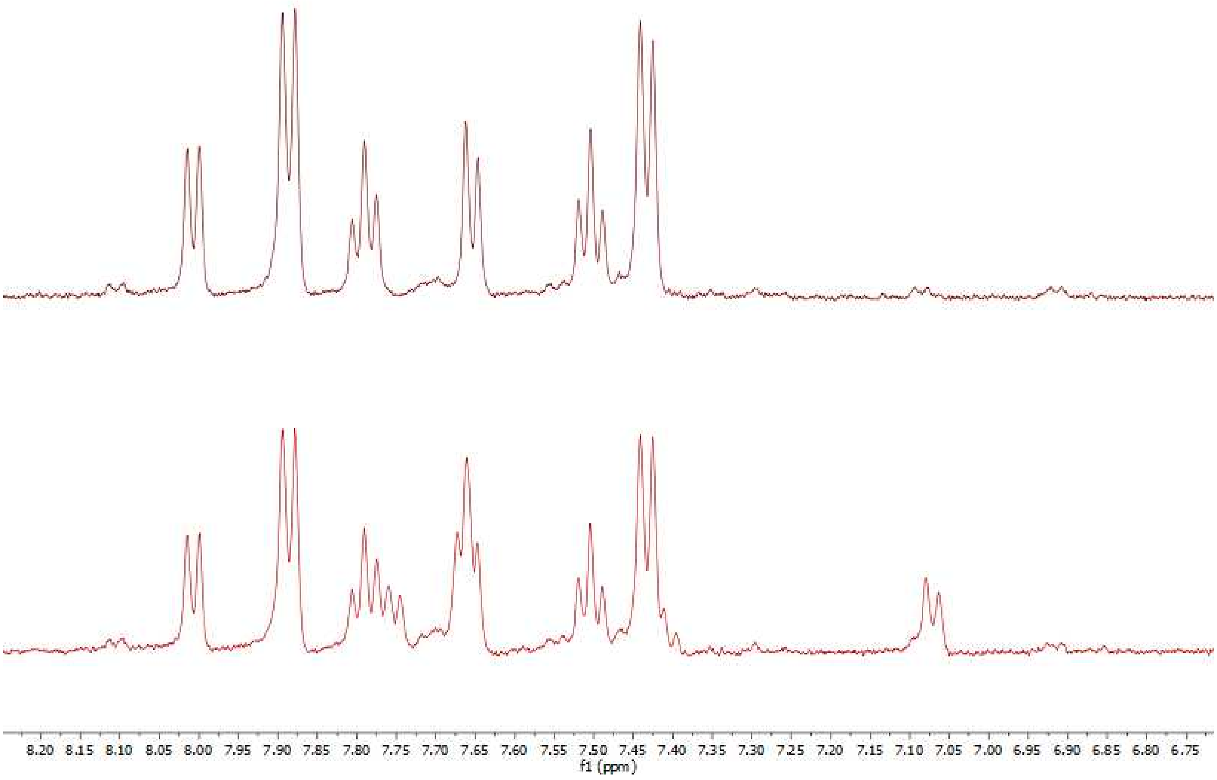

Supplementary Figure 75. *p-CF_3-ITI* ***1e*** *in CD_3OD at -60°C. Top: Thermal spectrum. Bottom spectrum: PSS at 455 nm irradiation.*

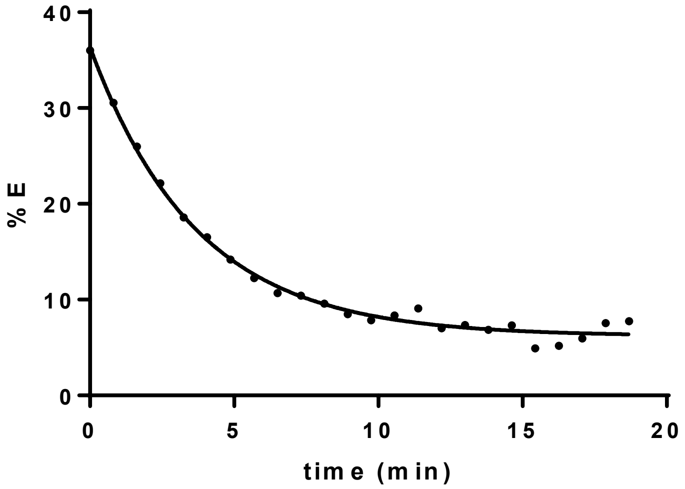

Supplementary Figure 76. *Thermal relaxation of p-CF_3-ITI* ***1d*** *at -60°C in CD_3OD.*

Supplementary Note 12:

The temperature of the NMR measurement in CD_3OD was determined by signals of CH_3 and OH of MeOH.[9]

Formula 1: $T\ (K) = 409 - 36.54\ \delta(CH_3 - OH) - 21.85\ \delta(CH_3 - OH)^2$

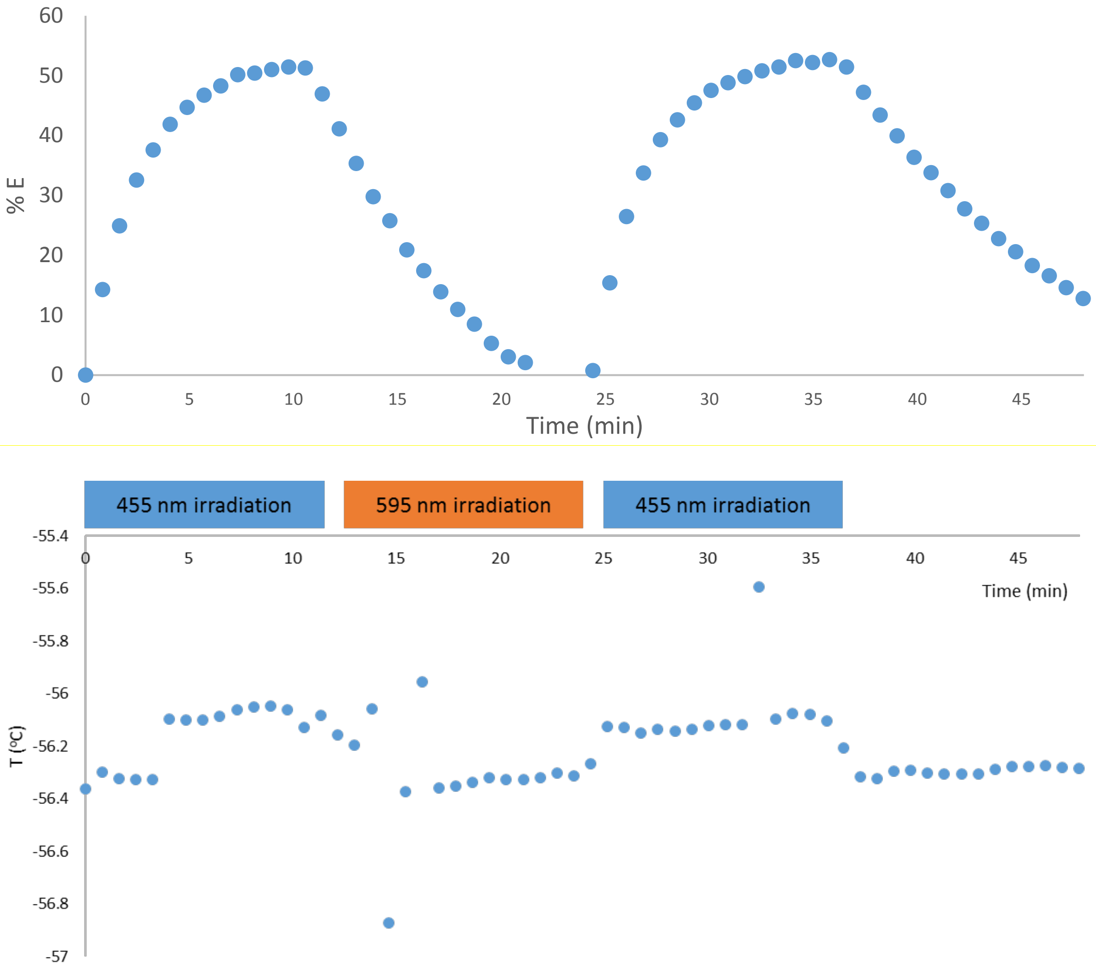

Supplementary Figure 77. *Temperature profile during in situ NMR irradiation experiments with 455 nm and 595 nm irradiation.*

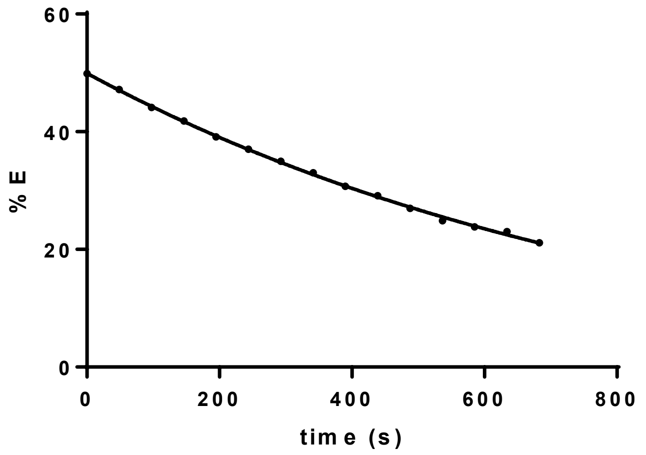

Supplementary Figure 78. *Thermal relaxation of p-H-ITI **1a** in CD_3OD at -58.6 °C*

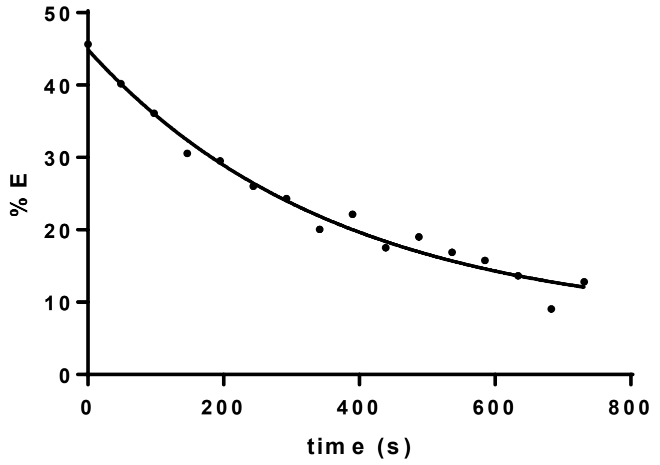

Supplementary Figure 79. *Thermal relaxation of p-H-ITI **1a** in CD_3OD at -52.8 °C*

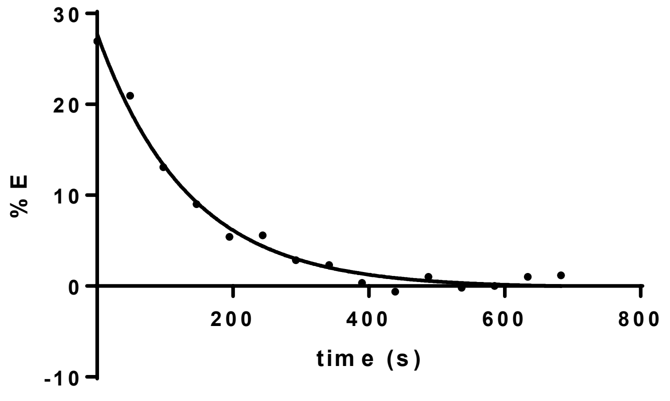

Supplementary Figure 80. *Thermal relaxation of p-H-ITI **1a** in CD_3OD at -47.4 °C*

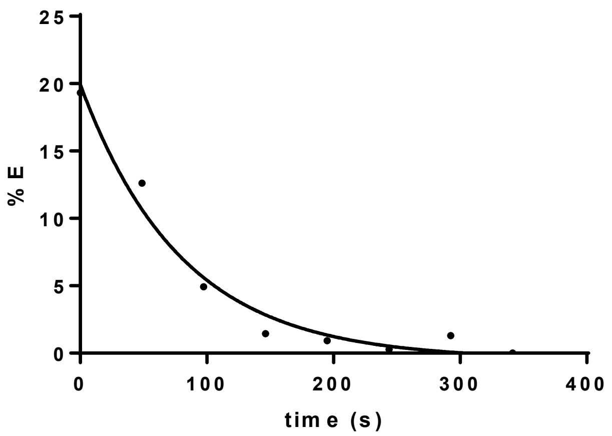

Supplementary Figure 81. *Thermal relaxation of p-H-ITI* ***1a*** *in CD_3OD at -41.6 °C*

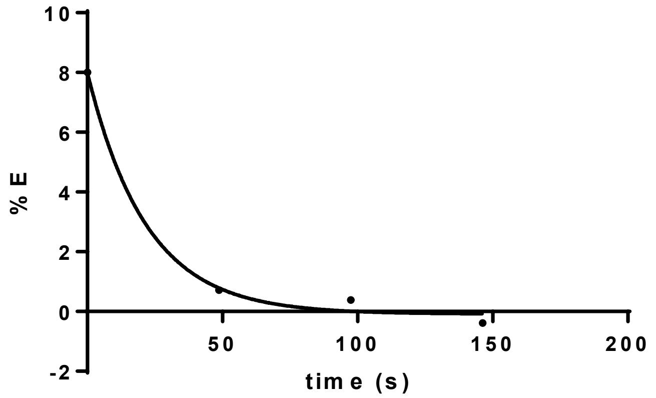

Supplementary Figure 82. *Thermal relaxation of p-H-ITI* ***1a*** *in CD_3OD at -35.9 °C*

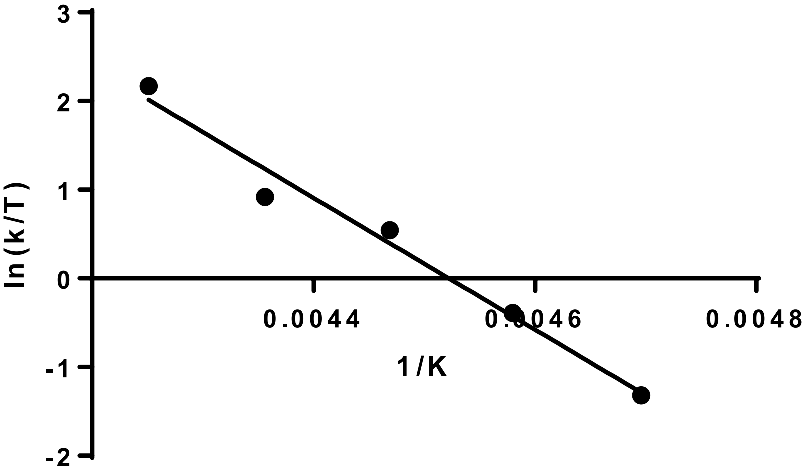

Supplementary Figure 83. *Eyring plot of p-H-ITI* ***1a*** *in CD_3OD.*

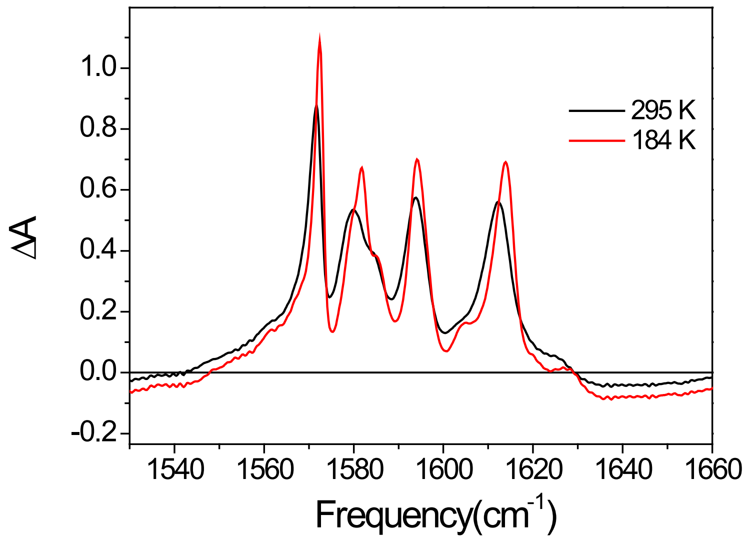

Supplementary Figure 84 *Portion of ITI-**a** FTIR spectrum before irradiation measured at 295 K and 184 K.*

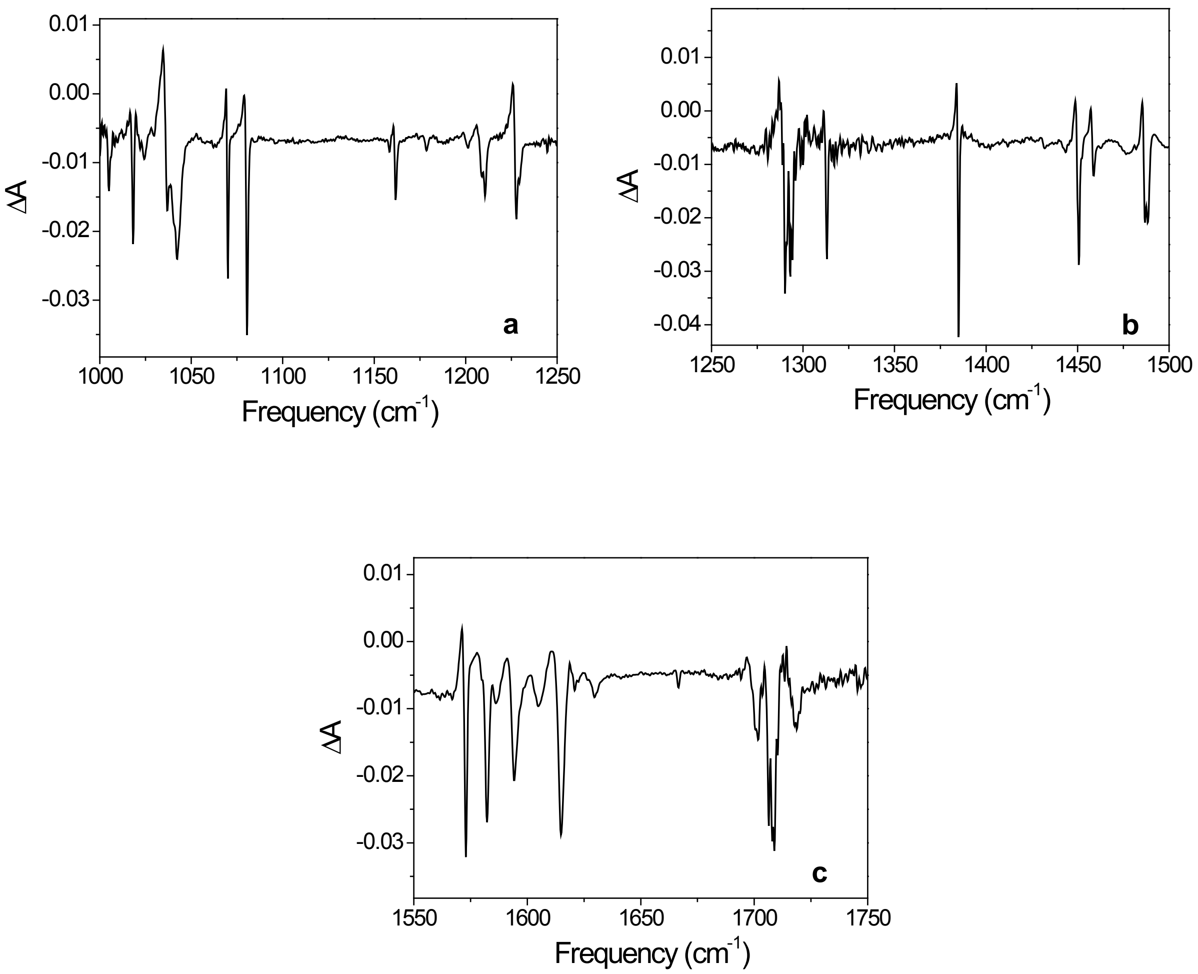

Supplementary Figure 85. *Light minus dark FTIR difference spectrum of ITI-**a**. The spectrum is shown in three separate panels for a better visualization. a) 1000-1250 cm^{-1} region; b) 1250-1500 cm^{-1} region a) 1550-1750 cm^{-1} region.*

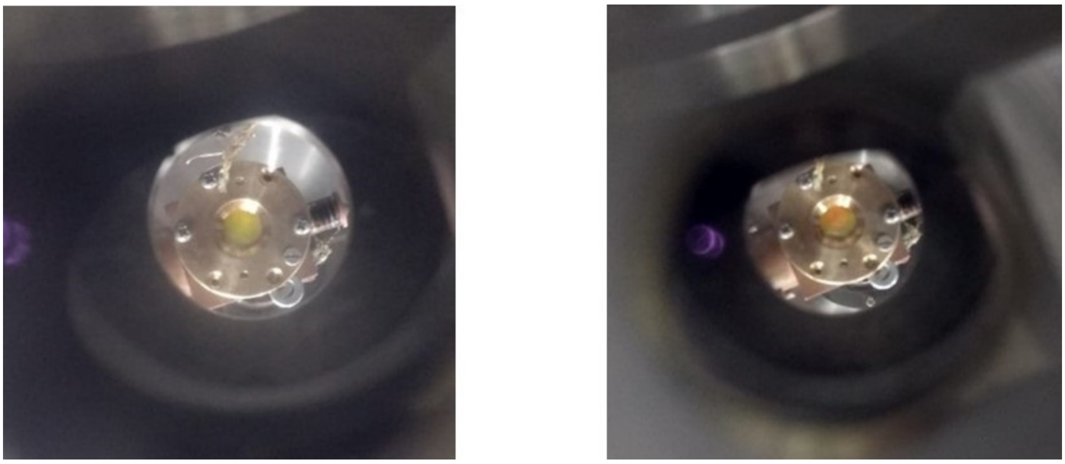

Supplementary Figure 86. *Photographs of an ITI-**a** sample in Kbr contained in the FTIR cell at 180 K in the absence (left) and under of 405 nm irradiation (right). A visible change in color is appreciable in the irradiated spot.*

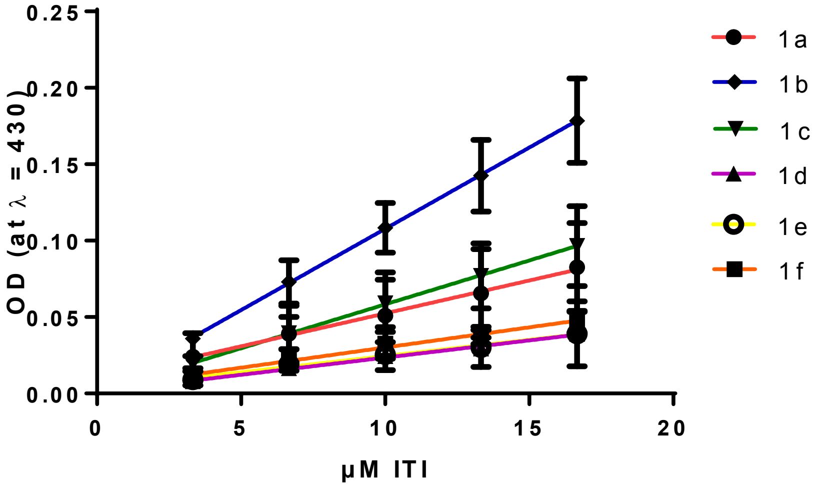

Supplementary Figure 87. *OD at absorption maximum of ITIs **1a-f** at different concentrations in MeOH. Experiment performed in duplo from two independent prepared stock solutions.*

Supplementary Note 13:

An estimate quantum yield was determined by calculating the number of molecules excited, by the observed bleach upon irradiation using the Lambert-Beer law, corrected by the probability of absorption. Herein was assumed that the *E* isomer does not absorb in this region, hence if the *E* isomer absorbs at 430 nm, these numbers are an under estimation.

Supplementary Table 14. *Quantum yield calculations.*

	R	ε_Z (M^{-1} cm^{-1}) (430 nm)	OD in 1 cm cell @430 nm	Concentration (mM) calculated from OD=εcd	Calculated Absorption @λ_{max} = maximum bleach possible	Maximum bleach observed @λ_{max}	$\varphi_{Z\text{->}E}$ (%)[(a)]
**1a**	H	4300	1.64	0.381	0.99	-0.061	6.2
**1b**	MeO	11000	1.33	0.121	2.12	-0.095	4.5
**1c**	Me	5700	1.21	0.212	1.01	-0.055	5.4
**1d**	COOMe	2300	1.69	0.735	0.54	-0.034	6.3
**1e**	CF_3	2100	1.59	0.757	0.47	-0.023	4.9
**1f**	NO_2	2600	1.58	0.608	0.58	-0.024	4.1

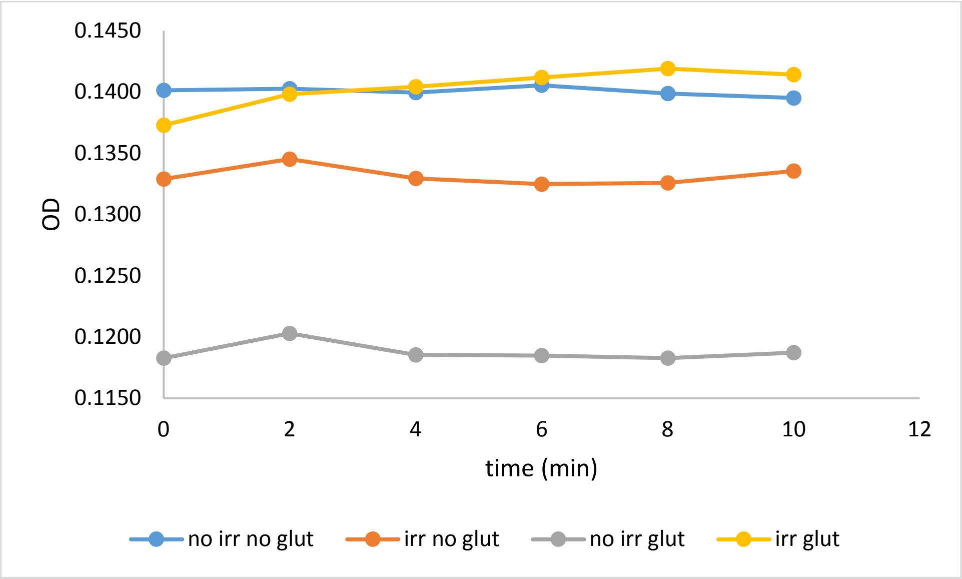

Supplementary Figure 88. *Stability of ~30 μM ITI **1a** in in PBS (1.7% DMSO), followed by at the absorption maximum at 439 nm, subjected to 400 nm light irradiation and/or 10 mM glutathione. A control was done without irradiation and glutathione.*

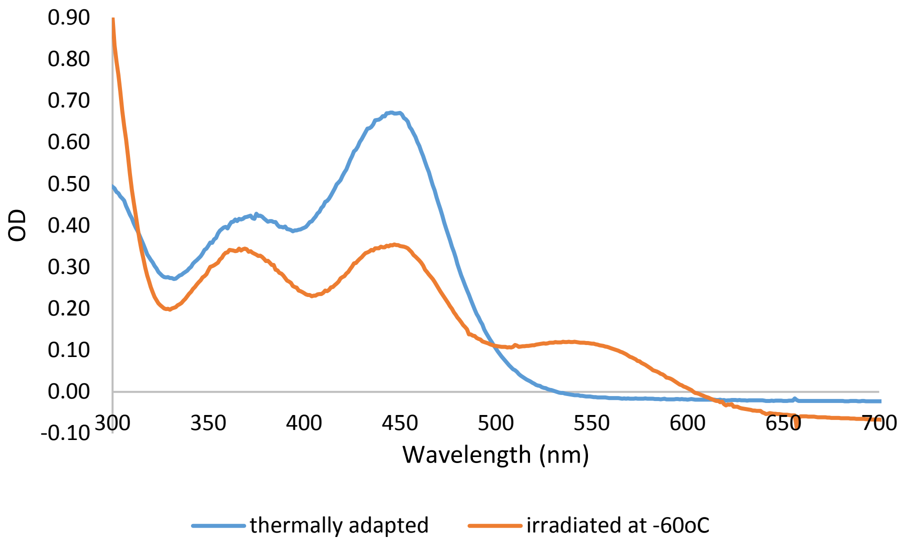

Supplementary Figure 89. *Absorption spectra of 100 μM **1b** in MeOH, thermally adapted and switched with 400 nm light, while cooled at -60 °C acetone.*

Supplementary References:

1. Mukherjee, C., Kamila, S. & De, A. Application of directed metalation in synthesis. Part 4: Expedient synthesis of substituted benzo[b]thiophene and naphthothiophene. *Tetrahedron* **59,** 4767–4774 (2003).
2. Soeta, T., Shitaya, S., Okuno, T., Fujinami, S. & Ukaji, Y. Efficient synthesis of benzothiophenes by [4+1] cycloaddition of 2-mercaptobenzaldehyde derivatives with isocyanides. *Tetrahedron* **72,** 7901–7905 (2016).
3. Prlj, A., Sandoval-Salinas, M. E., Casanova, D., Jacquemin, D. & Corminboeuf, C. Low-Lying ππ* States of Heteroaromatic Molecules: A Challenge for Excited State Methods. *J. Chem. Theory Comput.* **12,** 2652–2660 (2016).
4. Christiansen, O., Koch, H. & Jørgensen, P. The second-order approximate coupled cluster singles and doubles model CC2. *Chem. Phys. Lett.* **243,** 409–418 (1995).
5. Wormit, M. *et al.* Investigating excited electronic states using the algebraic diagrammatic construction (ADC) approach of the polarisation propagator. *Mol. Phys.* **112,** 774–784 (2014).
6. Perdew, J. P., Ernzerhof, M. & Burke, K. Rationale for mixing exact exchange with density functional approximations. *J. Chem. Phys.* **105,** 9982–9985 (1996).
7. J. P. Perdew, K. Burke, M. E. Generalized gradient approximation made simple. *Phys. Rev. Lett.* **77,** 3865–3868 (1996).
8. Jamróz, M. H., Dobrowolski, J. C. & Brzozowski, R. Vibrational modes of 2,6-, 2,7-, and 2,3-diisopropylnaphthalene. A DFT study. *J. Mol. Struct.* **787,** 172–183 (2006).
9. Ammann, C., Meier, P. & Merbach, A. A simple multinuclear NMR thermometer. *J. Magn. Reson.* **46,** 319–321 (1982).
